# Supplementary material for: Monoterpenoid aryl hydrocarbon receptor allosteric antagonists protect against ultraviolet skin damage in female mice
Source: Nat Commun. 2023 May 11;14:2728. doi: 10.1038/s41467-023-38478-6 (PMC10174618; doi:10.1038/s41467-023-38478-6)
Supplement: Supplementary file 1 — Supplementary Information [file 41467_2023_38478_MOESM1_ESM.pdf]

MONOTERPENOID ARYL HYDROCARBON RECEPTOR ALLOSTERIC ANTAGONISTS PROTECT AGAINST ULTRAVIOLET SKIN DAMAGE IN FEMALE MICE

Karolína Ondrová[1], Iveta Zůvalová[1], Barbora Vyhlídalová[1], Kristýna Krasulová[1], Eva Miková[1], Radim Vrzal[1], Petr Nádvorník[1], Binod Nepal[2], Sandhya Kortagere[2], Martina Kopečná[3], David Kopečný[3], Marek Šebela[4], Fraydoon Rastinejad[5], Hua Pu[5], Miroslav Soural[6], Katharina Maria Rolfes[7], Thomas Haarmann-Stemmann[7], Hao Li[8], Sridhar Mani[8]*, Zdeněk Dvořák[1]*

[1]*Department of Cell Biology and Genetics, Faculty of Science, Palacký University, Šlechtitelů 27, 783 71 Olomouc, Czech Republic*
[2]*Department of Microbiology & Immunology, Drexel University College of Medicine, Philadelphia, PA 19129, U.S.A.*
[3]*Department of Experimental Biology, Faculty of Science, Palacký University, Šlechtitelů 27, 783 71 Olomouc, Czech Republic*
[4]*Department of Biochemistry, Faculty of Science, Palacký University, Šlechtitelů 27, 783 71 Olomouc, Czech Republic*
[5]*Target Discovery Institute Nuffield Department of Medicine Research Building Brasenose College University of Oxford, Old Road Campus, Oxford, OX3 7FZ UK*
[6]*Department of Organic Chemistry, Faculty of Science, Palacký University, 17. Listopadu 12, 771 46 Olomouc, Czech Republic*
[7]*IUF-Leibniz-Research Institute for Environmental Medicine, 40225, Düsseldorf, Germany*
[8]*Department of Medicine, Molecular Pharmacology, and Genetics, Albert Einstein College of Medicine, Bronx, NY 10461, U.S.A.*

Corresponding authors*: Zdeněk Dvořák, Ph.D., D.Sc.
Department of Cell Biology and Genetics
Faculty of Science, Palacky University Olomouc
Slechtitelu 27; 783 71 Olomouc; Czech Republic
E: moulin@email.cz
T: +420-58-5634903 F: +420-58-5634901

Sridhar Mani, M.D.
Department of Genetics and Department of Medicine
Albert Einstein College of Medicine
Bronx, NY 10461, U.S.A.
E: sridhar.mani@einstein.yu.edu

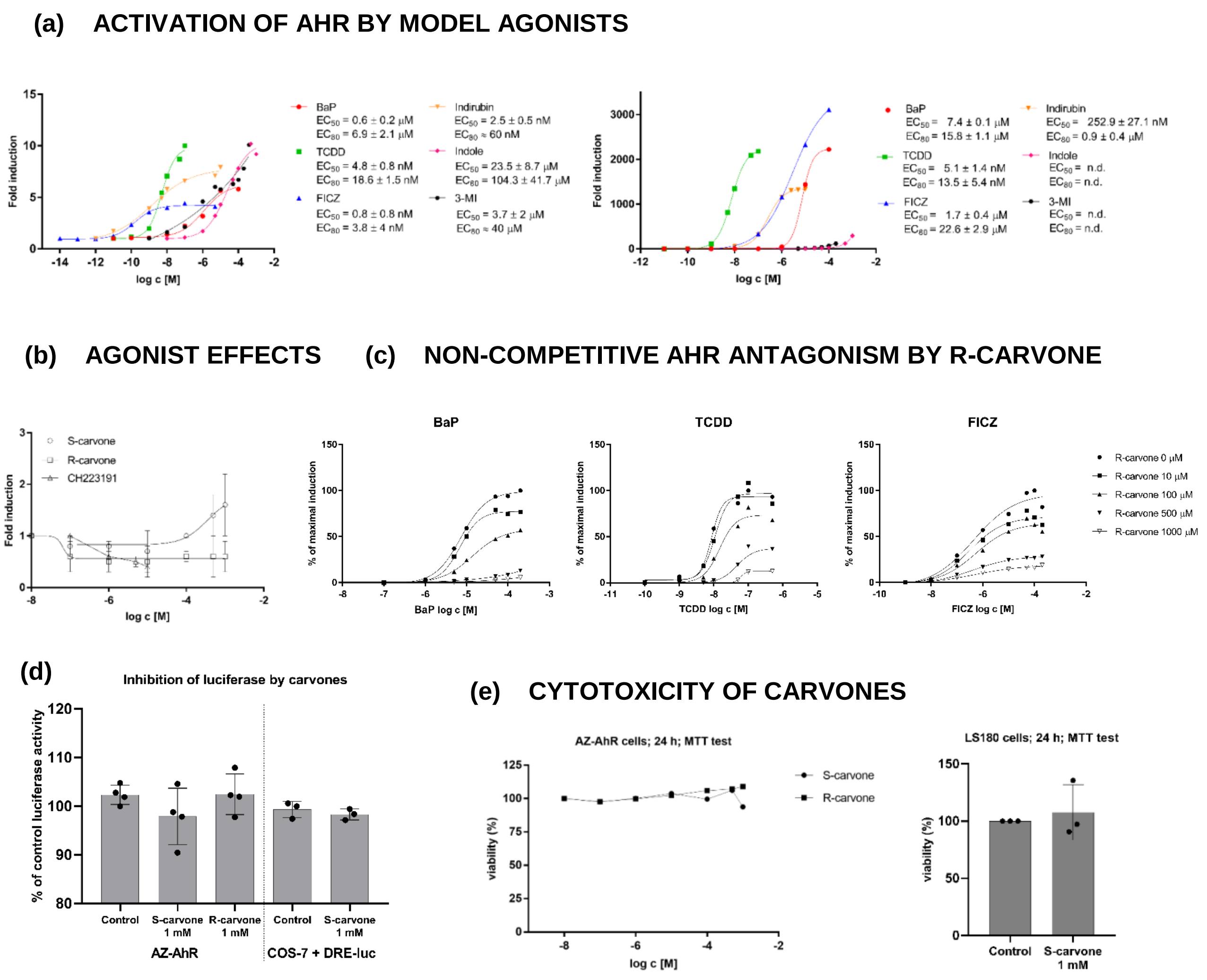

Figure S1. Noncompetitive antagonism of AhR by S-carvone. A reporter gene assay was carried out in stably transfected AZ-AHR cells incubated for 4 h and 24 h with the tested compounds. Incubations and measurements were performed in quadruplicate (technical replicates). **(A)** Activation of AhR by model agonists, including TCDD, BaP, FICZ, indirubin, indole, and 3-MI (EC_{50} and EC_{80} values indicated in the graph). Experiments were performed in three independent cell passages (n=3). Representative plots from one experiment are shown. **(B)** Effects of carvones and CH223191 on basal AhR activity after 24 h of incubation. Data are shown as mean ± SD from three independent cell passages (n=3) **(C)** Noncompetitive antagonism of R-carvone after 24 h: combined incubations with a fixed concentration of R-carvone and increasing concentrations of AhR agonists. Experiments were performed in two independent cell passages (n=2). Representative plots from one experiment are shown. **(D)** Inhibition of luciferase catalytic activity: Stably transfected AZ-AHR cells and COS-7 cells transiently transfected with DRE-luc reporter plasmid were incubated for 24 h

with 20 nM TCDD. Cells were lysed and the lysate containing luciferase was incubated for 30 min with carvones (1000 μM) or vehicle (control). Bar graph shows percentage of luciferase activity relative to control (vehicle). Data are presented as mean ± SD from three consecutive cell passages (n=3), and the treatments were done in triplicates (technical replicates). **(E)** Cell viability – MTT test: AZ-AHR and LS180 Cells were incubated with carvones for 24 h and an MTT test was performed. Treatments were performed in triplicates. The data mean were from two independent cell passages (n=2) and they are expressed as the percentage of control cell viability. Abbreviations: BaP, benzo[*a*]pyrene; FICZ, 6-formylindolo[3,2-*b*]carbazole; 3-MI, 3-methylindole; TCDD, 2,3,7,8-tetrachlorodibenzo-*p*-dioxin. Source data are provided as a Source Data file.

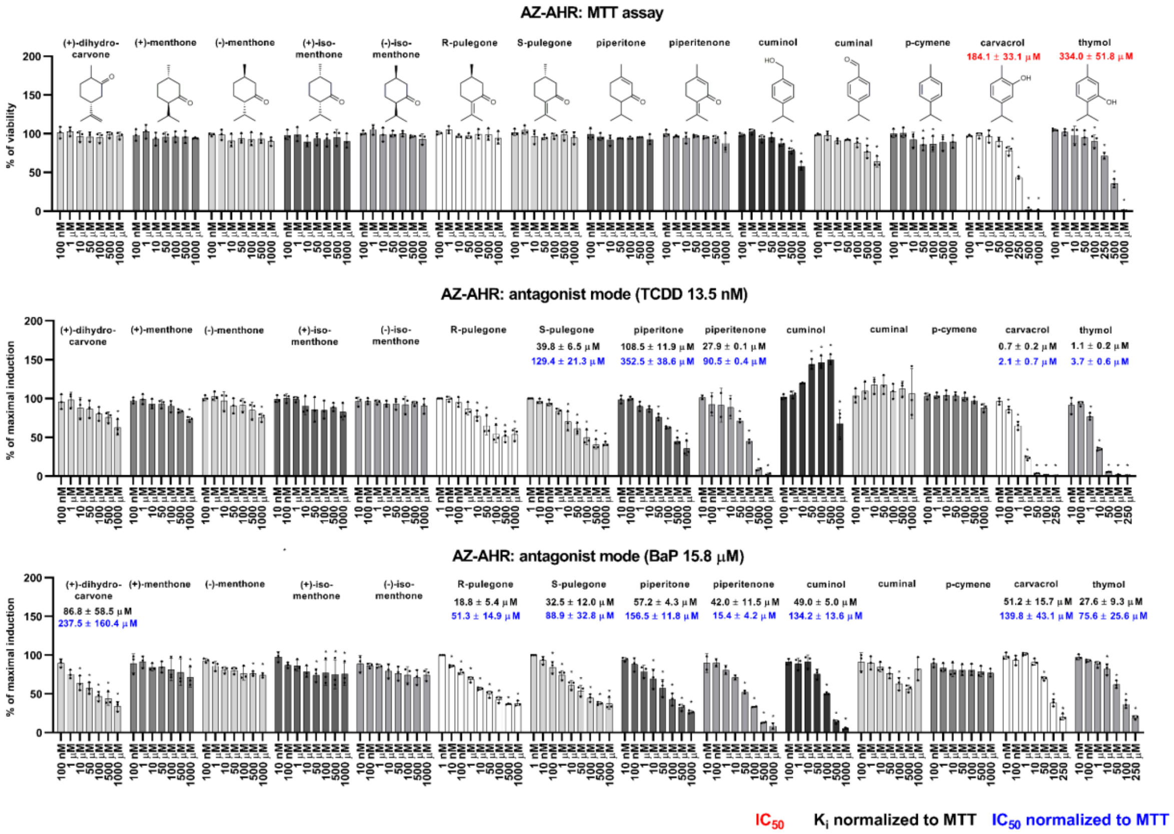

Figure S2. AhR antagonist effects of monoterpenoids. A reporter gene assay and MTT test were carried out in stably transfected AZ-AHR cells incubated for 24 h with the tested compounds in combination with vehicle (DMSO) or model AhR agonists TCDD and BaP . Data are presented as mean ± SD from three independent cell passages (n=3). The significance was calculated using 2way ANOVA multiple comparison test, and * in the graph refers to $p<0.05$. Incubations and measurements were performed in quadruplicate (technical replicates). **(top row)** Cell viability by MTT test. **(middle and bottom rows)** Antagonist effects of tested compounds against model AhR agonists. K_i values are inserted in the graphs, where allowed to calculate. Abbreviations: BaP, benzo[*a*]pyrene; TCDD, 2,3,7,8-tetrachlorodibenzo-*p*-dioxin. Source Data are provided as a Source Data file.

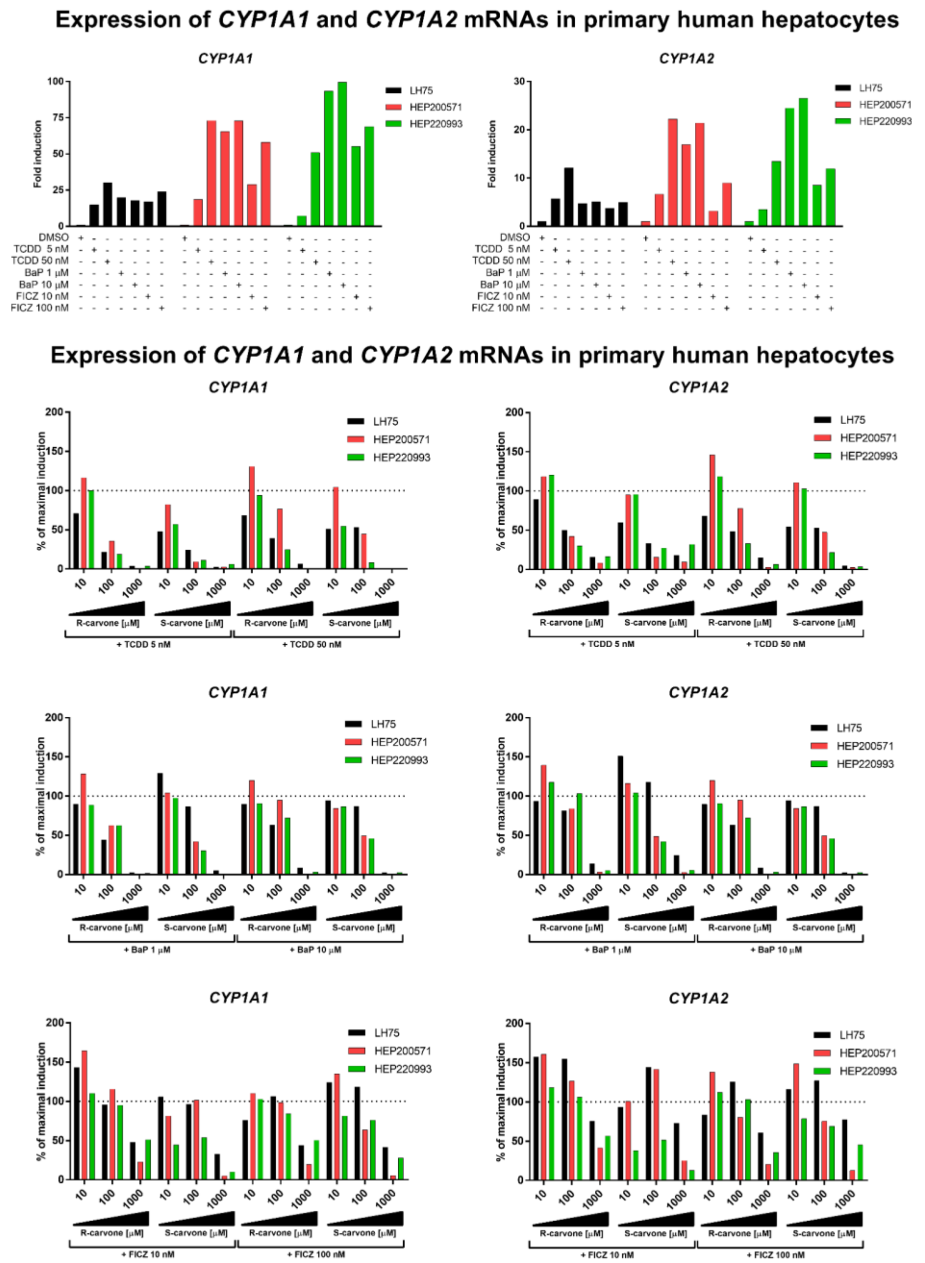

Figure S3. Downregulation of *CYP1A1* and *CYP1A2* mRNAs in primary cultures of human hepatocytes. Human hepatocyte cultures from three tissue donors (LH75, HEP200571, HEP220993) were incubated for 24 h with carvones (10 μM, 100 μM, 1000 μM) in the presence of the AhR agonists TCDD (5 nM, 50 nM), BaP (1 μM, 10 μM) and FICZ (10 nM, 100 nM). RT-PCR quantified *CYP1A* mRNAs. **(A)** Fold induction of *CYP1A* genes by AhR agonists. **(B)** Percentage of *CYP1A* maximal induction by model agonists in the presence of carvones. The data were normalized using *GAPDH* as a housekeeping gene. Abbreviations: BaP, benzo[*a*]pyrene; CYP1A1/2, cytochrome P450 1A1/2; FICZ, 6-formylindolo[3,2-*b*]carbazole; TCDD, 2,3,7,8-tetrachlorodibenzo-*p*-dioxin. Source data are provided as a Source Data file.

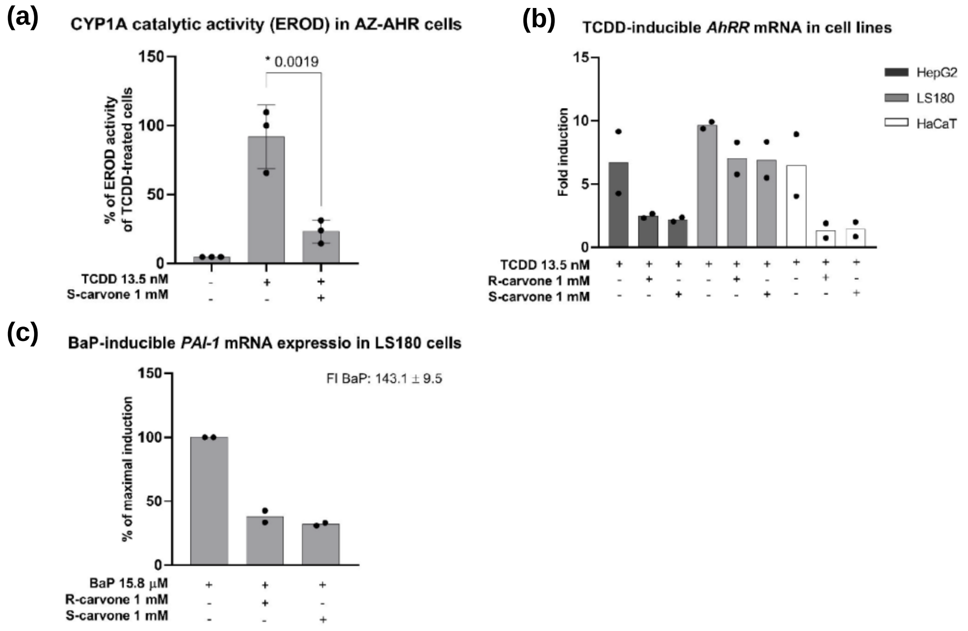

Figure S4. Downregulation of AhR target genes by carvones. (A) Downregulation of 7-ethoxyresorufin-*O*-deethylase EROD by S-carvone in hepatoma cells. The catalytic activity of EROD was measured using a fluorescent substrate in AZ-AHR cells preincubated for 24 h with vehicle (DMSO; 0.1% v/v), TCDD (13.5 nM) and/or a mixture of S-carvone (1 mM) + TCDD (13.5 nM). Incubations and measurements were performed in triplicate (technical replicates). The data are the mean ± S.D. from three consecutive cell passages (n=3) and are expressed as the percentage of fluorescence in TCDD-treated cells; The significance was calculated using 1way ANOVA multiple comparison test, and *p*-values are indicated in the graph. **(B)** Downregulation of *AhRR* by carvones. RT-PCR analyses of *AhRR* mRNA; LS180, HepG2, and HaCaT cells were incubated for 24 h with TCDD (13.5 nM) in the presence or absence of carvones (1 mM). The results are expressed as fold induction per vehicle-treated cells. The data are the mean from two consecutive cell passages (n=2). The results were normalized using *GAPDH* as a housekeeping gene. **(C)** Effects of carvones on noncanonical AhR signaling. RT-PCR analyses of *PAI-1* mRNA; LS180 cells were incubated for 24 h with BaP (15.8 μM) in the presence or absence of carvones (1 mM). The results are expressed relative to BaP in the absence of carvones (100%). Data are the mean from two consecutive cell passages (n=2). The results were normalized using *GAPDH* as a housekeeping gene. The absolute value of *PAI-1* mRNA fold induction (F.I.) by BaP is indicated in the text inserted in a bar graph. Abbreviations: AhRR, AhR repressor; BaP, benzo[*a*]pyrene; CYP1A1, cytochrome P450 1A1; EROD, 7-ethoxyresorufin-*O*-deethylase; PAI-1, plasminogen activator inhibitor 1; TCDD, 2,3,7,8-tetrachlorodibenzo-*p*-dioxin. Source data are provided as a Source Data file.

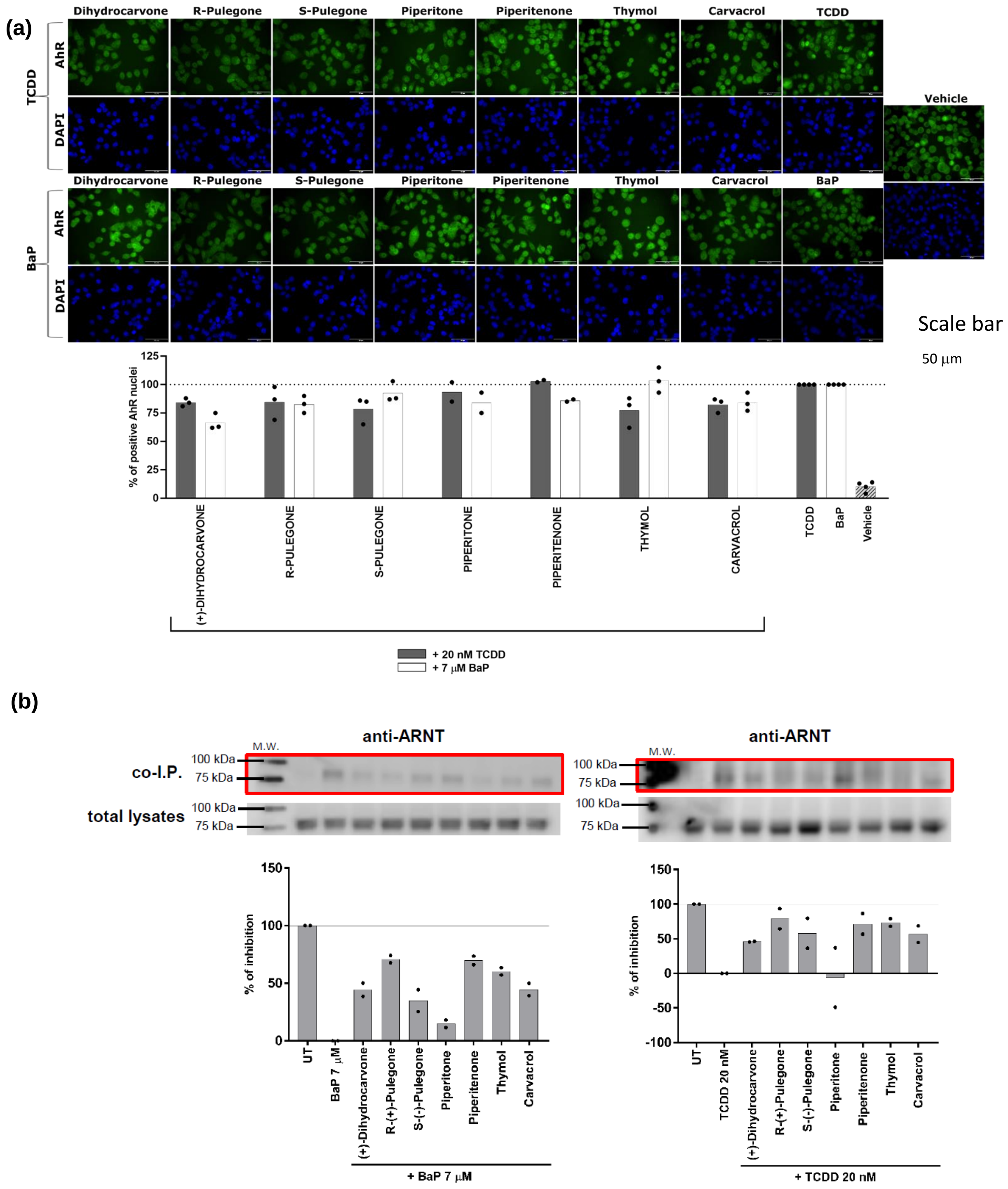

Figure S5. Nuclear translocation and heterodimerization of AhR by monoterpenoids. LS180 cells were incubated for 90 min with tested compounds (1000 μM) in combination with vehicle (0.1% DMSO) or AhR agonists TCDD (20 nM) and BaP (7 μM). **(A)** The nuclear translocation of AhR: Microscopic specimens from cells were prepared using Alexa Fluor 488-labeled primary antibody against AhR and DAPI. AhR was visualized and evaluated using a fluorescence microscope. Experiments were performed in two consecutive cell passages (n=2) with all tested compounds in duplicate. Representative images are shown.

Quantitation of AhR positive nuclei is displayed in a bar graph. Scale bar = 50 μM. **(B)** Protein coimmunoprecipitation – formation of AhR-ARNT heterodimer: Representative immunoblots of immunoprecipitated protein eluates and total cell lysates are shown. Data are mean from two consecutive cell passages (n=2). Abbreviations: BaP, benzo[*a*]pyrene; TCDD, 2,3,7,8-tetrachlorodibenzo-*p*-dioxin. Source data are provided as a Source Data file.

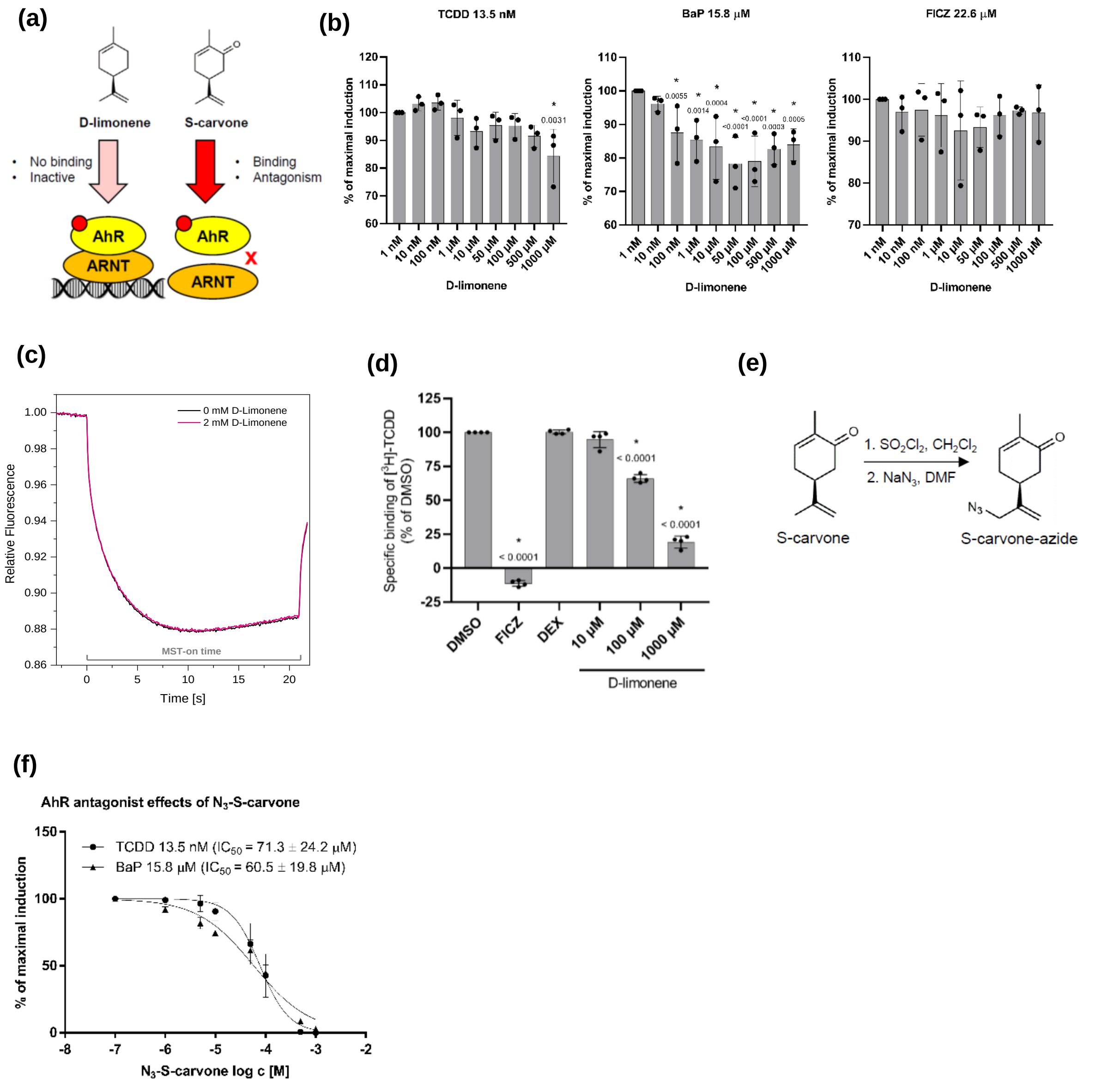

Figure S6. Interaction of D-limonene and N3-S-carvone with AhR. (A) Chemical structures of S-carvone and D-limonene and their schematic interaction with AhR. **(B)** A reporter gene assay was carried out in AZ-AHR cells incubated for 24 h with increasing concentrations of D-limonene in combination with the AhR agonists TCDD (13.5 nM), BaP (15.8 µM) and FICZ (22.6 µM). Incubations and measurements were performed in quadruplicate (technical replicates). The bar graph shows the percentage of maximal induction attained by a model AhR agonist. Data are the mean ± S.D. from three consecutive cell passages (n=3). The significance was calculated using 2way ANOVA multiple comparison test, and *p*-values are indicated in the graph. **(C)** Microscale thermophoresis using coexpressed His-AhR + FLAG-Arnt incubated with vehicle or 1 mM D-limonene. **(D)** Competitive radioligand binding assay: Cytosolic protein from Hepa1c1c7 cells was

incubated with D-limonene (10 μM, 100 μM, 1000 μM), FICZ (10 nM), DEX (100 nM; negative control) or DMSO (0.1% V/V; corresponding to *specific binding of [3H]-TCDD = 100%*) in the presence of 2 nM [^{3}H]- TCDD. Specific binding of [^{3}H]-TCDD was determined to be the difference between total and nonspecific (200 nM; 2,3,7,8-tetrachlorodibenzofuran) reactions. The significance was calculated using 1way ANOVA multiple comparison test, and *p*-values are indicated in the graph. Four independent experiments were performed (n=4), and the incubations and measurements were performed in triplicate in each experiment (technical replicates). The error bars represent the mean ± S.D. **(E)** Chemical synthesis of N_3-S-carvone. **(F)** Reporter gene assay in AZ-AHR cells incubated for 24 h with N_3-S-carvone in combination with TCDD (13.5 nM) and BaP (15.8 μM). Incubations were performed in quadruplicate (technical replicates). The bar graph shows the percentage of maximal induction attained by a model AhR agonist. Data are the mean ± S.D. from three consecutive cell passages (n=3). Abbreviations: BaP, benzo[*a*]pyrene; DEX, dexamethasone; FICZ, 6-formylindolo[3,2-*b*]carbazole; TCDD, 2,3,7,8-tetrachlorodibenzo-*p*-dioxin. Source data are provided as a Source Data file.

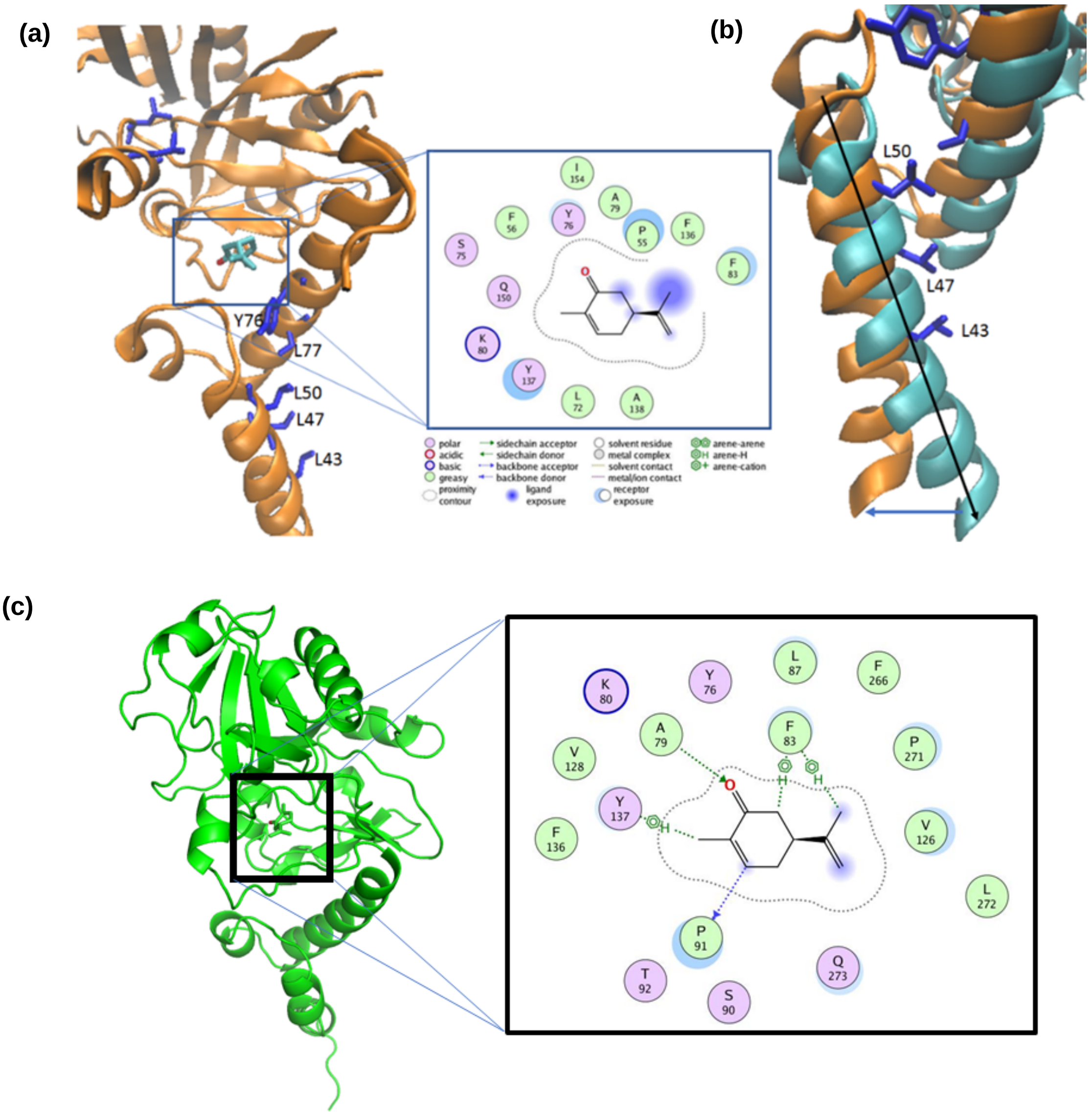

Figure S7. Molecular Docking and simulations of S-Carvone to AhR. A) S-carvone (licorice stick and colored atom type, carbon = cyan and oxygen = red) binds to a site proximal to the heterodimerization interface of AhR (depicted as orange ribbons with interface residues shown as blue licorice sticks and labeled) with residues from helices α1 and α2 contributing to the binding interactions (center panel). **B)** The complex of S-carvone with AhR was simulated to 10 ns and the resulting structure (cyan ribbons) was superimposed on to the original complex. The binding of S-carvone to this site also leads to conformational changes in the α1 and α2 helices (new positions shown as cyan ribbons), thereby disrupting the formation of the AhR-ARNT interface**. C)** The complex of S-carvone with AhR was simulated to 250 ns and the conformation of the complex is shown as green ribbons. The binding mode of S-carvone in displayed in the center panel. The binding of S-carvone to this site also leads to unwinding of the α1 helix which may be further disrupt the AhR-ARNT interface.

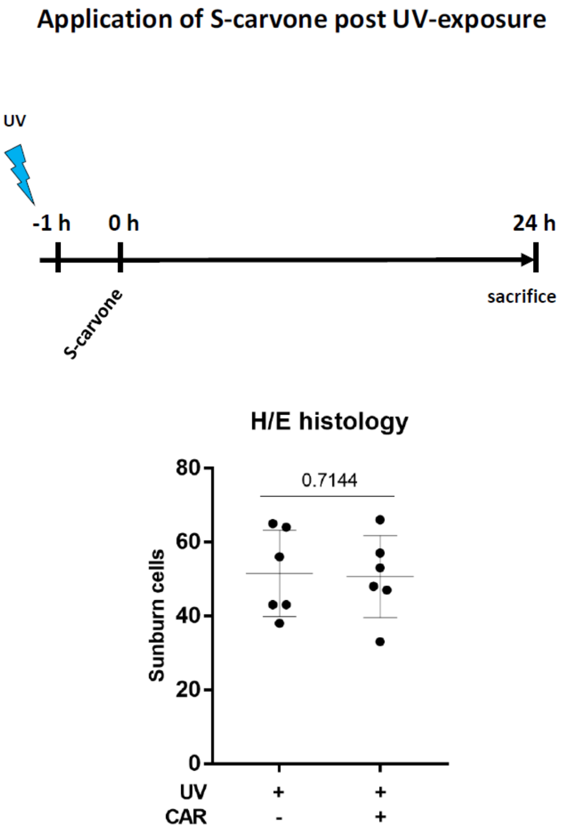

Figure S8. UV-burned cells in mouse ears treated with S-carvone post-UV exposure. S-carvone and UV irradiation were applied to C57BL/6 mouse auricles as described in the Methods section and as shown in a scheme. The scatter plot shows the count of UV-burned cells in the left and right ears of an individual mouse (n=6). Data are shown as mean ± SD. The significance was calculated using paired two-tailed *t*-test. P-value is indicated in the graph. Abbreviations: CAR, S-carvone; H/E, hematoxylin/eosin stain. Source data are provided as a Source Data file.

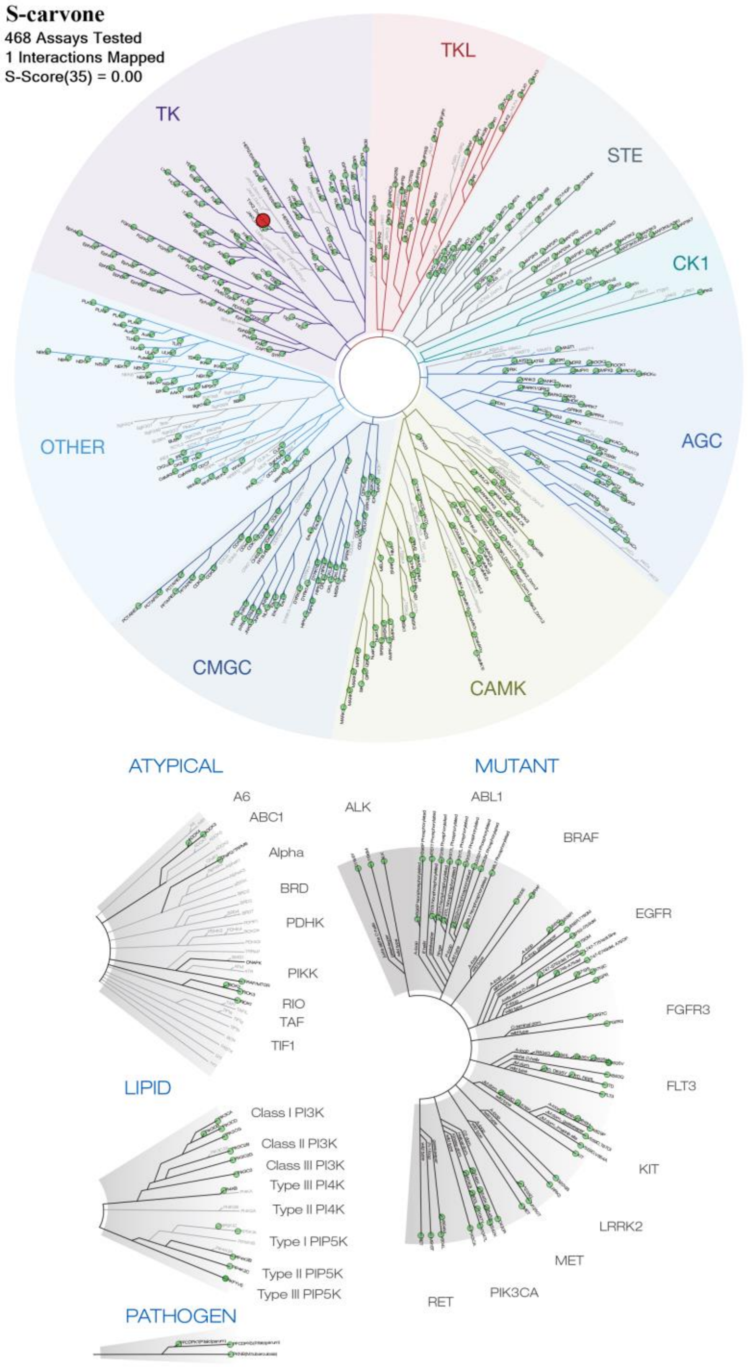

Figure S9. KINOMEscan^{TM} profiling: The interaction between 100 μM S-carvone and 468 human protein kinases, employing KINOMEscan^{TM} (scanMAX assay), a proprietary active site-directed competition binding assay. A high-resolution interaction map is shown. Source data are provided as a Source Data file.

GENE	primers & probes	supplier
GAPDH	probe: GAPDH-UPL60<br>fw: CTCTGCTCCTCCTGTTCGAC<br>rev: ACGACCAAATCCGTTGACTC	Universal Probes Library, Roche
CYP1A1	probe: CYP1A1-UPL33<br>fw: CCAGGCTCCAAGAGTCCA<br>rev: GATCTTGGAGGTGGCTGCT	
GAPDH	fw: AGGTGAAGGTCGGAGTCA<br>rev: GGTCATTGATGGCAACAA	Eurofins Genomics
VEGF	fw: TGCAAAAACACAGACTCGCG<br>rev: TGTCACATCTGCAAGTACGTTCG	
GAPDH	probe: [6FAM]ACTAACCCTGCGCTCCTGCCTCGAT[OQA]<br>fw: GAAGGAAATGAATGGGCAGC<br>rev: TCTAGGAAAAGCATCACCCG	KiCqStart®
PAI-1	probe: [6FAM]GTGGCCTCCTCATCCACAGCTGTCA[OQA]<br>fw:GCTGCAGAAAGTGAAGATCG<br>rev: GTCCATGATGATCTCCTCGG	
AhRR	probe: [6FAM]AAACCCAGAGCAGACACCGCAGCCA[OQA]<br>fw: GAGATGAAAATGAGGAGCGC<br>rev: TTTTACTTTTGCATCCGCGG	
mRplp0	fw: CGTCCTCGTTGGAGTGACAT<br>rev: TAGTTGGACTTCCAGGTCGC	Thermo Fischer Scientific
mCXCL5	fw: TGCCCTACGGTGGAAGTCAT<br>rev: AGCTTTCTTTTTGTCACTGCCC	
mCYP1A1	fw: CTCTTCCCTGGATGCCTTCAA<br>rev: GGATGTGGCCCTTCTCAAATG	
mIL-1β	fw: CAGGCAGGCAGTATCACTCA<br>rev: AGGTGCTCATGTCCTCATCC	

Table S1. Primers and probes used in real-time polymerase chain reaction RT-PCR.

% of inhibition		R-CARVONE					S-CARVONE				
		0 μM	10 μM	100 μM	500 μM	1000 μM	0 μM	10 μM	100 μM	500 μM	1000 μM
FICZ	100 nM	0%	19%	43%	71%	82%	0%	15%	29%	44%	77%
	1 μM	0%	16%	33%	70%	82%	0%	13%	25%	55%	82%
	10 μM	0%	20%	31%	70%	82%	0%	17%	28%	57%	88%
	50 μM	0%	24%	32%	71%	82%	0%	21%	30%	55%	90%
	100 μM	0%	24%	32%	71%	81%	0%	22%	32%	53%	91%
	200 μM	0%	24%	32%	70%	81%	0%	23%	33%	50%	91%
BaP	5 μM	0%	24%	63%	94%	97%	0%	18%	45%	89%	95%
	10 μM	0%	16%	61%	95%	97%	0%	19%	44%	92%	97%
	50 μM	0%	19%	49%	93%	97%	0%	19%	37%	90%	96%
	100 μM	0%	20%	45%	91%	96%	0%	19%	33%	87%	95%
	200 μM	0%	21%	43%	87%	94%	0%	18%	31%	84%	93%
TCDD	5 nM	0%	12%	56%	98%	100%	0%	39%	60%	93%	100%
	10 nM	0%	23%	59%	96%	100%	0%	14%	55%	89%	99%
	50 nM	0%	2%	29%	80%	97%	0%	8%	24%	62%	97%
	100 nM	0%	3%	26%	69%	88%	0%	8%	20%	53%	90%
	500 nM	0%	4%	25%	62%	87%	0%	8%	19%	46%	88%

Table S2. Quantitative analysis of S/R-carvones in reporter gene assay against uncompetitive antagonism. A reporter assay was carried out in stably transfected AZ-AHR cells and then incubated for 24 h with a fixed concentration of carvones combined with increasing concentrations of AhR agonists. Experiments were performed in two independent cell passages (n=2). Incubations and measurements were performed in quadruplicate (technical replicates). The percent inhibition by carvones (10 μM; 100 μM; 500 μM; 1000 μM) was calculated for all tested agonists as follows:

% Inhibition C_x = 100*(CARVONE C_0 - CARVONE C_x)/CARVONE C_0

		# of cells	# of fields of vision	# of AhR positive nuclei	% of AhR positive nuclei	Relative ligand efficiency (%)
**DMSO**	Exp #1	359	3	16	4.5	n.a.
	Exp #2	438	4	6	1.3	n.a.
**TCDD 20 nM**	Exp #1	550	5	235	43.1	100
	Exp #2	453	4	198	43.6	100
**BaP 7 µM**	Exp #1	446	4	143	31.9	100
	Exp #2	486	4	158	32.6	100
**FICZ 8 nM**	Exp #1	494	4	176	35.3	100
	Exp #2	470	4	188	40.9	100
**R-carvone 1 mM**	Exp #1	445	4	18	4.0	n.a.
	Exp #2	429	4	2	0.5	n.a.
**R-carvone + TCDD**	Exp #1	437	4	212	48.6	90
	Exp #2	491	4	193	39.5	97
**R-carvone + BaP**	Exp #1	454	4	144	32.0	101
	Exp #2	396	4	169	42.3	107
**R-carvone + FICZ**	Exp #1	481	4	191	40.0	109
	Exp #2	496	5	191	38.7	102
**S-carvone 1 mM**	Exp #1	570	5	23	4.0	n.a.
	Exp #2	428	4	6	1.5	n.a.
**S-carvone + TCDD**	Exp #1	485	4	233	48.6	99
	Exp #2	462	4	157	34.3	79
**S-carvone + BaP**	Exp #1	450	4	163	36.3	116
	Exp #2	519	4	146	28.4	92
**S-carvone + FICZ**	Exp #1	460	4	194	42.4	111
	Exp #2	428	4	154	36.3	81

Table S3. Carvones do not influence the nuclear translocation of AhR. LS180 cells were incubated for 90 min with carvones (1000 mM) in combination with vehicle (0.1% DMSO) or the AhR agonists TCDD (20 nM), BaP (7 µM), and FICZ (8 nM). Microscopic specimens were prepared using Alexa Fluor 488-labeled primary antibody against AhR and DAPI. AhR was visualized and evaluated using a fluorescence microscope. Experiments were performed in two consecutive cell passages (n=2), with all tested compounds in duplicate. The table shows the total and AhR-positive cell counts.
