# Supplementary material for: Monoterpenoid aryl hydrocarbon receptor allosteric antagonists protect against ultraviolet skin damage in female mice
Source: Nat Commun. 2023 May 11;14:2728. doi: 10.1038/s41467-023-38478-6 (PMC10174618; doi:10.1038/s41467-023-38478-6)
Supplement: Supplementary file 3 — Reporting Summary [file 41467_2023_38478_MOESM3_ESM.pdf]

nature portfolio

Corresponding author(s): Zdenek DVORAK, Sridhar MANI

Last updated by author(s): Apr 28, 2023

Reporting Summary

Nature Portfolio wishes to improve the reproducibility of the work that we publish. This form provides structure for consistency and transparency in reporting. For further information on Nature Portfolio policies, see our Editorial Policies and the Editorial Policy Checklist.

Statistics

For all statistical analyses, confirm that the following items are present in the figure legend, table legend, main text, or Methods section.

n/a	Confirmed	
☐	☒	The exact sample size (*n*) for each experimental group/condition, given as a discrete number and unit of measurement
☐	☒	A statement on whether measurements were taken from distinct samples or whether the same sample was measured repeatedly
☐	☒	The statistical test(s) used AND whether they are one- or two-sided<br>*Only common tests should be described solely by name; describe more complex techniques in the Methods section.*
☒	☐	A description of all covariates tested
☒	☐	A description of any assumptions or corrections, such as tests of normality and adjustment for multiple comparisons
☐	☒	A full description of the statistical parameters including central tendency (e.g. means) or other basic estimates (e.g. regression coefficient) AND variation (e.g. standard deviation) or associated estimates of uncertainty (e.g. confidence intervals)
☐	☒	For null hypothesis testing, the test statistic (e.g. F, t, r) with confidence intervals, effect sizes, degrees of freedom and P value noted<br>*Give P values as exact values whenever suitable.*
☒	☐	For Bayesian analysis, information on the choice of priors and Markov chain Monte Carlo settings
☒	☐	For hierarchical and complex designs, identification of the appropriate level for tests and full reporting of outcomes
☒	☐	Estimates of effect sizes (e.g. Cohen's *d*, Pearson's *r*), indicating how they were calculated

Our web collection on statistics for biologists contains articles on many of the points above.

Software and code

Policy information about availability of computer code

Data collection	Sally Sue™ Simple Western System (ProteinSimple™), Compass Software version 2.6.5.0 (ProteinSimple™); Light Cycler® 480 Instrument II (Roche), LightCycler® 480 Software version1.5; Fluorescence microscope IX73 (Olympus, Japan), OLYMPUS cellSens Standard version 1.17; C-DiGit® Blot Scanner (LI-COR Biotechnology), Image Studio Digit version 3.1; Microplate reader Infinite M200 (TECAN, Austria), i-control Software version 1.11; KINOMEscanTM screening platform (Eurofins DiscoverX, San Diego, CA, USA); Eurofins Panlabs Discovery Services Taiwan (New Taipei City, Taiwan) and Eurofins Cerep SA (Poitiers, France); Reaction Biology Corp. (Malvern, PA, USA); ViiA7 Real Time PCR System (Thermo Fisher Scientific, Waltham, MA 02451); MALDI-TOF/TOF MS and MSMS on an ultrafleXtreme instrument equipped with a Smartbeam II Nd:YAG laser (Bruker Daltonik, Bremen, Germany).
Data analysis	GraphPad Prism 8 for Windows version 8.2.1 (GraphPad Software, La Jolla, CA, USA); GraphPad Prism 9 for Windows version 9.4.1 (GraphPad Software, La Jolla, CA, USA); Microsoft Excel 2016 standard 32bit 16.0.5378.1000; MO Affinity analysis software for Monolith NT.115 instrument version 2.3 (NanoTemper Technologies); Compass Software version 2.6.5.0 (ProteinSimple™); QuantStudio Real-Time PCR Software version 1.3 (Thermo Fisher); flexAnalysis version 3.4 and BioTools version 3.1 (Bruker Daltonik); ProteinScape version 3.1 (Bruker Daltonik); Mascot Server version 2.4 (Matrix Science, London, UK), PEAKS Studio version X (Bioinformatics Solutions, Waterloo, ON, Canada).

For manuscripts utilizing custom algorithms or software that are central to the research but not yet described in published literature, software must be made available to editors and reviewers. We strongly encourage code deposition in a community repository (e.g. GitHub). See the Nature Portfolio guidelines for submitting code & software for further information.

Data

Policy information about availability of data

All manuscripts must include a data availability statement. This statement should provide the following information, where applicable:

- Accession codes, unique identifiers, or web links for publicly available datasets
- A description of any restrictions on data availability
- For clinical datasets or third party data, please ensure that the statement adheres to our policy

Data are available in publicly accessible repository: https://doi.org/10.5281/zenodo.7764002

Research involving human participants, their data, or biological material

Policy information about studies with human participants or human data. See also policy information about sex, gender (identity/presentation), and sexual orientation and race, ethnicity and racism.

Reporting on sex and gender	The sex of donors was determined solely on the basis of presently available donors at the time of experiments.
Reporting on race, ethnicity, or other socially relevant groupings	The race/ethnicity of donors was determined solely on the basis of presently available donors at the time of experiments.
Population characteristics	Not relevant for current study.
Recruitment	The primary human hepatocytes Hep200571 (male, 77 years, unknown ethnicity) and Hep220993 (female, 76 years, Caucasian) were purchased from Biopredic International (Rennes, France). The primary human hepatocytes LH75 (female, 78 years, Caucasian) were prepared at the Faculty of Medicine, Palacky University Olomouc.
Ethics oversight	The tissue acquisition protocol complied with the regulation issued by the "Ethical Committee of the Faculty Hospital Olomouc, Czech Republic" and Transplantation law #285/2002 Coll (which implies presumed consent from donor).

Note that full information on the approval of the study protocol must also be provided in the manuscript.

Field-specific reporting

Please select the one below that is the best fit for your research. If you are not sure, read the appropriate sections before making your selection.

[x] Life sciences [ ] Behavioural & social sciences [ ] Ecological, evolutionary & environmental sciences

For a reference copy of the document with all sections, see nature.com/documents/nr-reporting-summary-flat.pdf

Life sciences study design

All studies must disclose on these points even when the disclosure is negative.

Sample size	For majority of experiments, n=3 was chosen as the minimal replicate number. The sample size was determined based on the previous experiment and the standards in the field. Due to the high reproducibility and consistency between cell cultures, it was predetermined in the in vitro studies that a sample size of at least n=2 would allow adequate analysis to reach meaningful conclusions from the data (off target analyses, nuclear translocation). For in vivo studies, where higher variance is expected, we used n=6 or n=9, which is in line with common standards governing in vivo studies.
Data exclusions	No data was excluded
Replication	All attempts at replication were successful. The number of repeats and time span differed depending on the particular assay. For most in vitro studies, these were reproduced ("n" stated at each assay) within weeks. Key experiments including reporter gene assays (antagonism) and microscale thermophoresis (binding) were fully reproducible in the period of two years.
Randomization	The nature and principles of in vitro and physical assays do not require randomization. For in vivo experiments, the mice were selected randomly. The left and right ears were exposed to different treatments, thereby providing internal individual controls for comparative treatments, and each animal represents a biological repeat.
Blinding	No blinding was implemented for reported experiments, because the individual assays were carried out by single operator from the start to the end.

Reporting for specific materials, systems and methods

We require information from authors about some types of materials, experimental systems and methods used in many studies. Here, indicate whether each material, system or method listed is relevant to your study. If you are not sure if a list item applies to your research, read the appropriate section before selecting a response.

Materials & experimental systems

n/a	Involved in the study
☐	☒ Antibodies
☐	☒ Eukaryotic cell lines
☒	☐ Palaeontology and archaeology
☐	☒ Animals and other organisms
☒	☐ Clinical data
☒	☐ Dual use research of concern
☒	☐ Plants

Methods

n/a	Involved in the study
☒	☐ ChIP-seq
☒	☐ Flow cytometry
☒	☐ MRI-based neuroimaging

Antibodies

Antibodies used	Anti-CYP1A1 mouse monoclonal antibody (Santa Cruz Biotechnology, sc-393979, A-9, LOT C0217); anti-β-actin mouse monoclonal antibody (Cell Signalling Technology, 3700S, LOT 15, 8H10D10); anti-ARNT 1 mouse monoclonal antibody (Santa Cruz Biotechnology, sc-17812, G-3, LOT B2306); anti-AhR mouse monoclonal antibody (Santa Cruz Biotechnology, sc-133088, A-3, LOT 1718); anti-His-tag mouse monoclonal antibody (Invitrogen, MA1-21315, LOT WH326875); anti-FLAG-tag rabbit monoclonal antibody (Cell Signaling Technology, 14793S, LOT 5); Alexa Fluor 488 labeled anti-AhR mouse monoclonal antibody (Santa Cruz Biotechnology, sc-133088 , LOT AF488); anti-AhR rabbit monoclonal antibody (Cell Signaling Technology, D5S6H, LOT 83200); horse anti-mouse secondary HRP-linked antibody (Cell Signaling Technology, 7076S, LOT 34); goat anti-rabbit secondary HRP-linked antibody (Cell Signaling Technology, 7074P2, LOT 30); goat anti-mouse secondary HRP- linked antibody (Protein Simple, PN 042-205, LOT 86063).
Validation	Anti-CYP1A1 mouse monoclonal antibody (Santa Cruz Biotechnology, sc-393979, A-9, LOT C0217) cited in 20 publications; specific for an epitope mapping between amino acids 479-506 near the C-terminus of CYP1A1 of mouse origin; recommended for detection of CYP1A1 of mouse, rat and human origin by western blot (WB), immunoprecipitation (IP), immunofluorescence (IF) and enzyme-linked immuno sorbent assay (ELISA).<br>Anti-β-actin mouse monoclonal antibody (Cell Signalling Technology, 3700S, LOT 15, 8H10D10) cited in 3611 publications; corresponding to amino-terminal residues of human β-actin, recommended for detection of β-actin of mouse, rat, hamster, monkey, dog and human origin by WB, Immunohistochemistry (IHC) IF and flow cytometry.<br>Anti-ARNT 1 mouse monoclonal antibody (Santa Cruz Biotechnology, sc-17812, G-3, LOT B2306); cited in 9 publications, raised against amino acids 520-692 mapping near the C-terminus of Arnt 1 of human origin; recommended for detection of Arnt 1 of human origin by WB, IP, IF and ELISA.<br>Anti-AhR mouse monoclonal antibody (Santa Cruz Biotechnology, sc-133088, A-3, LOT 1718); cited in 86 publications, raised against amino acids 637-848 of Ah Receptor of human origin; recommended for detection of AhR of mouse, rat and human origin by WB, IP, IF, IHC(P) and ELISA.<br>Anti-His-tag mouse monoclonal antibody (Invitrogen, MA1-21315, LOT WH326875); citated in 179 publications; verified by relative expression to ensure that the antibody binds to the antigen stated; recommended for detection of His-tag by WB, IP, IF, IHC(P) and ELISA.<br>Anti-FLAG-tag rabbit monoclonal antibody (Cell Signaling Technology, 14793S, LOT 5); citated in 855 publications; recommended for detection of DYKDDDDK(Flag)-tag by WB, IP, IF, IHC(P), flow cytometry and Chromatin IP (ChIP).<br>Alexa Fluor 488 labeled anti-AhR mouse monoclonal antibody (Santa Cruz Biotechnology, sc-133088 , LOT AF488); cited in 86 publications; raised against amino acids 637-848 of Ah Receptor of human origin; recommended for detection of AhR of mouse, rat and human origin by WB, IP, IF, IHC(P) and ELISA.<br>Anti-AhR rabbit monoclonal antibody (Cell Signaling Technology, D5S6H, LOT 83200); cited in 38 publications; recommended for detection of AhR by WB, IP and ChIP.

Eukaryotic cell lines

Policy information about cell lines and Sex and Gender in Research

Cell line source(s)	Human hepatoma cells HepG2 (ECACC No. 85011430); intestinal human colon adenocarcinoma cells LS180 (ECACC No. 87021202); mouse hepatoma Hepa1c1 (ECACC No. 95090613); monkey kidney fibroblast cell line COS-7 (ECACC No. 87021302) were purchased from European Collection of Cell Cultures.<br>Human immortalized keratinocytes HaCaT were obtained from German Cancer Research Centre (Heidelberg, Germany).<br>Primary human hepatocytes Hep200571 and Hep220993 were purchased from Biopredic International (Rennes, France).<br>Primary human hepatocytes LH75 were prepared at Faculty of Medicine, Palacky University Olomouc. The tissue acquisition protocol complied the regulation issued by "Ethical Committee of the Faculty Hospital Olomouc, Czech Republic" and Transplantation law #285/2002 Coll.<br>Development and verification of stably transfected gene reporter cell line AZ-AHR was described previously (Novotna et al., 2011).
Authentication	The cell lines were not authenticated.
Mycoplasma contamination	Cells tested negative for Mycoplasma by Mycoplasma Detection Kit-Digital Test v2.0 Cat.No. B39132 (Biotool).
Commonly misidentified lines (See ICLAC register)	No commonly misidentified cell lines were used.

Animals and other research organisms

Policy information about studies involving animals; ARRIVE guidelines recommended for reporting animal research, and Sex and Gender in Research

Laboratory animals	Five-week-old female C57BL/6 mice which were purchased from Jackson Laboratories(Bar Harbor, Maine; # 000664) were co-housed for acclimatization at the vivarium for one weeks prior to experiments. Housing conditions: 14 hour light/ 10 hour dark cycle; temperature: 20-22 °C; humidity: 30-70%; diet: LAB Diet #5058. All clinical inspections were performed by laboratory personnel. On our animal protocol, mice were clinically inspected daily (with particular attention to ear and body skin conditions at 6-8 h intervals within a single day). For irradiation studies, there is potential for ear bleeding, ulceration, and infection. These could result in poor movement or feeding although this was not observed in any mice over a 24 h period of observation. Mice were to be euthanized if they exhibited – poor feeding, ulcerated skin on ears, cachexia, weight loss > 20% of highest basal weight, and poor drinking - mice were to be hydrated for loss of fluids (50-100 microL in 0.9% saline/PBS every 3-4 h to look for signs of reversal), shallow breathing. None of the mice in the study met any criteria for euthanasia. At the end of the experiment, all mice were euthanized by CO2 asphyxiation.
Wild animals	No wild animals were used in the study.
Reporting on sex	The experiments were performed solely in female mice. This is reflected and reported in all finding in the manuscript, including the abstract and title.
Field-collected samples	No field-collected samples were used in the study
Ethics oversight	The experiments were approved by the Institutional Animal Care and Use Committee of the Albert Einstein College of Medicine (New York, NY, USA; Protocol #00001405).

Note that full information on the approval of the study protocol must also be provided in the manuscript.
