# Supplementary material for: Monoterpenoid aryl hydrocarbon receptor allosteric antagonists protect against ultraviolet skin damage in female mice
Source: Nat Commun. 2023 May 11;14:2728. doi: 10.1038/s41467-023-38478-6 (PMC10174618; doi:10.1038/s41467-023-38478-6)
Supplement: Supplementary file 4 — source data [file 41467_2023_38478_MOESM4_ESM.zip › DATA - ONDROVA new/Figure 3/Figure 3B.pptx]

## Slide 1
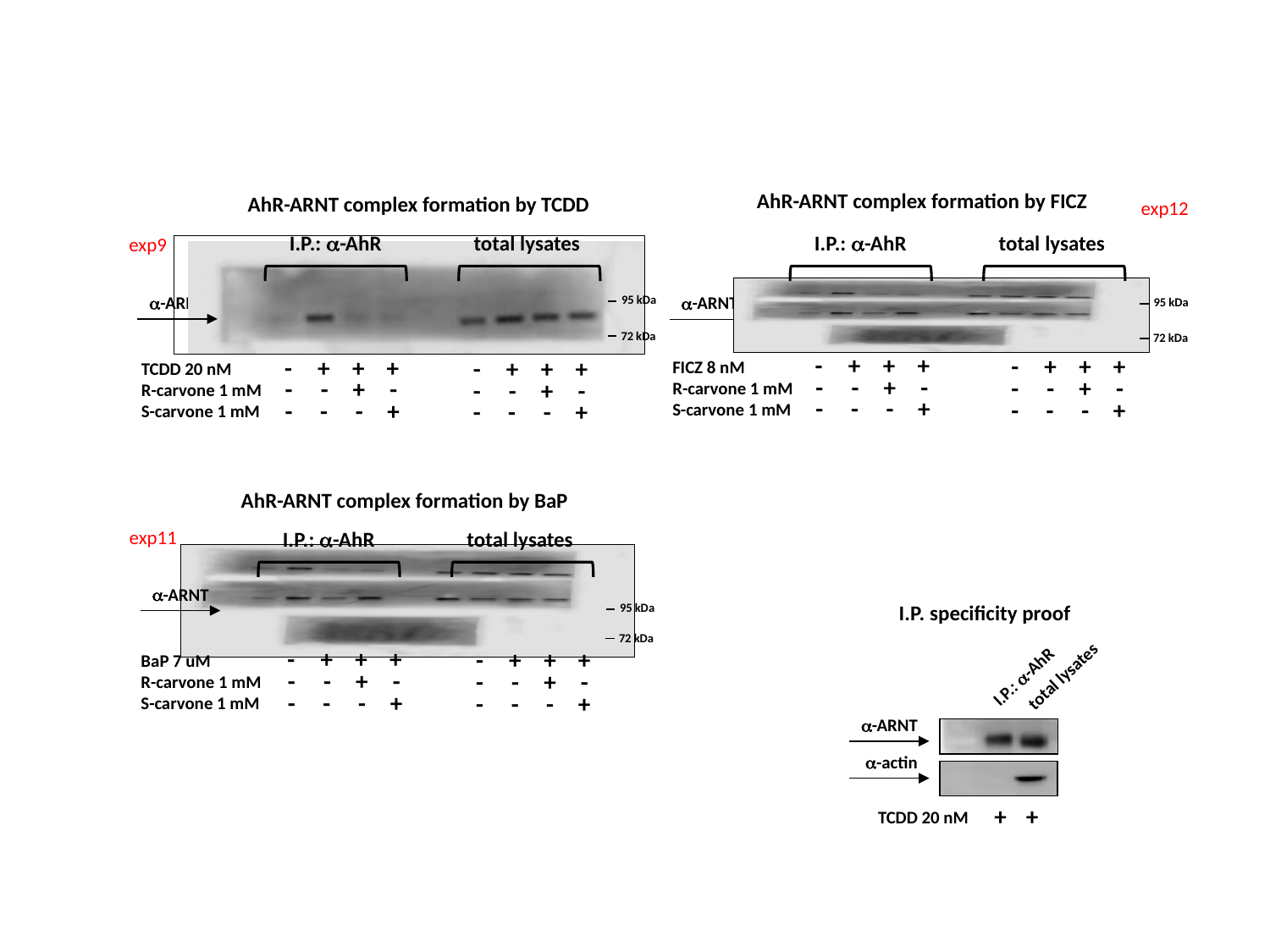

AhR-ARNT complex formation by FICZ
AhR-ARNT complex formation by TCDD
exp12
total lysates
total lysates
I.P.: a-AhR
I.P.: a-AhR
exp9
a-ARNT
95 kDa
a-ARNT
95 kDa
72 kDa
72 kDa
-
+
+
+
-
+
+
+
-
+
+
+
-
+
+
+
FICZ 8 nM
R-carvone 1 mM
S-carvone 1 mM
TCDD 20 nM
R-carvone 1 mM
S-carvone 1 mM
-
-
+
-
-
-
+
-
-
-
+
-
-
-
+
-
-
-
-
+
-
-
-
+
-
-
-
+
-
-
-
+
AhR-ARNT complex formation by BaP
exp11
total lysates
I.P.: a-AhR
a-ARNT
95 kDa
I.P. specificity proof
total lysates
I.P.: a-AhR
a-ARNT
a-actin
+
+
TCDD 20 nM
72 kDa
-
+
+
+
-
+
+
+
BaP 7 uM
R-carvone 1 mM
S-carvone 1 mM
-
-
+
-
-
-
+
-
-
-
-
+
-
-
-
+
