# Supplementary material for: Monoterpenoid aryl hydrocarbon receptor allosteric antagonists protect against ultraviolet skin damage in female mice
Source: Nat Commun. 2023 May 11;14:2728. doi: 10.1038/s41467-023-38478-6 (PMC10174618; doi:10.1038/s41467-023-38478-6)
Supplement: Supplementary file 4 — source data [file 41467_2023_38478_MOESM4_ESM.zip › DATA - ONDROVA new/Figure 4/Figure 4E/220414_AHR_ingel_digest_sample_K01_CHCA_8000shots_02/0_P19/1/1SRef/pdata/1/MSToolData.HTM]

Concise Summary Report (../data/20220414/F028523.dat)


# Mascot Search Results

```
User            : 
Email           : 
Search title    : 
Database        : SwissProt 2019_07 (560537 sequences; 201466755 residues)
Taxonomy        : Mammalia (mammals) (67185 sequences)
Timestamp       : 14 Apr 2022 at 10:35:37 GMT
Top Score       : 110 for AHR_HUMAN, Aryl hydrocarbon receptor OS=Homo sapiens OX=9606 GN=AHR PE=1 SV=2
```

### Mascot Score Histogram

Protein score is -10\*Log(P), where P is the probability that the observed match is a random event.  
Protein scores greater than 61 are significant (p<0.05).  

### Concise Protein Summary Report

|  |  |  |
| --- | --- | --- |
|  | Protein Summary Concise Protein Summary Export Search Results | Help |
|  | Significance threshold p< | Max. number of hits |


  

|  |  |
| --- | --- |
| **1.** | AHR\_HUMAN    **Mass:** 97113    **Score:** **110**  **Expect:** 6.7e-007  **Matches:** 19 |
|  | Aryl hydrocarbon receptor OS=Homo sapiens OX=9606 GN=AHR PE=1 SV=2 |

|  |  |
| --- | --- |
|  | APLD1\_RAT    **Mass:** 27211    **Score:** 43     **Expect:** 3.6  **Matches:** 6 |
|  | Apolipoprotein L domain-containing protein 1 OS=Rattus norvegicus OX=10116 GN=Apold1 PE=1 SV=1 |

|  |  |
| --- | --- |
|  | PB1\_HUMAN    **Mass:** 194080   **Score:** 41     **Expect:** 4.9  **Matches:** 12 |
|  | Protein polybromo-1 OS=Homo sapiens OX=9606 GN=PBRM1 PE=1 SV=1 |

|  |  |
| --- | --- |
|  | IMA1\_HUMAN    **Mass:** 58168    **Score:** 40     **Expect:** 6.6  **Matches:** 7 |
|  | Importin subunit alpha-1 OS=Homo sapiens OX=9606 GN=KPNA2 PE=1 SV=1 |

|  |  |
| --- | --- |
|  | R113A\_BOVIN    **Mass:** 39509    **Score:** 39     **Expect:** 7.9  **Matches:** 8 |
|  | E3 ubiquitin-protein ligase RNF113A OS=Bos taurus OX=9913 GN=RNF113A PE=2 SV=1 |

|  |  |
| --- | --- |
|  | R113A\_HUMAN    **Mass:** 39504    **Score:** 36     **Expect:** 15  **Matches:** 7 |
|  | E3 ubiquitin-protein ligase RNF113A OS=Homo sapiens OX=9606 GN=RNF113A PE=1 SV=1 |

|  |  |
| --- | --- |
|  | CCD63\_RAT    **Mass:** 66181    **Score:** 36     **Expect:** 18  **Matches:** 11 |
|  | Coiled-coil domain-containing protein 63 OS=Rattus norvegicus OX=10116 GN=Ccdc63 PE=2 SV=1 |

|  |  |
| --- | --- |
|  | ZNF92\_HUMAN    **Mass:** 70381    **Score:** 36     **Expect:** 19  **Matches:** 9 |
|  | Zinc finger protein 92 OS=Homo sapiens OX=9606 GN=ZNF92 PE=2 SV=2 |

|  |  |
| --- | --- |
|  | LSMD1\_MOUSE    **Mass:** 13707    **Score:** 35     **Expect:** 19  **Matches:** 5 |
|  | N-alpha-acetyltransferase 38, NatC auxiliary subunit OS=Mus musculus OX=10090 GN=Naa38 PE=1 SV=1 |

|  |  |
| --- | --- |
|  | LSMD1\_HUMAN    **Mass:** 13791    **Score:** 35     **Expect:** 20  **Matches:** 5 |
|  | N-alpha-acetyltransferase 38, NatC auxiliary subunit OS=Homo sapiens OX=9606 GN=NAA38 PE=1 SV=1 |

|  |  |
| --- | --- |
|  | ILKAP\_RAT    **Mass:** 43288    **Score:** 34     **Expect:** 30  **Matches:** 6 |
|  | Integrin-linked kinase-associated serine/threonine phosphatase 2C OS=Rattus norvegicus OX=10116 GN=Ilkap PE=2 SV=1 |

|  |  |
| --- | --- |
|  | DDIT3\_HUMAN    **Mass:** 19163    **Score:** 33     **Expect:** 31  **Matches:** 4 |
|  | DNA damage-inducible transcript 3 protein OS=Homo sapiens OX=9606 GN=DDIT3 PE=1 SV=1 |

|  |  |
| --- | --- |
|  | Z280C\_HUMAN    **Mass:** 84867    **Score:** 33     **Expect:** 34  **Matches:** 9 |
|  | Zinc finger protein 280C OS=Homo sapiens OX=9606 GN=ZNF280C PE=1 SV=1 |

|  |  |
| --- | --- |
|  | ILKAP\_MOUSE    **Mass:** 43318    **Score:** 33     **Expect:** 35  **Matches:** 6 |
|  | Integrin-linked kinase-associated serine/threonine phosphatase 2C OS=Mus musculus OX=10090 GN=Ilkap PE=1 SV=1 |

|  |  |
| --- | --- |
|  | IL5\_HORSE    **Mass:** 15242    **Score:** 33     **Expect:** 36  **Matches:** 4 |
|  | Interleukin-5 OS=Equus caballus OX=9796 GN=IL5 PE=2 SV=1 |

|  |  |
| --- | --- |
|  | SDC2\_MOUSE    **Mass:** 22174    **Score:** 32     **Expect:** 41  **Matches:** 4 |
|  | Syndecan-2 OS=Mus musculus OX=10090 GN=Sdc2 PE=1 SV=1 |

|  |  |
| --- | --- |
|  | C1D\_CRIGR    **Mass:** 15935    **Score:** 32     **Expect:** 45  **Matches:** 5 |
|  | Nuclear nucleic acid-binding protein C1D OS=Cricetulus griseus OX=10029 GN=C1D PE=2 SV=1 |

|  |  |
| --- | --- |
|  | RM09\_PAPAN    **Mass:** 30629    **Score:** 32     **Expect:** 45  **Matches:** 6 |
|  | 39S ribosomal protein L9, mitochondrial OS=Papio anubis OX=9555 GN=MRPL9 PE=3 SV=1 |

|  |  |
| --- | --- |
|  | IMA1\_MOUSE    **Mass:** 58234    **Score:** 31     **Expect:** 55  **Matches:** 6 |
|  | Importin subunit alpha-1 OS=Mus musculus OX=10090 GN=Kpna2 PE=1 SV=2 |

|  |  |
| --- | --- |
|  | EXOC6\_MOUSE    **Mass:** 93872    **Score:** 31     **Expect:** 55  **Matches:** 8 |
|  | Exocyst complex component 6 OS=Mus musculus OX=10090 GN=Exoc6 PE=1 SV=2 |

|  |  |
| --- | --- |
|  | THEGL\_RAT    **Mass:** 51548    **Score:** 31     **Expect:** 59  **Matches:** 6 |
|  | Testicular haploid expressed gene protein-like OS=Rattus norvegicus OX=10116 GN=Thegl PE=2 SV=1 |

|  |  |
| --- | --- |
|  | MBNL2\_HUMAN    **Mass:** 41518    **Score:** 30     **Expect:** 63  **Matches:** 5 |
|  | Muscleblind-like protein 2 OS=Homo sapiens OX=9606 GN=MBNL2 PE=1 SV=2 |

|  |  |
| --- | --- |
|  | MBNL2\_PONAB    **Mass:** 41518    **Score:** 30     **Expect:** 63  **Matches:** 5 |
|  | Muscleblind-like protein 2 OS=Pongo abelii OX=9601 GN=MBNL2 PE=2 SV=2 |

|  |  |
| --- | --- |
|  | DDAH1\_HUMAN    **Mass:** 31444    **Score:** 30     **Expect:** 63  **Matches:** 5 |
|  | N(G),N(G)-dimethylarginine dimethylaminohydrolase 1 OS=Homo sapiens OX=9606 GN=DDAH1 PE=1 SV=3 |

|  |  |
| --- | --- |
|  | CHST7\_MOUSE    **Mass:** 55303    **Score:** 30     **Expect:** 69  **Matches:** 7 |
|  | Carbohydrate sulfotransferase 7 OS=Mus musculus OX=10090 GN=Chst7 PE=2 SV=1 |

|  |  |
| --- | --- |
|  | MMP21\_MOUSE    **Mass:** 65698    **Score:** 30     **Expect:** 70  **Matches:** 7 |
|  | Matrix metalloproteinase-21 OS=Mus musculus OX=10090 GN=Mmp21 PE=1 SV=1 |

|  |  |
| --- | --- |
|  | USH1G\_MOUSE    **Mass:** 51857    **Score:** 30     **Expect:** 75  **Matches:** 6 |
|  | Usher syndrome type-1G protein homolog OS=Mus musculus OX=10090 GN=Ush1g PE=1 SV=1 |

|  |  |
| --- | --- |
|  | CAZA2\_PLEMO    **Mass:** 33161    **Score:** 29     **Expect:** 77  **Matches:** 5 |
|  | F-actin-capping protein subunit alpha-2 OS=Plecturocebus moloch OX=9523 GN=CAPZA2 PE=3 SV=3 |

|  |  |
| --- | --- |
|  | CASK\_RABIT    **Mass:** 20387    **Score:** 29     **Expect:** 77  **Matches:** 4 |
|  | Kappa-casein OS=Oryctolagus cuniculus OX=9986 GN=CSN3 PE=2 SV=1 |

|  |  |
| --- | --- |
|  | HRH2\_GORGO    **Mass:** 40813    **Score:** 29     **Expect:** 81  **Matches:** 5 |
|  | Histamine H2 receptor OS=Gorilla gorilla gorilla OX=9595 GN=HRH2 PE=3 SV=1 |

|  |  |
| --- | --- |
|  | HRH2\_HUMAN    **Mass:** 40813    **Score:** 29     **Expect:** 81  **Matches:** 5 |
|  | Histamine H2 receptor OS=Homo sapiens OX=9606 GN=HRH2 PE=2 SV=1 |

---

|  |  |
| --- | --- |
| **2.** | CD4\_RAT    **Mass:** 52089    **Score:** 40     **Expect:** 6.1  **Matches:** 9 |
|  | T-cell surface glycoprotein CD4 OS=Rattus norvegicus OX=10116 GN=Cd4 PE=1 SV=1 |

---

|  |  |
| --- | --- |
| **3.** | URIC\_PIG    **Mass:** 35214    **Score:** 39     **Expect:** 7.7  **Matches:** 6 |
|  | Uricase OS=Sus scrofa OX=9823 GN=UOX PE=2 SV=3 |

---

|  |  |
| --- | --- |
| **4.** | PRR11\_HUMAN    **Mass:** 40573    **Score:** 38     **Expect:** 10  **Matches:** 8 |
|  | Proline-rich protein 11 OS=Homo sapiens OX=9606 GN=PRR11 PE=1 SV=1 |

|  |  |
| --- | --- |
|  | ACYP2\_CAVPO    **Mass:** 11058    **Score:** 30     **Expect:** 75  **Matches:** 4 |
|  | Acylphosphatase-2 OS=Cavia porcellus OX=10141 GN=ACYP2 PE=1 SV=2 |

---

|  |  |
| --- | --- |
| **5.** | FMO5\_RABIT    **Mass:** 60320    **Score:** 37     **Expect:** 13  **Matches:** 7 |
|  | Dimethylaniline monooxygenase [N-oxide-forming] 5 OS=Oryctolagus cuniculus OX=9986 GN=FMO5 PE=2 SV=2 |

---

|  |  |
| --- | --- |
| **6.** | JDP2\_MOUSE    **Mass:** 18777    **Score:** 37     **Expect:** 13  **Matches:** 7 |
|  | Jun dimerization protein 2 OS=Mus musculus OX=10090 GN=Jdp2 PE=1 SV=2 |

|  |  |
| --- | --- |
|  | JDP2\_RAT    **Mass:** 18777    **Score:** 37     **Expect:** 13  **Matches:** 7 |
|  | Jun dimerization protein 2 OS=Rattus norvegicus OX=10116 GN=Jdp2 PE=1 SV=1 |

|  |  |
| --- | --- |
|  | JDP2\_HUMAN    **Mass:** 18806    **Score:** 36     **Expect:** 15  **Matches:** 7 |
|  | Jun dimerization protein 2 OS=Homo sapiens OX=9606 GN=JDP2 PE=1 SV=1 |

---

|  |  |
| --- | --- |
| **7.** | DHX30\_BOVIN    **Mass:** 136945   **Score:** 35     **Expect:** 20  **Matches:** 10 |
|  | ATP-dependent RNA helicase DHX30 OS=Bos taurus OX=9913 GN=DHX30 PE=2 SV=1 |

---

|  |  |
| --- | --- |
| **8.** | SFI1\_CALJA    **Mass:** 148014   **Score:** 35     **Expect:** 22  **Matches:** 11 |
|  | Protein SFI1 homolog OS=Callithrix jacchus OX=9483 GN=SFI1 PE=3 SV=1 |

---

|  |  |
| --- | --- |
| **9.** | FMO5\_MOUSE    **Mass:** 60532    **Score:** 34     **Expect:** 28  **Matches:** 7 |
|  | Dimethylaniline monooxygenase [N-oxide-forming] 5 OS=Mus musculus OX=10090 GN=Fmo5 PE=1 SV=4 |

---

|  |  |
| --- | --- |
| **10.** | RBL2\_HUMAN    **Mass:** 129711   **Score:** 33     **Expect:** 37  **Matches:** 10 |
|  | Retinoblastoma-like protein 2 OS=Homo sapiens OX=9606 GN=RBL2 PE=1 SV=3 |

---

|  |  |
| --- | --- |
| **11.** | PKHG6\_MOUSE    **Mass:** 89435    **Score:** 32     **Expect:** 48  **Matches:** 9 |
|  | Pleckstrin homology domain-containing family G member 6 OS=Mus musculus OX=10090 GN=Plekhg6 PE=2 SV=2 |

---

|  |  |
| --- | --- |
| **12.** | PPM1K\_BOVIN    **Mass:** 41524    **Score:** 30     **Expect:** 64  **Matches:** 5 |
|  | Protein phosphatase 1K, mitochondrial OS=Bos taurus OX=9913 GN=PPM1K PE=2 SV=1 |

---

|  |  |
| --- | --- |
| **13.** | WHAMM\_MOUSE    **Mass:** 89968    **Score:** 30     **Expect:** 67  **Matches:** 9 |
|  | WASP homolog-associated protein with actin, membranes and microtubules OS=Mus musculus OX=10090 GN=Whamm PE=1 SV=2 |

---

|  |  |
| --- | --- |
| **14.** | TFE3\_MOUSE    **Mass:** 61556    **Score:** 30     **Expect:** 67  **Matches:** 6 |
|  | Transcription factor E3 OS=Mus musculus OX=10090 GN=Tfe3 PE=1 SV=2 |

---

|  |  |
| --- | --- |
| **15.** | PRS35\_RAT    **Mass:** 45924    **Score:** 30     **Expect:** 70  **Matches:** 6 |
|  | Inactive serine protease 35 OS=Rattus norvegicus OX=10116 GN=Prss35 PE=2 SV=1 |

---

|  |  |
| --- | --- |
| **16.** | K2C3\_HUMAN    **Mass:** 64549    **Score:** 30     **Expect:** 74  **Matches:** 7 |
|  | Keratin, type II cytoskeletal 3 OS=Homo sapiens OX=9606 GN=KRT3 PE=1 SV=3 |

---

|  |  |
| --- | --- |
| **17.** | MTEF2\_MOUSE    **Mass:** 44052    **Score:** 29     **Expect:** 79  **Matches:** 6 |
|  | Transcription termination factor 2, mitochondrial OS=Mus musculus OX=10090 GN=Mterf2 PE=1 SV=1 |

---

### Search Parameters

```
Type of search         : Peptide Mass Fingerprint
Enzyme                 : Trypsin
Fixed modifications    : Carbamidomethyl (C)
Variable modifications : Deamidated (NQ),Oxidation (M)
Mass values            : Monoisotopic
Protein Mass           : Unrestricted
Peptide Mass Tolerance : ± 80 ppm
Peptide Charge State   : 1+
Max Missed Cleavages   : 2
Number of queries      : 22
```

|  |
| --- |
| **Mascot:** http://www.matrixscience.com/ |
