# Supplementary material for: Monoterpenoid aryl hydrocarbon receptor allosteric antagonists protect against ultraviolet skin damage in female mice
Source: Nat Commun. 2023 May 11;14:2728. doi: 10.1038/s41467-023-38478-6 (PMC10174618; doi:10.1038/s41467-023-38478-6)
Supplement: Supplementary file 4 — source data [file 41467_2023_38478_MOESM4_ESM.zip › DATA - ONDROVA new/Figure 4/Figure 4E/220414_AHR_ingel_digest_sample_K01_CHCA_8000shots_02/0_P19/1/1SRef/pdata/1/MSToolMascotResults]

 Mascot search status page


```
MIME-Version: 1.0 (Generated by Mascot version 1.0)
Content-Type: multipart/mixed; boundary=gc0p4Jq0M2Yt08jU534c0p

--gc0p4Jq0M2Yt08jU534c0p
Content-Type: application/x-Mascot; name="parameters"

LICENSE=Palacky University in Olomouc  (JL68-KGZ2-FUR6-AUVA-H55D)
MP=
NM=TRUE
COM=
IATOL=
IA2TOL=
IASTOL=
IBTOL=
IB2TOL=
IBSTOL=
IYTOL=
IY2TOL=
IYSTOL=
SEG=
SEGT=
SEGTU=
LTOL=
TOL=80
TOLU=ppm
ITH=
ITOL=
ITOLU=
PFA=2
DB=SwissProt
MODS=Carbamidomethyl (C)
MASS=Monoisotopic
CLE=Trypsin
FILE=
PEAK=AUTO
QUE=
TWO=
SEARCH=PMF
USERNAME=
USEREMAIL=
CHARGE=1+
INTERMEDIATE=
REPORT=20
OVERVIEW=ON
FORMAT=Mascot generic
FORMVER=1.01
FRAG=
IT_MODS=Deamidated (NQ),Oxidation (M)
USER00=PEP_INT(578.308108:3563.000000,587.396523:3755.000000,650.048860:8120.000000,676.341244:1373.000000,688.450884:1684.000000,745.403923:2716.000000,755.337537:6439.000000,837.464949:12252.000000,860.434240:2878.000000,920.363419:1137.000000,926.473176:5232.000000,943.587130:1375.000000,986.521862:992.000000,1131.542589:1372.000000,1479.731639:776.000000,1554.851373:3769.000000,1583.774093:898.000000,1901.924999:1747.000000,1911.124459:7071.000000,1917.926783:1966.000000,2150.265692:1271.000000,2187.103818:738.000000,)
USER01=
USER02=
USER03=
USER04=
USER05=
USER06=
USER07=
USER08=
USER09=
USER10=
USER11=
USER12=
PRECURSOR=
TAXONOMY=. . . . . . . . . . . . Mammalia (mammals)
ACCESSION=
REPTYPE=concise
SUBCLUSTER=
ICAT=
INSTRUMENT=
ERRORTOLERANT=
FRAMES=
CUTOUT=
USERID=0
QUANTITATION=
DECOY=
PEP_ISOTOPE_ERROR=
MULTI_SITE_MODS=
RULES=1,2,5,6,8,9,13,14
INTERNALS=0.0,700.0
--gc0p4Jq0M2Yt08jU534c0p
Content-Type: application/x-Mascot; name="masses"

A=71.037114
B=114.534940
C=160.030649
D=115.026943
E=129.042593
F=147.068414
G=57.021464
H=137.058912
I=113.084064
J=0.000000
K=128.094963
L=113.084064
M=131.040485
N=114.042927
O=0.000000
P=97.052764
Q=128.058578
R=156.101111
S=87.032028
T=101.047679
U=150.953633
V=99.068414
W=186.079313
X=111.000000
Y=163.063329
Z=128.550590
Hydrogen=1.007825
Carbon=12.000000
Nitrogen=14.003074
Oxygen=15.994915
Electron=0.000549
C_term=17.002740
N_term=1.007825
delta1=0.984016,Deamidated (NQ)
NeutralLoss1=0.000000
delta2=15.994915,Oxidation (M)
NeutralLoss2=0.000000
NeutralLoss2_master=63.998285
FixedMod1=57.021464,Carbamidomethyl (C)
FixedModResidues1=C
--gc0p4Jq0M2Yt08jU534c0p
Content-Type: application/x-Mascot; name="unimod"

xml version="1.0" encoding="UTF-8" ?

  
    
    
    
    
    
    
    
    
    
    
    
    
    
    
    
    
    
    
    
    
    
    
    
    
    
    
    
    
    
    
    
    
    
    
    
    
  
  
    
      
      
      
      
      
      
      
      
      
      
      
        
        
        
        
      
      Carboxyamidomethylation
      
        12422359
        PubMed PMID
        
      
      
        Boja, E. S., Fales, H. M., Anal. Chem. 73 3576-82 (2001)
        Journal
        
      
      
        11510821
        PubMed PMID
        
      
      
        Creasy, D. M., Cottrell, J. S., Proteomics 2 1426-34 (2002)
        Journal
        
      
    
    
      
      
        Protein which is post-translationally modified by the de-imination of one or more arginine residues; Peptidylarginine deiminase (PAD) converts protein bound to citrulline
      
      
        Convertion of glycosylated asparagine residues upon deglycosylation with PNGase F in H2O
      
      
      
        
        
        
      
      phenyllactyl from N-term Phe
      Citrullination
      
        FLAC
        FindMod
        
      
      
        AA0128
        RESID
        
      
      
        CITR
        FindMod
        
      
      
        IonSource tutorial
        Misc. URL
        http://www.ionsource.com/Card/Deamidation/deamidation.htm
      
      
        AA0214
        RESID
        
      
      
        6838602
        PubMed PMID
        
      
      
        15700232
        PubMed PMID
        
      
      
        DEAM
        FindMod
        
      
    
    
      
      
      
        Cysteine sulfenic acid
      
      
        
        
          
          
          
          
        
      
      
      
      
      
        Proline oxidation to glutamic semialdehyde
      
      
      
      
      
        Hydroxyglycine derivative in amidation pathway
      
      
      
        
      
      
        AA0235
        RESID
        
      
      
        AA0027
        RESID
        
      
      
        11461766
        PubMed PMID
        
      
      
        AA0028
        RESID
        
      
      
        9004526
        PubMed PMID
        
      
      
        AA0030
        RESID
        
      
      
        AA0029
        RESID
        
      
      
        Lagerwerf FM, van de Weert M, Heerma W, Haverkamp J, Rapid Commun Mass Spectrom. 1996;10(15):1905-10
        Journal
        
      
      
        AA0215
        RESID
        
      
      
        AA0205
        RESID
        
      
      
        15569593
        PubMed PMID
        
      
      
        Berlett, Barbara S.; Stadtman, Earl R. Journal of Biological Chemistry (1997), 272(33), 20313-20316.
        Journal
        
      
      
        AA0026
        RESID
        
      
      
        14661084
        PubMed PMID
        
      
      
        CSEA
        FindMod
        
      
      
        DOPA
        FindMod
        
      
      
        11120890
        PubMed PMID
        
      
      
        11212008
        PubMed PMID
        
      
      
        AA0322
        RESID
        
      
      
        HYDR
        FindMod
        
      
      
        AA0146
        RESID
        
      
      
        14661085
        PubMed PMID
        
      
      
        12781462
        PubMed PMID
        
      
      
        2057999
        PubMed PMID
        
      
    
  
  
    
    
      
      
      
      
    
    
      
      
      
      
    
    
      
      
      
      
    
    
      
      
      
      
    
    
      
      
      
      
      
    
    
      
      
      
      
    
    
      
      
      
      
    
    
      
      
      
      
    
    
      
      
      
      
    
    
      
      
      
      
    
    
      
      
      
      
    
    
      
      
      
      
    
    
      
      
      
      
      
    
    
      
      
      
      
    
    
      
      
      
      
    
    
      
      
      
      
    
    
      
      
      
      
    
    
      
      
      
      
    
    
      
      
      
      
    
    
      
      
      
      
    
    
      
    
    
      
      
    
    
      
      
      
      
      
    
  


--gc0p4Jq0M2Yt08jU534c0p
Content-Type: application/x-Mascot; name="enzyme"

Title:Trypsin
Cleavage:KR
Restrict:P
Cterm
*
--gc0p4Jq0M2Yt08jU534c0p
Content-Type: application/x-Mascot; name="taxonomy"

Title:. . . . . . . . . . . . Mammalia (mammals)
Include: 40674
Exclude:
*
--gc0p4Jq0M2Yt08jU534c0p
Content-Type: application/x-Mascot; name="header"

sequences=560537
sequences_after_tax=67185
residues=201466755
distribution=49813,12,137,633,868,1876,1841,1681,1512,1441,1240,1060,1072,819,761,602,466,400,256,167,114,112,58,48,43,37,23,30,7,18,8,3,5,3,1,5,2,3,1,2,2,1,1,0,0,0,0,0,0,0,0,0,0,0,0,0,0,0,0,0,0,0,0,0,0,0,0,0,0,0,0,0,0,0,0,0,0,0,0,0,0,0,0,0,0,0,0,0,0,0,0,0,0,0,0,0,0,0,0,0,0,0,0,0,0,0,0,0,0,0,1
exec_time=7
date=1649932537
time=12:35:37
queries=22
min_peaks_for_homology=6
pmf_num_queries_used=22
pmf_queries_used=1,2,3,4,5,6,7,8,9,10,11,12,13,14,15,16,17,18,19,20,21,22
pmf_hits_preserved=100
max_hits=50
version=2.4.0
fastafile=C:/inetpub/mascot/sequence/SwissProt/current/SwissProt_2019_07.fasta
release=SwissProt_2019_07.fasta
--gc0p4Jq0M2Yt08jU534c0p
Content-Type: application/x-Mascot; name="summary"

qmass1=577.300832
qexp1=578.308108,1+
qintensity1=3563.0000
qmatch1=0
qplughole1=0.000000
qmass2=586.389247
qexp2=587.396523,1+
qintensity2=3755.0000
qmatch2=0
qplughole2=0.000000
qmass3=649.041584
qexp3=650.048860,1+
qintensity3=8120.0000
qmatch3=0
qplughole3=0.000000
qmass4=675.333968
qexp4=676.341244,1+
qintensity4=1373.0000
qmatch4=0
qplughole4=0.000000
qmass5=687.443608
qexp5=688.450884,1+
qintensity5=1684.0000
qmatch5=0
qplughole5=0.000000
qmass6=744.396647
qexp6=745.403923,1+
qintensity6=2716.0000
qmatch6=0
qplughole6=0.000000
qmass7=754.330261
qexp7=755.337537,1+
qintensity7=6439.0000
qmatch7=0
qplughole7=0.000000
qmass8=836.457673
qexp8=837.464949,1+
qintensity8=12252.0000
qmatch8=0
qplughole8=0.000000
qmass9=859.426964
qexp9=860.434240,1+
qintensity9=2878.0000
qmatch9=0
qplughole9=0.000000
qmass10=919.356143
qexp10=920.363419,1+
qintensity10=1137.0000
qmatch10=0
qplughole10=0.000000
qmass11=925.465900
qexp11=926.473176,1+
qintensity11=5232.0000
qmatch11=0
qplughole11=0.000000
qmass12=942.579854
qexp12=943.587130,1+
qintensity12=1375.0000
qmatch12=0
qplughole12=0.000000
qmass13=985.514586
qexp13=986.521862,1+
qintensity13=992.0000
qmatch13=0
qplughole13=0.000000
qmass14=1130.535313
qexp14=1131.542589,1+
qintensity14=1372.0000
qmatch14=0
qplughole14=0.000000
qmass15=1478.724363
qexp15=1479.731639,1+
qintensity15=776.0000
qmatch15=0
qplughole15=0.000000
qmass16=1553.844097
qexp16=1554.851373,1+
qintensity16=3769.0000
qmatch16=0
qplughole16=0.000000
qmass17=1582.766817
qexp17=1583.774093,1+
qintensity17=898.0000
qmatch17=0
qplughole17=0.000000
qmass18=1900.917723
qexp18=1901.924999,1+
qintensity18=1747.0000
qmatch18=0
qplughole18=0.000000
qmass19=1910.117183
qexp19=1911.124459,1+
qintensity19=7071.0000
qmatch19=0
qplughole19=0.000000
qmass20=1916.919507
qexp20=1917.926783,1+
qintensity20=1966.0000
qmatch20=0
qplughole20=0.000000
qmass21=2149.258416
qexp21=2150.265692,1+
qintensity21=1271.0000
qmatch21=0
qplughole21=0.000000
qmass22=2186.096542
qexp22=2187.103818,1+
qintensity22=738.0000
qmatch22=0
qplughole22=0.000000
num_hits=50
h1=AHR_HUMAN,1.10e+002,0.86,97112.63
h1_text=Aryl hydrocarbon receptor OS=Homo sapiens OX=9606 GN=AHR PE=1 SV=2
h1_q1=0,577.297226,0.003606,103,107,0.00,AANFR,0,0000000,0.00,1,0000000000000000000,0,0,0.000000
h1_q1_terms=R,E
h1_q2=0,586.380234,0.009013,67,71,0.00,LSVLR,0,0000000,0.00,1,0000000000000000000,0,0,0.000000
h1_q2_terms=K,L
h1_q3=-1
h1_q4=0,675.318756,0.015212,89,94,0.00,SSPTER,0,00000000,0.00,1,0000000000000000000,0,0,0.000000
h1_q4_terms=K,N
h1_q5=-1
h1_q6=0,744.391861,0.004786,245,250,0.00,YLHGQK,0,00000000,0.00,1,0000000000000000000,0,0,0.000000
h1_q6_terms=K,K
h1_q7=0,754.325455,0.004806,219,223,0.00,CFICR,0,0000000,0.00,1,0000000000000000000,0,0,0.000000
h1_q7_terms=R,L
h1_q8=0,836.475586,-0.017913,72,78,0.00,LSVSYLR,0,000000000,0.00,1,0000000000000000000,0,0,0.000000
h1_q8_terms=R,A
h1_q9=0,859.439926,-0.012962,43,49,0.00,LNTELDR,0,000000000,0.00,1,0000000000000000000,0,0,0.000000
h1_q9_terms=R,L
h1_q10=0,919.356628,-0.000485,95,102,0.00,NGGQDNCR,0,0000000000,0.00,1,0000000000000000000,0,0,0.000000
h1_q10_terms=R,A
h1_q11=0,925.490921,-0.025021,81,88,0.00,SFFDVALK,0,0000000000,0.00,1,0000000000000000000,0,0,0.000000
h1_q11_terms=K,S
h1_q12=1,942.586197,-0.006343,64,71,0.00,LDKLSVLR,0,0000000000,0.00,1,0000000000000000000,0,0,0.000000
h1_q12_terms=K,L
h1_q13=1,985.570877,-0.056291,243,250,0.00,LKYLHGQK,0,0000000000,0.00,1,0000000000000000000,0,0,0.000000
h1_q13_terms=K,K
h1_q14=1,1130.567978,-0.032665,41,49,0.00,DRLNTELDR,0,00000000000,0.00,1,0000000000000000000,0,0,0.000000
h1_q14_terms=R,L
h1_q15=1,1478.643295,0.081068,95,107,0.00,NGGQDNCRAANFR,0,000000000000000,0.00,1,0000000000000000000,0,0,0.000000
h1_q15_terms=R,E
h1_q16=0,1553.881714,-0.037617,50,63,0.00,LASLLPFPQDVINK,0,0000000000000000,0.00,2,0000000000000000000,0,0,0.000000
h1_q16_terms=R,L
h1_q17=1,1582.799118,-0.032301,81,94,0.00,SFFDVALKSSPTER,0,0000000000000000,0.00,1,0000000000000000000,0,0,0.000000
h1_q17_terms=K,N
h1_q18=0,1900.881149,0.036574,226,242,0.00,CLLDNSSGFLAMNFQGK,0,0000000000000000000,0.00,1,0000000000000000000,0,0,0.000000
h1_q18_terms=R,L
h1_q19=1,1910.087677,0.029506,50,66,0.00,LASLLPFPQDVINKLDK,0,0000000000000000000,0.00,1,0000000000000000000,0,0,0.000000
h1_q19_terms=R,L
h1_q20=0,1916.876068,0.043439,226,242,0.00,CLLDNSSGFLAMNFQGK,0,0000000000002000000,0.00,1,0000000000000000000,0,0,0.000000
h1_q20_terms=R,L
h1_q21=-1
h1_q22=1,2186.061234,0.035308,224,242,0.00,LRCLLDNSSGFLAMNFQGK,0,000000000000002000000,0.00,1,0000000000000000000,0,0,0.000000
h1_q22_terms=R,L
h2=APLD1_RAT,4.27e+001,0.27,27211.21
h2_text=Apolipoprotein L domain-containing protein 1 OS=Rattus norvegicus OX=10116 GN=Apold1 PE=1 SV=1
h2_q1=-1
h2_q2=1,586.380219,0.009028,195,199,0.00,AKIQK,0,0000000,0.00,1,0000000000000000000,0,0,0.000000
h2_q2_terms=K,L
h2_q3=-1
h2_q4=-1
h2_q5=-1
h2_q6=0,744.438141,-0.041494,188,194,0.00,VSQAVLK,0,000100000,0.00,1,0000000000000000000,0,0,0.000000
h2_q6_terms=K,A
h2_q7=-1
h2_q8=-1
h2_q9=-1
h2_q10=-1
h2_q11=0,925.480362,-0.014462,29,35,0.00,LHCQVLR,0,000010000,0.00,1,0000000000000000000,0,0,0.000000
h2_q11_terms=R,L
h2_q12=1,942.586197,-0.006343,188,196,0.00,VSQAVLKAK,0,00000000000,0.00,1,0000000000000000000,0,0,0.000000
h2_q12_terms=K,I
h2_q13=-1
h2_q14=1,1130.567978,-0.032665,146,155,0.00,GDRQLLQSGR,0,000010010000,0.00,1,0000000000000000000,0,0,0.000000
h2_q14_terms=R,D
h2_q15=-1
h2_q16=-1
h2_q17=-1
h2_q18=-1
h2_q19=-1
h2_q20=-1
h2_q21=-1
h2_q22=0,2186.050659,0.045883,156,174,0.00,DASMALYNSVYFIVFFGSR,0,000000000000000000000,0.00,1,0000000000000000000,0,0,0.000000
h2_q22_terms=R,G
h3=PB1_HUMAN,4.14e+001,0.55,194079.70
h3_text=Protein polybromo-1 OS=Homo sapiens OX=9606 GN=PBRM1 PE=1 SV=1
h3_q1=1,577.300598,0.000234,521,524,0.00,QRMK,0,000200,0.00,2,0000000000000000000,0,0,0.000000
h3_q1_terms=K,I
h3_q2=-1
h3_q3=-1
h3_q4=0,675.343887,-0.009919,1419,1424,0.00,NLETAK,0,01000000,0.00,1,0000000000000000000,0,0,0.000000
h3_q4_terms=R,K
h3_q5=-1
h3_q6=0,744.351440,0.045207,828,833,0.00,NVENNR,0,00000000,0.00,1,0000000000000000000,0,0,0.000000
h3_q6_terms=K,Y
h3_q7=0,754.313324,0.016937,1261,1266,0.00,YNESDK,0,00000000,0.00,1,0000000000000000000,0,0,0.000000
h3_q7_terms=R,Q
h3_q8=-1
h3_q9=0,859.403534,0.023430,1435,1441,0.00,VAEQQER,0,000010000,0.00,2,0000000000000000000,0,0,0.000000
h3_q9_terms=K,E
h3_q10=-1
h3_q11=-1
h3_q12=1,942.574936,0.004918,1308,1315,0.00,KIQLLEAK,0,0001000000,0.00,1,0000000000000000000,0,0,0.000000
h3_q12_terms=K,F
h3_q13=2,985.541702,-0.027116,333,341,0.00,NKRAVQGGR,0,01000000000,0.00,1,0000000000000000000,0,0,0.000000
h3_q13_terms=R,L
h3_q14=1,1130.556717,-0.021404,1432,1441,0.00,AAKVAEQQER,0,000000011000,0.00,1,0000000000000000000,0,0,0.000000
h3_q14_terms=R,E
h3_q15=-1
h3_q16=1,1553.779770,0.064327,310,324,0.00,LTGPSHSKGSLGEER,0,00000000000000000,0.00,1,0000000000000000000,0,0,0.000000
h3_q16_terms=R,N
h3_q17=1,1582.762711,0.004106,258,271,0.00,NAKTYNEPGSQVFK,0,0100000000000000,0.00,1,0000000000000000000,0,0,0.000000
h3_q17_terms=K,D
h3_q18=-1
h3_q19=-1
h3_q20=1,1916.974442,-0.054935,1395,1411,0.00,AVIKAQHPDYSFGELSR,0,0000000000000000000,0.00,1,0000000000000000000,0,0,0.000000
h3_q20_terms=R,L
h3_q21=-1
h3_q22=2,2186.159119,-0.062577,1380,1398,0.00,KINMSGYILFSSEMRAVIK,0,000000000000000000000,0.00,1,0000000000000000000,0,0,0.000000
h3_q22_terms=R,A
h4=CD4_RAT,4.04e+001,0.41,52089.06
h4_text=T-cell surface glycoprotein CD4 OS=Rattus norvegicus OX=10116 GN=Cd4 PE=1 SV=1
h4_q1=-1
h4_q2=-1
h4_q3=-1
h4_q4=0,675.297607,0.036361,87,91,0.00,NAWER,0,0100000,0.00,1,0000000000000000000,0,0,0.000000
h4_q4_terms=K,G
h4_q5=0,687.416672,0.026936,384,389,0.00,IQVLSK,0,00100000,0.00,1,0000000000000000000,0,0,0.000000
h4_q5_terms=K,G
h4_q6=1,744.449356,-0.052709,436,441,0.00,RLLSEK,0,00000000,0.00,1,0000000000000000000,0,0,0.000000
h4_q6_terms=K,K
h4_q7=-1
h4_q8=-1
h4_q9=-1
h4_q10=-1
h4_q11=-1
h4_q12=-1
h4_q13=1,985.501480,0.013106,450,457,0.00,MQKSHNLI,0,0200000000,0.00,1,0000000000000000000,0,0,0.000000
h4_q13_terms=R,-
h4_q14=-1
h4_q15=2,1478.654068,0.070295,442,452,0.00,KTCQCSHRMQK,0,0000000002000,0.00,2,0000000000000000000,0,0,0.000000
h4_q15_terms=K,S
h4_q16=-1
h4_q17=2,1582.799759,-0.032942,423,435,0.00,HQQRQAARMSQIK,0,001100000000000,0.00,1,0000000000000000000,0,0,0.000000
h4_q17_terms=R,R
h4_q18=2,1900.834198,0.083525,443,457,0.00,TCQCSHRMQKSHNLI,0,00010000010000000,0.00,1,0000000000000000000,0,0,0.000000
h4_q18_terms=K,-
h4_q19=-1
h4_q20=2,1916.829117,0.090390,443,457,0.00,TCQCSHRMQKSHNLI,0,00010000210000000,0.00,1,0000000000000000000,0,0,0.000000
h4_q20_terms=K,-
h4_q21=2,2149.116730,0.141686,243,261,0.00,AEKAPSSQSWITFSLKNQK,0,000000000000000000000,0.00,1,0000000000000000000,0,0,0.000000
h4_q21_terms=K,V
h4_q22=-1
h5=IMA1_HUMAN,4.01e+001,0.32,58168.08
h5_text=Importin subunit alpha-1 OS=Homo sapiens OX=9606 GN=KPNA2 PE=1 SV=1
h5_q1=-1
h5_q2=0,586.369003,0.020244,198,202,0.00,DLVIK,0,0000000,0.00,1,0000000000000000000,0,0,0.000000
h5_q2_terms=R,Y
h5_q3=-1
h5_q4=0,675.343903,-0.009935,454,459,0.00,LGETEK,0,00000000,0.00,1,0000000000000000000,0,0,0.000000
h5_q4_terms=K,L
h5_q5=-1
h5_q6=1,744.449356,-0.052709,103,108,0.00,LLSREK,0,00000000,0.00,1,0000000000000000000,0,0,0.000000
h5_q6_terms=K,Q
h5_q7=-1
h5_q8=1,836.439209,0.018464,389,395,0.00,ADFKTQK,0,000000000,0.00,1,0000000000000000000,0,0,0.000000
h5_q8_terms=K,E
h5_q9=-1
h5_q10=-1
h5_q11=-1
h5_q12=2,942.539902,0.039952,14,20,0.00,LHRFKNK,0,000000100,0.00,1,0000000000000000000,0,0,0.000000
h5_q12_terms=R,G
h5_q13=-1
h5_q14=-1
h5_q15=-1
h5_q16=-1
h5_q17=-1
h5_q18=0,1900.923843,-0.006120,84,101,0.00,GINSSNVENQLQATQAAR,0,00010000000000000000,0.00,1,0000000000000000000,0,0,0.000000
h5_q18_terms=K,K
h5_q19=-1
h5_q20=-1
h5_q21=-1
h5_q22=1,2186.194641,-0.098099,239,258,0.00,NKNPAPPIDAVEQILPTLVR,0,0101000000000000000000,0.00,1,0000000000000000000,0,0,0.000000
h5_q22_terms=R,L
h6=URIC_PIG,3.94e+001,0.27,35214.18
h6_text=Uricase OS=Sus scrofa OX=9823 GN=UOX PE=2 SV=3
h6_q1=-1
h6_q2=-1
h6_q3=-1
h6_q4=0,675.362518,-0.028550,273,278,0.00,MGLINK,0,00000100,0.00,1,0000000000000000000,0,0,0.000000
h6_q4_terms=K,E
h6_q5=0,687.416672,0.026936,216,221,0.00,SIVLQK,0,00000100,0.00,1,0000000000000000000,0,0,0.000000
h6_q5_terms=R,F
h6_q6=-1
h6_q7=-1
h6_q8=-1
h6_q9=0,859.465073,-0.038109,73,80,0.00,NTVNVLAK,0,0100100000,0.00,1,0000000000000000000,0,0,0.000000
h6_q9_terms=K,F
h6_q10=-1
h6_q11=-1
h6_q12=-1
h6_q13=-1
h6_q14=-1
h6_q15=-1
h6_q16=1,1553.845352,-0.001255,162,175,0.00,VLKTTQSGFEGFIK,0,0000000000000000,0.00,1,0000000000000000000,0,0,0.000000
h6_q16_terms=K,D
h6_q17=-1
h6_q18=-1
h6_q19=2,1910.051315,0.065868,159,175,0.00,DLKVLKTTQSGFEGFIK,0,0000000000000000000,0.00,1,0000000000000000000,0,0,0.000000
h6_q19_terms=K,D
h6_q20=-1
h6_q21=-1
h6_q22=1,2186.140503,-0.043961,273,291,0.00,MGLINKEEVLLPLDNPYGR,0,020000000000000000000,0.00,1,0000000000000000000,0,0,0.000000
h6_q22_terms=K,I
h7=R113A_BOVIN,3.93e+001,0.36,39508.99
h7_text=E3 ubiquitin-protein ligase RNF113A OS=Bos taurus OX=9913 GN=RNF113A PE=2 SV=1
h7_q1=-1
h7_q2=-1
h7_q3=-1
h7_q4=-1
h7_q5=-1
h7_q6=-1
h7_q7=0,754.325455,0.004806,262,266,0.00,CFICR,0,0000000,0.00,1,0000000000000000000,0,0,0.000000
h7_q7_terms=K,Q
h7_q8=0,836.402802,0.054871,155,161,0.00,GINNYQK,0,000100000,0.00,1,0000000000000000000,0,0,0.000000
h7_q8_terms=R,F
h7_q9=-1
h7_q10=-1
h7_q11=0,925.440598,0.025302,228,234,0.00,HGWQIER,0,000010000,0.00,1,0000000000000000000,0,0,0.000000
h7_q11_terms=K,E
h7_q12=1,942.574936,0.004918,312,319,0.00,ELIAKLEK,0,0000000000,0.00,1,0000000000000000000,0,0,0.000000
h7_q12_terms=K,H
h7_q13=-1
h7_q14=1,1130.593109,-0.057796,137,145,0.00,SQKIQEELR,0,00100000000,0.00,1,0000000000000000000,0,0,0.000000
h7_q14_terms=R,G
h7_q15=1,1478.718765,0.005598,267,278,0.00,QTFQNPVVTKCR,0,01001000000000,0.00,1,0000000000000000000,0,0,0.000000
h7_q15_terms=R,H
h7_q16=-1
h7_q17=-1
h7_q18=2,1900.986877,-0.069154,152,166,0.00,IYRGINNYQKFMKPK,0,00000011000000000,0.00,1,0000000000000000000,0,0,0.000000
h7_q18_terms=K,D
h7_q19=-1
h7_q20=2,1916.981796,-0.062289,152,166,0.00,IYRGINNYQKFMKPK,0,00000011000020000,0.00,1,0000000000000000000,0,0,0.000000
h7_q20_terms=K,D
h7_q21=-1
h7_q22=-1
h8=PRR11_HUMAN,3.81e+001,0.36,40572.74
h8_text=Proline-rich protein 11 OS=Homo sapiens OX=9606 GN=PRR11 PE=1 SV=1
h8_q1=1,577.333618,-0.032786,4,7,0.00,FKQR,0,000000,0.00,1,0000000000000000000,0,0,0.000000
h8_q1_terms=K,R
h8_q2=2,586.416595,-0.027348,10,14,0.00,KLKAK,0,0000000,0.00,1,0000000000000000000,0,0,0.000000
h8_q2_terms=R,A
h8_q3=-1
h8_q4=-1
h8_q5=0,687.416687,0.026921,231,236,0.00,DLLTVK,0,00000000,0.00,1,0000000000000000000,0,0,0.000000
h8_q5_terms=K,L
h8_q6=1,744.412994,-0.016347,301,306,0.00,KVDVER,0,00000000,0.00,1,0000000000000000000,0,0,0.000000
h8_q6_terms=R,S
h8_q7=-1
h8_q8=-1
h8_q9=-1
h8_q10=-1
h8_q11=-1
h8_q12=-1
h8_q13=-1
h8_q14=-1
h8_q15=-1
h8_q16=1,1553.758163,0.085934,117,128,0.00,QQFCILESKLCK,0,01000000000000,0.00,1,0000000000000000000,0,0,0.000000
h8_q16_terms=K,L
h8_q17=-1
h8_q18=2,1901.029175,-0.111452,281,297,0.00,VTNVLITPGKSQMDLRK,0,0001000000001000000,0.00,1,0000000000000000000,0,0,0.000000
h8_q18_terms=R,L
h8_q19=2,1910.065903,0.051280,213,230,0.00,ALQAGPLKKDGPMQITVK,0,00000000000002000000,0.00,1,0000000000000000000,0,0,0.000000
h8_q19_terms=K,D
h8_q20=2,1917.024094,-0.104587,281,297,0.00,VTNVLITPGKSQMDLRK,0,0001000000001200000,0.00,1,0000000000000000000,0,0,0.000000
h8_q20_terms=R,L
h8_q21=-1
h8_q22=-1
h9=FMO5_RABIT,3.72e+001,0.32,60319.98
h9_text=Dimethylaniline monooxygenase [N-oxide-forming] 5 OS=Oryctolagus cuniculus OX=9986 GN=FMO5 PE=2 SV=2
h9_q1=1,577.300598,0.000234,1,5,0.00,MAGKR,0,0200000,0.00,1,0000000000000000000,0,0,0.000000
h9_q1_terms=-,V
h9_q2=1,586.380234,0.009013,345,349,0.00,VVKNK,0,0000000,0.00,1,0000000000000000000,0,0,0.000000
h9_q2_terms=K,V
h9_q3=-1
h9_q4=1,675.348602,-0.014634,260,264,0.00,KMNQR,0,0000000,0.00,1,0000000000000000000,0,0,0.000000
h9_q4_terms=R,F
h9_q5=-1
h9_q6=0,744.431610,-0.034963,497,502,0.00,KPLMTR,0,00000000,0.00,1,0000000000000000000,0,0,0.000000
h9_q6_terms=R,V
h9_q7=-1
h9_q8=-1
h9_q9=0,859.430038,-0.003074,169,175,0.00,GQYLHSR,0,000000000,0.00,1,0000000000000000000,0,0,0.000000
h9_q9_terms=K,D
h9_q10=-1
h9_q11=-1
h9_q12=-1
h9_q13=-1
h9_q14=-1
h9_q15=-1
h9_q16=-1
h9_q17=1,1582.819077,-0.052260,265,277,0.00,FDHAMFGLKPKHR,0,000000000000000,0.00,1,0000000000000000000,0,0,0.000000
h9_q17_terms=R,A
h9_q18=-1
h9_q19=-1
h9_q20=-1
h9_q21=0,2149.174286,0.084130,188,209,0.00,VIVIGIGNSGGDLAVEISHTAK,0,000000000000000000000000,0.00,1,0000000000000000000,0,0,0.000000
h9_q21_terms=R,Q
h9_q22=-1
h10=JDP2_MOUSE,3.71e+001,0.32,18777.01
h10_text=Jun dimerization protein 2 OS=Mus musculus OX=10090 GN=Jdp2 PE=1 SV=2
h10_q1=1,577.264206,0.036626,89,92,0.00,CRNK,0,000100,0.00,1,0000000000000000000,0,0,0.000000
h10_q1_terms=R,K
h10_q2=-1
h10_q3=-1
h10_q4=-1
h10_q5=-1
h10_q6=-1
h10_q7=-1
h10_q8=-1
h10_q9=0,859.465073,-0.038109,116,122,0.00,TQIEELK,0,000000000,0.00,1,0000000000000000000,0,0,0.000000
h10_q9_terms=K,L
h10_q10=-1
h10_q11=-1
h10_q12=-1
h10_q13=2,985.566833,-0.052247,80,88,0.00,EKNKVAAAR,0,00000000000,0.00,1,0000000000000000000,0,0,0.000000
h10_q13_terms=R,C
h10_q14=0,1130.600494,-0.065181,126,134,0.00,QQLILMLNR,0,01100000100,0.00,1,0000000000000000000,0,0,0.000000
h10_q14_terms=R,H
h10_q15=-1
h10_q16=-1
h10_q17=-1
h10_q18=1,1901.017899,-0.100176,107,122,0.00,LELMNAELKTQIEELK,0,000000000000000000,0.00,1,0000000000000000000,0,0,0.000000
h10_q18_terms=R,L
h10_q19=-1
h10_q20=1,1917.012817,-0.093310,107,122,0.00,LELMNAELKTQIEELK,0,000020000000000000,0.00,1,0000000000000000000,0,0,0.000000
h10_q20_terms=R,L
h10_q21=1,2149.161194,0.097222,126,142,0.00,QQLILMLNRHRPTCIVR,0,0110000000000000000,0.00,1,0000000000000000000,0,0,0.000000
h10_q21_terms=R,T
h10_q22=-1
h11=JDP2_RAT,3.71e+001,0.32,18777.01
h11_text=Jun dimerization protein 2 OS=Rattus norvegicus OX=10116 GN=Jdp2 PE=1 SV=1
h11_q1=1,577.264206,0.036626,89,92,0.00,CRNK,0,000100,0.00,1,0000000000000000000,0,0,0.000000
h11_q1_terms=R,K
h11_q2=-1
h11_q3=-1
h11_q4=-1
h11_q5=-1
h11_q6=-1
h11_q7=-1
h11_q8=-1
h11_q9=0,859.465073,-0.038109,116,122,0.00,TQIEELK,0,000000000,0.00,1,0000000000000000000,0,0,0.000000
h11_q9_terms=K,L
h11_q10=-1
h11_q11=-1
h11_q12=-1
h11_q13=2,985.566833,-0.052247,80,88,0.00,EKNKVAAAR,0,00000000000,0.00,1,0000000000000000000,0,0,0.000000
h11_q13_terms=R,C
h11_q14=0,1130.600494,-0.065181,126,134,0.00,QQLILMLNR,0,01100000100,0.00,1,0000000000000000000,0,0,0.000000
h11_q14_terms=R,H
h11_q15=-1
h11_q16=-1
h11_q17=-1
h11_q18=1,1901.017899,-0.100176,107,122,0.00,LELMNAELKTQIEELK,0,000000000000000000,0.00,1,0000000000000000000,0,0,0.000000
h11_q18_terms=R,L
h11_q19=-1
h11_q20=1,1917.012817,-0.093310,107,122,0.00,LELMNAELKTQIEELK,0,000020000000000000,0.00,1,0000000000000000000,0,0,0.000000
h11_q20_terms=R,L
h11_q21=1,2149.161194,0.097222,126,142,0.00,QQLILMLNRHRPTCIVR,0,0110000000000000000,0.00,1,0000000000000000000,0,0,0.000000
h11_q21_terms=R,T
h11_q22=-1
h12=R113A_HUMAN,3.64e+001,0.32,39503.88
h12_text=E3 ubiquitin-protein ligase RNF113A OS=Homo sapiens OX=9606 GN=RNF113A PE=1 SV=1
h12_q1=-1
h12_q2=-1
h12_q3=-1
h12_q4=-1
h12_q5=-1
h12_q6=-1
h12_q7=0,754.325455,0.004806,262,266,0.00,CFICR,0,0000000,0.00,1,0000000000000000000,0,0,0.000000
h12_q7_terms=K,Q
h12_q8=0,836.402802,0.054871,155,161,0.00,GINNYQK,0,000100000,0.00,1,0000000000000000000,0,0,0.000000
h12_q8_terms=R,Y
h12_q9=-1
h12_q10=-1
h12_q11=0,925.440598,0.025302,228,234,0.00,HGWQIER,0,000010000,0.00,1,0000000000000000000,0,0,0.000000
h12_q11_terms=K,E
h12_q12=1,942.574936,0.004918,312,319,0.00,ELIAKLEK,0,0000000000,0.00,1,0000000000000000000,0,0,0.000000
h12_q12_terms=K,H
h12_q13=-1
h12_q14=1,1130.593109,-0.057796,137,145,0.00,SQKIQEELR,0,00100000000,0.00,1,0000000000000000000,0,0,0.000000
h12_q14_terms=R,G
h12_q15=-1
h12_q16=-1
h12_q17=-1
h12_q18=0,1900.808395,0.109328,296,311,0.00,CYVCDQQTNGVFNPAK,0,000000100000000000,0.00,1,0000000000000000000,0,0,0.000000
h12_q18_terms=R,E
h12_q19=-1
h12_q20=2,1916.981781,-0.062274,152,166,0.00,IYRGINNYQKYMKPK,0,00000011000000000,0.00,1,0000000000000000000,0,0,0.000000
h12_q20_terms=K,D
h12_q21=-1
h12_q22=-1
h13=JDP2_HUMAN,3.64e+001,0.32,18806.02
h13_text=Jun dimerization protein 2 OS=Homo sapiens OX=9606 GN=JDP2 PE=1 SV=1
h13_q1=1,577.264206,0.036626,89,92,0.00,CRNK,0,000100,0.00,1,0000000000000000000,0,0,0.000000
h13_q1_terms=R,K
h13_q2=-1
h13_q3=-1
h13_q4=-1
h13_q5=-1
h13_q6=-1
h13_q7=-1
h13_q8=-1
h13_q9=0,859.465073,-0.038109,116,122,0.00,TQIEELK,0,000000000,0.00,1,0000000000000000000,0,0,0.000000
h13_q9_terms=K,Q
h13_q10=-1
h13_q11=-1
h13_q12=-1
h13_q13=2,985.566833,-0.052247,80,88,0.00,EKNKVAAAR,0,00000000000,0.00,1,0000000000000000000,0,0,0.000000
h13_q13_terms=R,C
h13_q14=0,1130.600494,-0.065181,126,134,0.00,QQLILMLNR,0,01100000100,0.00,1,0000000000000000000,0,0,0.000000
h13_q14_terms=R,H
h13_q15=-1
h13_q16=-1
h13_q17=-1
h13_q18=1,1901.017899,-0.100176,107,122,0.00,LELMNAELKTQIEELK,0,000000000000000000,0.00,1,0000000000000000000,0,0,0.000000
h13_q18_terms=R,Q
h13_q19=-1
h13_q20=1,1917.012817,-0.093310,107,122,0.00,LELMNAELKTQIEELK,0,000020000000000000,0.00,1,0000000000000000000,0,0,0.000000
h13_q20_terms=R,Q
h13_q21=1,2149.161194,0.097222,126,142,0.00,QQLILMLNRHRPTCIVR,0,0110000000000000000,0.00,1,0000000000000000000,0,0,0.000000
h13_q21_terms=R,T
h13_q22=-1
h14=CCD63_RAT,3.58e+001,0.50,66180.54
h14_text=Coiled-coil domain-containing protein 63 OS=Rattus norvegicus OX=10116 GN=Ccdc63 PE=2 SV=1
h14_q1=0,577.297241,0.003591,33,36,0.00,QQFR,0,000000,0.00,1,0000000000000000000,0,0,0.000000
h14_q1_terms=R,K
h14_q2=-1
h14_q3=-1
h14_q4=0,675.337372,-0.003404,222,227,0.00,VEAMAR,0,00000000,0.00,1,0000000000000000000,0,0,0.000000
h14_q4_terms=R,M
h14_q5=-1
h14_q6=0,744.401733,-0.005086,59,64,0.00,EIENLK,0,00000000,0.00,1,0000000000000000000,0,0,0.000000
h14_q6_terms=K,A
h14_q7=-1
h14_q8=-1
h14_q9=-1
h14_q10=-1
h14_q11=1,925.436554,0.029346,544,550,0.00,NEEKNHR,0,000000000,0.00,1,0000000000000000000,0,0,0.000000
h14_q11_terms=R,R
h14_q12=2,942.586182,-0.006328,280,287,0.00,QEAVKIKK,0,0000000000,0.00,2,0000000000000000000,0,0,0.000000
h14_q12_terms=K,H
h14_q13=-1
h14_q14=-1
h14_q15=2,1478.715561,0.008802,222,234,0.00,VEAMARMAAMKDR,0,000000000000000,0.00,1,0000000000000000000,0,0,0.000000
h14_q15_terms=R,Q
h14_q16=2,1553.866425,-0.022328,7,20,0.00,KVSEPLPELSEKAK,0,0000000000000000,0.00,1,0000000000000000000,0,0,0.000000
h14_q16_terms=R,E
h14_q17=1,1582.791199,-0.024382,52,64,0.00,MIASQYKEIENLK,0,020001000000000,0.00,1,0000000000000000000,0,0,0.000000
h14_q17_terms=R,A
h14_q18=2,1901.029175,-0.111452,112,127,0.00,VLLGELDDKIVQMERK,0,000000000000020000,0.00,1,0000000000000000000,0,0,0.000000
h14_q18_terms=K,I
h14_q19=1,1910.014893,0.102290,435,450,0.00,ITDSNLQQYFAIIEKK,0,000000000000000000,0.00,1,0000000000000000000,0,0,0.000000
h14_q19_terms=K,T
h14_q20=2,1916.995544,-0.076037,79,94,0.00,SPKNLDLNQKNFTELR,0,000010000000000000,0.00,1,0000000000000000000,0,0,0.000000
h14_q20_terms=K,F
h14_q21=-1
h14_q22=-1
h15=ZNF92_HUMAN,3.56e+001,0.41,70380.98
h15_text=Zinc finger protein 92 OS=Homo sapiens OX=9606 GN=ZNF92 PE=2 SV=2
h15_q1=1,577.264206,0.036626,175,178,0.00,CKNR,0,000100,0.00,1,0000000000000000000,0,0,0.000000
h15_q1_terms=K,G
h15_q2=-1
h15_q3=-1
h15_q4=-1
h15_q5=-1
h15_q6=-1
h15_q7=-1
h15_q8=0,836.421463,0.036210,1,7,0.00,MGPLTFR,0,020000000,0.00,1,0000000000000000000,0,0,0.000000
h15_q8_terms=-,D
h15_q9=-1
h15_q10=-1
h15_q11=-1
h15_q12=-1
h15_q13=-1
h15_q14=0,1130.535599,-0.000286,106,114,0.00,YGHENLQLR,0,00000101000,0.00,1,0000000000000000000,0,0,0.000000
h15_q14_terms=K,K
h15_q15=0,1478.701004,0.023359,181,192,0.00,SFCMLSQLTQHK,0,00000000000000,0.00,1,0000000000000000000,0,0,0.000000
h15_q15_terms=K,K
h15_q16=2,1553.856567,-0.012470,93,105,0.00,DSFQKVILRTYGK,0,000000000000000,0.00,1,0000000000000000000,0,0,0.000000
h15_q16_terms=K,Y
h15_q17=2,1582.767395,-0.000578,443,454,0.00,HKRNHMEDKPYK,0,00001000000000,0.00,1,0000000000000000000,0,0,0.000000
h15_q17_terms=K,C
h15_q18=2,1900.840714,0.077009,231,246,0.00,CEECGKAFNRSSNLTK,0,000000000100000000,0.00,1,0000000000000000000,0,0,0.000000
h15_q18_terms=K,H
h15_q19=-1
h15_q20=1,1916.839676,0.079831,203,218,0.00,CEECGKAFNWSSTLTK,0,000000000000000000,0.00,1,0000000000000000000,0,0,0.000000
h15_q20_terms=K,H
h15_q21=-1
h15_q22=2,2186.148376,-0.051834,489,507,0.00,AFNQSSIFTKHKIIHTEGK,0,000100000000000000000,0.00,1,0000000000000000000,0,0,0.000000
h15_q22_terms=K,S
h16=LSMD1_MOUSE,3.54e+001,0.23,13706.61
h16_text=N-alpha-acetyltransferase 38, NatC auxiliary subunit OS=Mus musculus OX=10090 GN=Naa38 PE=1 SV=1
h16_q1=-1
h16_q2=-1
h16_q3=-1
h16_q4=1,675.384995,-0.051027,52,56,0.00,TMRIR,0,0000000,0.00,1,0000000000000000000,0,0,0.000000
h16_q4_terms=K,M
h16_q5=-1
h16_q6=-1
h16_q7=-1
h16_q8=-1
h16_q9=-1
h16_q10=-1
h16_q11=-1
h16_q12=-1
h16_q13=0,985.537872,-0.023286,2,11,0.00,AGAGPTMLLR,0,000000000000,0.00,1,0000000000000000000,0,0,0.000000
h16_q13_terms=M,E
h16_q14=-1
h16_q15=-1
h16_q16=-1
h16_q17=-1
h16_q18=1,1900.859406,0.058317,57,72,0.00,MTDGRTLVGCFLCTDR,0,000000000000000000,0.00,1,0000000000000000000,0,0,0.000000
h16_q18_terms=R,D
h16_q19=-1
h16_q20=1,1916.854324,0.065183,57,72,0.00,MTDGRTLVGCFLCTDR,0,020000000000000000,0.00,1,0000000000000000000,0,0,0.000000
h16_q20_terms=R,D
h16_q21=-1
h16_q22=2,2186.039490,0.057052,55,72,0.00,IRMTDGRTLVGCFLCTDR,0,00020000000000000000,0.00,1,0000000000000000000,0,0,0.000000
h16_q22_terms=R,D
h17=LSMD1_HUMAN,3.53e+001,0.23,13790.68
h17_text=N-alpha-acetyltransferase 38, NatC auxiliary subunit OS=Homo sapiens OX=9606 GN=NAA38 PE=1 SV=1
h17_q1=-1
h17_q2=-1
h17_q3=-1
h17_q4=1,675.384995,-0.051027,52,56,0.00,TMRIR,0,0000000,0.00,1,0000000000000000000,0,0,0.000000
h17_q4_terms=K,M
h17_q5=-1
h17_q6=-1
h17_q7=-1
h17_q8=-1
h17_q9=-1
h17_q10=-1
h17_q11=-1
h17_q12=-1
h17_q13=0,985.537872,-0.023286,2,11,0.00,AGAGPTMLLR,0,000000000000,0.00,1,0000000000000000000,0,0,0.000000
h17_q13_terms=M,E
h17_q14=-1
h17_q15=-1
h17_q16=-1
h17_q17=-1
h17_q18=1,1900.859406,0.058317,57,72,0.00,MTDGRTLVGCFLCTDR,0,000000000000000000,0.00,1,0000000000000000000,0,0,0.000000
h17_q18_terms=R,D
h17_q19=-1
h17_q20=1,1916.854324,0.065183,57,72,0.00,MTDGRTLVGCFLCTDR,0,020000000000000000,0.00,1,0000000000000000000,0,0,0.000000
h17_q20_terms=R,D
h17_q21=-1
h17_q22=2,2186.039490,0.057052,55,72,0.00,IRMTDGRTLVGCFLCTDR,0,00020000000000000000,0.00,1,0000000000000000000,0,0,0.000000
h17_q22_terms=R,D
h18=DHX30_BOVIN,3.53e+001,0.45,136944.73
h18_text=ATP-dependent RNA helicase DHX30 OS=Bos taurus OX=9913 GN=DHX30 PE=2 SV=1
h18_q1=-1
h18_q2=-1
h18_q3=-1
h18_q4=0,675.318756,0.015212,1154,1159,0.00,LEGDSR,0,00000000,0.00,1,0000000000000000000,0,0,0.000000
h18_q4_terms=R,T
h18_q5=1,687.402771,0.040837,1061,1066,0.00,VTRQGK,0,00000000,0.00,2,0000000000000000000,0,0,0.000000
h18_q5_terms=K,F
h18_q6=0,744.380630,0.016017,75,80,0.00,EFPQPK,0,00000000,0.00,1,0000000000000000000,0,0,0.000000
h18_q6_terms=K,N
h18_q7=-1
h18_q8=0,836.404160,0.053513,659,664,0.00,HQYPHR,0,00000000,0.00,1,0000000000000000000,0,0,0.000000
h18_q8_terms=K,H
h18_q9=-1
h18_q10=-1
h18_q11=-1
h18_q12=-1
h18_q13=0,985.555618,-0.041032,81,89,0.00,NLLNSVIGR,0,01000000000,0.00,1,0000000000000000000,0,0,0.000000
h18_q13_terms=K,A
h18_q14=1,1130.548187,-0.012874,659,666,0.00,HQYPHRHR,0,0010000000,0.00,1,0000000000000000000,0,0,0.000000
h18_q14_terms=K,H
h18_q15=-1
h18_q16=1,1553.918076,-0.073979,264,277,0.00,ALTQFPLPKNLLAK,0,0000100000000000,0.00,1,0000000000000000000,0,0,0.000000
h18_q16_terms=R,V
h18_q17=-1
h18_q18=0,1900.939316,-0.021593,692,708,0.00,GEPGGILCFLPGWQEIK,0,0000000000000010000,0.00,1,0000000000000000000,0,0,0.000000
h18_q18_terms=R,G
h18_q19=2,1910.033325,0.083858,1078,1094,0.00,SGNILLHKSTINREATR,0,0001000000000000000,0.00,1,0000000000000000000,0,0,0.000000
h18_q19_terms=K,L
h18_q20=-1
h18_q21=1,2149.123947,0.134469,193,213,0.00,QLNPESIRPGGPGGLSRSLGR,0,01010000000000000000000,0.00,1,0000000000000000000,0,0,0.000000
h18_q21_terms=R,E
h18_q22=-1
h19=SFI1_CALJA,3.49e+001,0.50,148014.12
h19_text=Protein SFI1 homolog OS=Callithrix jacchus OX=9483 GN=SFI1 PE=3 SV=1
h19_q1=1,577.300598,0.000234,178,181,0.00,QKMR,0,000200,0.00,1,0000000000000000000,0,0,0.000000
h19_q1_terms=K,Q
h19_q2=0,586.343842,0.045405,224,228,0.00,LGQIR,0,0001000,0.00,1,0000000000000000000,0,0,0.000000
h19_q2_terms=R,V
h19_q3=-1
h19_q4=1,675.373749,-0.039781,12,16,0.00,MIKQR,0,0000100,0.00,1,0000000000000000000,0,0,0.000000
h19_q4_terms=K,M
h19_q5=-1
h19_q6=1,744.399063,-0.002416,760,765,0.00,RSAQQR,0,00000000,0.00,1,0000000000000000000,0,0,0.000000
h19_q6_terms=R,L
h19_q7=-1
h19_q8=-1
h19_q9=-1
h19_q10=-1
h19_q11=0,925.498123,-0.032223,1094,1102,0.00,VSAQPATPR,0,00000000000,0.00,1,0000000000000000000,0,0,0.000000
h19_q11_terms=R,H
h19_q12=-1
h19_q13=1,985.545715,-0.031129,1175,1181,0.00,RWLELNR,0,000000000,0.00,1,0000000000000000000,0,0,0.000000
h19_q13_terms=R,E
h19_q14=-1
h19_q15=-1
h19_q16=1,1553.846008,-0.001911,1218,1231,0.00,QPTGACIARIQALR,0,0000000000000000,0.00,2,0000000000000000000,0,0,0.000000
h19_q16_terms=R,Q
h19_q17=-1
h19_q18=2,1900.999496,-0.081773,215,228,0.00,VWWSMWRQRLGQIR,0,0000000000000000,0.00,1,0000000000000000000,0,0,0.000000
h19_q18_terms=R,V
h19_q19=-1
h19_q20=2,1916.994415,-0.074908,215,228,0.00,VWWSMWRQRLGQIR,0,0000020000000000,0.00,1,0000000000000000000,0,0,0.000000
h19_q20_terms=R,V
h19_q21=2,2149.088379,0.170037,107,122,0.00,FYYEQQLLRKIFGEWK,0,000001100000000000,0.00,1,0000000000000000000,0,0,0.000000
h19_q21_terms=R,E
h19_q22=2,2186.091553,0.004989,812,828,0.00,LSQTCFHQWRQQLAARR,0,0001000000000000000,0.00,1,0000000000000000000,0,0,0.000000
h19_q22_terms=R,Q
h20=FMO5_MOUSE,3.38e+001,0.32,60532.22
h20_text=Dimethylaniline monooxygenase [N-oxide-forming] 5 OS=Mus musculus OX=10090 GN=Fmo5 PE=1 SV=4
h20_q1=-1
h20_q2=1,586.380234,0.009013,345,349,0.00,VVKNK,0,0000000,0.00,1,0000000000000000000,0,0,0.000000
h20_q2_terms=K,V
h20_q3=-1
h20_q4=0,675.312225,0.021743,260,264,0.00,QMNQR,0,0000000,0.00,1,0000000000000000000,0,0,0.000000
h20_q4_terms=K,F
h20_q5=-1
h20_q6=0,744.431610,-0.034963,497,502,0.00,KPLMTR,0,00000000,0.00,1,0000000000000000000,0,0,0.000000
h20_q6_terms=R,V
h20_q7=-1
h20_q8=-1
h20_q9=-1
h20_q10=-1
h20_q11=-1
h20_q12=-1
h20_q13=1,985.545715,-0.031129,217,224,0.00,RGAWILNR,0,0000000100,0.00,1,0000000000000000000,0,0,0.000000
h20_q13_terms=R,V
h20_q14=-1
h20_q15=0,1478.663223,0.061140,399,411,0.00,LPSQSEMMAEINK,0,000010000000100,0.00,1,0000000000000000000,0,0,0.000000
h20_q15_terms=K,A
h20_q16=1,1553.721756,0.122341,247,259,0.00,ICGPSLKNNYMEK,0,000000001000000,0.00,1,0000000000000000000,0,0,0.000000
h20_q16_terms=R,Q
h20_q17=-1
h20_q18=-1
h20_q19=-1
h20_q20=-1
h20_q21=0,2149.174286,0.084130,188,209,0.00,VIVIGIGNSGGDLAVEISHTAK,0,000000000000000000000000,0.00,1,0000000000000000000,0,0,0.000000
h20_q21_terms=R,Q
h20_q22=-1
h21=ILKAP_RAT,3.35e+001,0.27,43287.93
h21_text=Integrin-linked kinase-associated serine/threonine phosphatase 2C OS=Rattus norvegicus OX=10116 GN=Ilkap PE=2 SV=1
h21_q1=-1
h21_q2=-1
h21_q3=-1
h21_q4=1,675.373749,-0.039781,269,273,0.00,MRIQK,0,0000100,0.00,1,0000000000000000000,0,0,0.000000
h21_q4_terms=R,A
h21_q5=-1
h21_q6=-1
h21_q7=-1
h21_q8=-1
h21_q9=2,859.451141,-0.024177,72,79,0.00,NEGKGAKR,0,0100000000,0.00,1,0000000000000000000,0,0,0.000000
h21_q9_terms=K,K
h21_q10=-1
h21_q11=-1
h21_q12=1,942.524658,0.055196,271,279,0.00,IQKAGGNVR,0,00100000000,0.00,1,0000000000000000000,0,0,0.000000
h21_q12_terms=R,D
h21_q13=2,985.519211,-0.004625,79,86,0.00,RKAPEEEK,0,0000000000,0.00,1,0000000000000000000,0,0,0.000000
h21_q13_terms=K,N
h21_q14=0,1130.571991,-0.036678,205,214,0.00,QASSQKPAWK,0,010000000000,0.00,1,0000000000000000000,0,0,0.000000
h21_q14_terms=K,D
h21_q15=-1
h21_q16=1,1553.831375,0.012722,162,174,0.00,FAAQNLHQNLIRK,0,000011000000000,0.00,1,0000000000000000000,0,0,0.000000
h21_q16_terms=K,F
h21_q17=-1
h21_q18=-1
h21_q19=-1
h21_q20=-1
h21_q21=-1
h21_q22=-1
h22=DDIT3_HUMAN,3.33e+001,0.18,19163.26
h22_text=DNA damage-inducible transcript 3 protein OS=Homo sapiens OX=9606 GN=DDIT3 PE=1 SV=1
h22_q1=1,577.300598,0.000234,118,121,0.00,QRMK,0,000200,0.00,1,0000000000000000000,0,0,0.000000
h22_q1_terms=K,E
h22_q2=0,586.343842,0.045405,158,162,0.00,ALIDR,0,0000000,0.00,1,0000000000000000000,0,0,0.000000
h22_q2_terms=R,M
h22_q3=-1
h22_q4=-1
h22_q5=-1
h22_q6=-1
h22_q7=-1
h22_q8=-1
h22_q9=1,859.451157,-0.024193,151,157,0.00,EVEATRR,0,000000000,0.00,1,0000000000000000000,0,0,0.000000
h22_q9_terms=R,A
h22_q10=-1
h22_q11=-1
h22_q12=-1
h22_q13=-1
h22_q14=-1
h22_q15=-1
h22_q16=2,1553.798370,0.045727,157,169,0.00,RALIDRMVNLHQA,0,000000020100100,0.00,1,0000000000000000000,0,0,0.000000
h22_q16_terms=R,-
h22_q17=-1
h22_q18=-1
h22_q19=-1
h22_q20=-1
h22_q21=-1
h22_q22=-1
h23=Z280C_HUMAN,3.29e+001,0.41,84866.97
h23_text=Zinc finger protein 280C OS=Homo sapiens OX=9606 GN=ZNF280C PE=1 SV=1
h23_q1=0,577.268234,0.032598,254,257,0.00,YHMK,0,000000,0.00,1,0000000000000000000,0,0,0.000000
h23_q1_terms=K,H
h23_q2=-1
h23_q3=-1
h23_q4=-1
h23_q5=-1
h23_q6=0,744.401764,-0.005117,181,187,0.00,VNSVTPK,0,001000000,0.00,1,0000000000000000000,0,0,0.000000
h23_q6_terms=K,K
h23_q7=-1
h23_q8=0,836.464355,-0.006682,477,483,0.00,LQFLTSK,0,001000000,0.00,1,0000000000000000000,0,0,0.000000
h23_q8_terms=R,E
h23_q9=-1
h23_q10=-1
h23_q11=2,925.502823,-0.036923,614,620,0.00,CRRGIHK,0,000000000,0.00,1,0000000000000000000,0,0,0.000000
h23_q11_terms=R,C
h23_q12=-1
h23_q13=1,985.512711,0.001875,702,709,0.00,MAKHLSQR,0,0200000000,0.00,1,0000000000000000000,0,0,0.000000
h23_q13_terms=R,K
h23_q14=-1
h23_q15=1,1478.762527,-0.038164,441,452,0.00,NLLCPFCLKVSK,0,01000000000000,0.00,1,0000000000000000000,0,0,0.000000
h23_q15_terms=K,M
h23_q16=1,1553.871185,-0.027088,676,689,0.00,HSGTLRGITLVCLK,0,0000000000000000,0.00,1,0000000000000000000,0,0,0.000000
h23_q16_terms=K,C
h23_q17=-1
h23_q18=-1
h23_q19=-1
h23_q20=1,1916.912415,0.007092,434,449,0.00,AAHENTKNLLCPFCLK,0,000001001000000000,0.00,2,0000000000000000000,0,0,0.000000
h23_q20_terms=R,V
h23_q21=-1
h23_q22=2,2186.169525,-0.072983,168,187,0.00,NTSYVLKHPSTSKVNSVTPK,0,0000000000000000000000,0.00,1,0000000000000000000,0,0,0.000000
h23_q22_terms=K,K
h24=ILKAP_MOUSE,3.28e+001,0.27,43317.99
h24_text=Integrin-linked kinase-associated serine/threonine phosphatase 2C OS=Mus musculus OX=10090 GN=Ilkap PE=1 SV=1
h24_q1=-1
h24_q2=-1
h24_q3=-1
h24_q4=1,675.373749,-0.039781,269,273,0.00,MRIQK,0,0000100,0.00,1,0000000000000000000,0,0,0.000000
h24_q4_terms=R,A
h24_q5=-1
h24_q6=-1
h24_q7=-1
h24_q8=-1
h24_q9=0,859.465088,-0.038124,178,185,0.00,GDIISVEK,0,0000000000,0.00,1,0000000000000000000,0,0,0.000000
h24_q9_terms=K,T
h24_q10=-1
h24_q11=-1
h24_q12=1,942.524658,0.055196,271,279,0.00,IQKAGGNVR,0,00100000000,0.00,1,0000000000000000000,0,0,0.000000
h24_q12_terms=R,D
h24_q13=2,985.519211,-0.004625,79,86,0.00,RKAPEEEK,0,0000000000,0.00,1,0000000000000000000,0,0,0.000000
h24_q13_terms=K,N
h24_q14=0,1130.571991,-0.036678,205,214,0.00,QASSQKPAWK,0,010000000000,0.00,1,0000000000000000000,0,0,0.000000
h24_q14_terms=K,D
h24_q15=-1
h24_q16=1,1553.831375,0.012722,162,174,0.00,FAAQNLHQNLIRK,0,000011000000000,0.00,1,0000000000000000000,0,0,0.000000
h24_q16_terms=K,F
h24_q17=-1
h24_q18=-1
h24_q19=-1
h24_q20=-1
h24_q21=-1
h24_q22=-1
h25=IL5_HORSE,3.27e+001,0.18,15242.00
h25_text=Interleukin-5 OS=Equus caballus OX=9796 GN=IL5 PE=2 SV=1
h25_q1=-1
h25_q2=-1
h25_q3=-1
h25_q4=-1
h25_q5=-1
h25_q6=-1
h25_q7=-1
h25_q8=0,836.427963,0.029710,96,102,0.00,GYIDLQK,0,000000100,0.00,1,0000000000000000000,0,0,0.000000
h25_q8_terms=K,K
h25_q9=-1
h25_q10=1,919.408264,-0.052121,105,111,0.00,CGGERWR,0,000000000,0.00,1,0000000000000000000,0,0,0.000000
h25_q10_terms=K,V
h25_q11=-1
h25_q12=-1
h25_q13=-1
h25_q14=0,1130.556763,-0.021450,76,86,0.00,NQTVQGDAVAK,0,0100000000000,0.00,1,0000000000000000000,0,0,0.000000
h25_q14_terms=K,L
h25_q15=-1
h25_q16=-1
h25_q17=-1
h25_q18=-1
h25_q19=-1
h25_q20=-1
h25_q21=-1
h25_q22=1,2186.205902,-0.109360,76,95,0.00,NQTVQGDAVAKLFQNLSLIK,0,0000000000000000000000,0.00,1,0000000000000000000,0,0,0.000000
h25_q22_terms=K,G
h26=RBL2_HUMAN,3.26e+001,0.45,129711.15
h26_text=Retinoblastoma-like protein 2 OS=Homo sapiens OX=9606 GN=RBL2 PE=1 SV=3
h26_q1=-1
h26_q2=2,586.366302,0.022945,189,192,0.00,KQRR,0,000000,0.00,1,0000000000000000000,0,0,0.000000
h26_q2_terms=R,Q
h26_q3=-1
h26_q4=-1
h26_q5=-1
h26_q6=-1
h26_q7=-1
h26_q8=-1
h26_q9=-1
h26_q10=0,919.406891,-0.050748,56,63,0.00,LNMDEAAR,0,0010000000,0.00,1,0000000000000000000,0,0,0.000000
h26_q10_terms=R,A
h26_q11=0,925.396362,0.069538,49,55,0.00,FDELCSR,0,000000000,0.00,1,0000000000000000000,0,0,0.000000
h26_q11_terms=R,L
h26_q12=0,942.549835,0.030019,399,407,0.00,ISTPLTGVR,0,00000000000,0.00,1,0000000000000000000,0,0,0.000000
h26_q12_terms=R,Y
h26_q13=0,985.482849,0.031737,454,462,0.00,DPTQAIANR,0,00001000000,0.00,1,0000000000000000000,0,0,0.000000
h26_q13_terms=R,L
h26_q14=1,1130.622971,-0.087658,1085,1093,0.00,LREINSMIR,0,00000000000,0.00,1,0000000000000000000,0,0,0.000000
h26_q14_terms=R,T
h26_q15=-1
h26_q16=-1
h26_q17=-1
h26_q18=2,1900.978851,-0.061128,438,453,0.00,NAPSEKLEQILRTCSR,0,000000000000000000,0.00,1,0000000000000000000,0,0,0.000000
h26_q18_terms=R,D
h26_q19=2,1910.026138,0.091045,149,164,0.00,TERLERNFTVSAVIFK,0,000000010000000000,0.00,1,0000000000000000000,0,0,0.000000
h26_q19_terms=R,K
h26_q20=0,1916.893814,0.025693,514,530,0.00,LGDMDLSGILEQDAFHR,0,0000000000001000000,0.00,1,0000000000000000000,0,0,0.000000
h26_q20_terms=R,S
h26_q21=1,2149.109756,0.148660,887,904,0.00,HLDQLLMCAIYVMAKVTK,0,00000002000000000000,0.00,1,0000000000000000000,0,0,0.000000
h26_q21_terms=R,E
h26_q22=-1
h27=SDC2_MOUSE,3.21e+001,0.18,22174.11
h27_text=Syndecan-2 OS=Mus musculus OX=10090 GN=Sdc2 PE=1 SV=1
h27_q1=2,577.336975,-0.036143,172,175,0.00,MRKK,0,020000,0.00,1,0000000000000000000,0,0,0.000000
h27_q1_terms=R,D
h27_q2=-1
h27_q3=-1
h27_q4=-1
h27_q5=-1
h27_q6=-1
h27_q7=-1
h27_q8=0,836.431351,0.026322,87,93,0.00,VETMTLK,0,000020000,0.00,1,0000000000000000000,0,0,0.000000
h27_q8_terms=K,T
h27_q9=0,859.418808,0.008156,136,142,0.00,HSDNLFK,0,000000000,0.00,1,0000000000000000000,0,0,0.000000
h27_q9_terms=K,R
h27_q10=-1
h27_q11=1,925.454514,0.011386,195,202,0.00,APTKEFYA,0,0000000000,0.00,1,0000000000000000000,0,0,0.000000
h27_q11_terms=K,-
h27_q12=-1
h27_q13=-1
h27_q14=-1
h27_q15=-1
h27_q16=-1
h27_q17=-1
h27_q18=-1
h27_q19=-1
h27_q20=-1
h27_q21=-1
h27_q22=-1
h28=C1D_CRIGR,3.17e+001,0.23,15935.22
h28_text=Nuclear nucleic acid-binding protein C1D OS=Cricetulus griseus OX=10029 GN=C1D PE=2 SV=1
h28_q1=-1
h28_q2=-1
h28_q3=-1
h28_q4=-1
h28_q5=-1
h28_q6=0,744.401733,-0.005086,41,46,0.00,NELLQK,0,01000000,0.00,1,0000000000000000000,0,0,0.000000
h28_q6_terms=R,L
h28_q7=-1
h28_q8=-1
h28_q9=0,859.392288,0.034676,129,136,0.00,NTPNVANK,0,0100100100,0.00,1,0000000000000000000,0,0,0.000000
h28_q9_terms=K,G
h28_q10=-1
h28_q11=1,925.469116,-0.003216,92,98,0.00,VYMNRVK,0,000210000,0.00,1,0000000000000000000,0,0,0.000000
h28_q11_terms=R,E
h28_q12=1,942.524643,0.055211,85,91,0.00,QELERIR,0,000000000,0.00,1,0000000000000000000,0,0,0.000000
h28_q12_terms=K,V
h28_q13=-1
h28_q14=-1
h28_q15=-1
h28_q16=1,1553.742889,0.101208,34,46,0.00,TMMSVSRNELLQK,0,002000001000100,0.00,1,0000000000000000000,0,0,0.000000
h28_q16_terms=K,L
h28_q17=-1
h28_q18=-1
h28_q19=-1
h28_q20=-1
h28_q21=-1
h28_q22=-1
h29=RM09_PAPAN,3.17e+001,0.27,30628.62
h29_text=39S ribosomal protein L9, mitochondrial OS=Papio anubis OX=9555 GN=MRPL9 PE=3 SV=1
h29_q1=0,577.301270,-0.000438,192,195,0.00,HFFK,0,000000,0.00,1,0000000000000000000,0,0,0.000000
h29_q1_terms=R,N
h29_q2=-1
h29_q3=-1
h29_q4=0,675.362534,-0.028566,172,177,0.00,LEVGMK,0,00000000,0.00,1,0000000000000000000,0,0,0.000000
h29_q4_terms=R,N
h29_q5=-1
h29_q6=0,744.413010,-0.016363,109,115,0.00,GDLVSVR,0,000000000,0.00,1,0000000000000000000,0,0,0.000000
h29_q6_terms=R,K
h29_q7=-1
h29_q8=-1
h29_q9=0,859.410950,0.016014,260,267,0.00,MAPTSPQI,0,0200000000,0.00,1,0000000000000000000,0,0,0.000000
h29_q9_terms=R,-
h29_q10=-1
h29_q11=-1
h29_q12=-1
h29_q13=1,985.566849,-0.052263,12,20,0.00,ALLRAGTER,0,00000000000,0.00,1,0000000000000000000,0,0,0.000000
h29_q13_terms=R,L
h29_q14=1,1130.611755,-0.076442,172,181,0.00,LEVGMKNNVK,0,000000000000,0.00,1,0000000000000000000,0,0,0.000000
h29_q14_terms=R,W
h29_q15=-1
h29_q16=-1
h29_q17=-1
h29_q18=-1
h29_q19=-1
h29_q20=-1
h29_q21=-1
h29_q22=-1
h30=PKHG6_MOUSE,3.15e+001,0.41,89435.22
h30_text=Pleckstrin homology domain-containing family G member 6 OS=Mus musculus OX=10090 GN=Plekhg6 PE=2 SV=2
h30_q1=-1
h30_q2=-1
h30_q3=-1
h30_q4=-1
h30_q5=2,687.402740,0.040868,445,450,0.00,KADRAK,0,00000000,0.00,1,0000000000000000000,0,0,0.000000
h30_q5_terms=R,V
h30_q6=1,744.449371,-0.052724,97,102,0.00,LKEVTR,0,00000000,0.00,1,0000000000000000000,0,0,0.000000
h30_q6_terms=K,A
h30_q7=-1
h30_q8=-1
h30_q9=0,859.403519,0.023445,739,745,0.00,EELANQR,0,000001000,0.00,1,0000000000000000000,0,0,0.000000
h30_q9_terms=R,I
h30_q10=-1
h30_q11=0,925.425339,0.040561,601,608,0.00,DSHSPLNR,0,0000000100,0.00,1,0000000000000000000,0,0,0.000000
h30_q11_terms=K,L
h30_q12=0,942.549805,0.030049,762,769,0.00,LTLAQLQR,0,0000010000,0.00,1,0000000000000000000,0,0,0.000000
h30_q12_terms=R,M
h30_q13=-1
h30_q14=-1
h30_q15=-1
h30_q16=-1
h30_q17=1,1582.802475,-0.035658,68,81,0.00,GMSPLVLREPDPEK,0,0020000000000000,0.00,1,0000000000000000000,0,0,0.000000
h30_q17_terms=R,R
h30_q18=2,1900.942444,-0.024721,731,745,0.00,EDMLREIREELANQR,0,00000000000000000,0.00,1,0000000000000000000,0,0,0.000000
h30_q18_terms=R,I
h30_q19=-1
h30_q20=2,1916.937363,-0.017856,731,745,0.00,EDMLREIREELANQR,0,00020000000000000,0.00,1,0000000000000000000,0,0,0.000000
h30_q20_terms=R,I
h30_q21=-1
h30_q22=2,2186.087723,0.008819,110,127,0.00,LHTFSMFGMPRLPPEDRR,0,00000000000000000000,0.00,1,0000000000000000000,0,0,0.000000
h30_q22_terms=R,H
h31=IMA1_MOUSE,3.09e+001,0.27,58234.28
h31_text=Importin subunit alpha-1 OS=Mus musculus OX=10090 GN=Kpna2 PE=1 SV=2
h31_q1=-1
h31_q2=0,586.369003,0.020244,198,202,0.00,DLVIK,0,0000000,0.00,1,0000000000000000000,0,0,0.000000
h31_q2_terms=R,H
h31_q3=-1
h31_q4=0,675.343903,-0.009935,454,459,0.00,LGETEK,0,00000000,0.00,1,0000000000000000000,0,0,0.000000
h31_q4_terms=K,L
h31_q5=-1
h31_q6=1,744.449356,-0.052709,103,108,0.00,LLSREK,0,00000000,0.00,1,0000000000000000000,0,0,0.000000
h31_q6_terms=K,Q
h31_q7=-1
h31_q8=1,836.439209,0.018464,389,395,0.00,ADFKTQK,0,000000000,0.00,1,0000000000000000000,0,0,0.000000
h31_q8_terms=K,E
h31_q9=-1
h31_q10=-1
h31_q11=-1
h31_q12=-1
h31_q13=-1
h31_q14=-1
h31_q15=-1
h31_q16=-1
h31_q17=-1
h31_q18=-1
h31_q19=-1
h31_q20=0,1916.907501,0.012006,84,101,0.00,GINSNNLESQLQATQAAR,0,00010110000000000000,0.00,2,0000000000000000000,0,0,0.000000
h31_q20_terms=K,K
h31_q21=-1
h31_q22=1,2186.194641,-0.098099,239,258,0.00,NKNPAPPLDAVEQILPTLVR,0,0101000000000000000000,0.00,1,0000000000000000000,0,0,0.000000
h31_q22_terms=R,L
h32=EXOC6_MOUSE,3.09e+001,0.36,93872.48
h32_text=Exocyst complex component 6 OS=Mus musculus OX=10090 GN=Exoc6 PE=1 SV=2
h32_q1=1,577.307129,-0.006297,762,766,0.00,DTSKK,0,0000000,0.00,1,0000000000000000000,0,0,0.000000
h32_q1_terms=K,N
h32_q2=-1
h32_q3=-1
h32_q4=1,675.293594,0.040374,776,780,0.00,NDRDR,0,0100000,0.00,1,0000000000000000000,0,0,0.000000
h32_q4_terms=K,Q
h32_q5=-1
h32_q6=-1
h32_q7=-1
h32_q8=2,836.442566,0.015107,760,766,0.00,MKDTSKK,0,000000000,0.00,1,0000000000000000000,0,0,0.000000
h32_q8_terms=K,N
h32_q9=1,859.444641,-0.017677,116,121,0.00,CRIQQR,0,00000000,0.00,1,0000000000000000000,0,0,0.000000
h32_q9_terms=R,N
h32_q10=-1
h32_q11=1,925.399704,0.066196,145,151,0.00,EQMSMKR,0,001200000,0.00,1,0000000000000000000,0,0,0.000000
h32_q11_terms=K,Y
h32_q12=-1
h32_q13=-1
h32_q14=-1
h32_q15=-1
h32_q16=-1
h32_q17=0,1582.770096,-0.003279,357,369,0.00,VYTEELWNMALSK,0,000000000000000,0.00,1,0000000000000000000,0,0,0.000000
h32_q17_terms=R,I
h32_q18=2,1900.883591,0.034132,208,224,0.00,HSDKIGEAAMKQAQQQK,0,0000000000001011100,0.00,1,0000000000000000000,0,0,0.000000
h32_q18_terms=K,S
h32_q19=-1
h32_q20=2,1916.878510,0.040997,208,224,0.00,HSDKIGEAAMKQAQQQK,0,0000000000201011100,0.00,1,0000000000000000000,0,0,0.000000
h32_q20_terms=K,S
h32_q21=-1
h32_q22=-1
h33=THEGL_RAT,3.06e+001,0.27,51548.37
h33_text=Testicular haploid expressed gene protein-like OS=Rattus norvegicus OX=10116 GN=Thegl PE=2 SV=1
h33_q1=-1
h33_q2=-1
h33_q3=-1
h33_q4=-1
h33_q5=-1
h33_q6=-1
h33_q7=-1
h33_q8=-1
h33_q9=1,859.401047,0.025917,169,174,0.00,CFYSRK,0,00000000,0.00,2,0000000000000000000,0,0,0.000000
h33_q9_terms=K,R
h33_q10=0,919.424683,-0.068540,81,89,0.00,SVNSSDSPK,0,00000000000,0.00,1,0000000000000000000,0,0,0.000000
h33_q10_terms=K,A
h33_q11=0,925.523254,-0.057354,447,454,0.00,ELAEPIVR,0,0000000000,0.00,1,0000000000000000000,0,0,0.000000
h33_q11_terms=K,-
h33_q12=0,942.513428,0.066426,176,183,0.00,VQDLSRPK,0,0010000000,0.00,1,0000000000000000000,0,0,0.000000
h33_q12_terms=R,K
h33_q13=-1
h33_q14=-1
h33_q15=-1
h33_q16=-1
h33_q17=-1
h33_q18=-1
h33_q19=2,1910.012238,0.104945,176,191,0.00,VQDLSRPKKQWGTPDR,0,000000000000000000,0.00,1,0000000000000000000,0,0,0.000000
h33_q19_terms=R,R
h33_q20=-1
h33_q21=-1
h33_q22=2,2186.183411,-0.086869,299,318,0.00,LSIAKGTDPNYIPPKTIQTK,0,0000000000100000001000,0.00,1,0000000000000000000,0,0,0.000000
h33_q22_terms=R,I
h34=MBNL2_HUMAN,3.03e+001,0.23,41517.56
h34_text=Muscleblind-like protein 2 OS=Homo sapiens OX=9606 GN=MBNL2 PE=1 SV=2
h34_q1=0,577.297241,0.003591,21,24,0.00,QFQR,0,000000,0.00,1,0000000000000000000,0,0,0.000000
h34_q1_terms=R,G
h34_q2=-1
h34_q3=-1
h34_q4=0,675.337387,-0.003419,179,183,0.00,LEVCR,0,0000000,0.00,1,0000000000000000000,0,0,0.000000
h34_q4_terms=K,E
h34_q5=-1
h34_q6=1,744.412964,-0.016317,281,286,0.00,RQALEK,0,00100000,0.00,2,0000000000000000000,0,0,0.000000
h34_q6_terms=K,S
h34_q7=-1
h34_q8=-1
h34_q9=0,859.428665,-0.001701,85,91,0.00,NNLIQQK,0,011001000,0.00,1,0000000000000000000,0,0,0.000000
h34_q9_terms=R,T
h34_q10=-1
h34_q11=-1
h34_q12=-1
h34_q13=0,985.537872,-0.023286,1,9,0.00,MALNVAPVR,0,02000000000,0.00,1,0000000000000000000,0,0,0.000000
h34_q13_terms=-,D
h34_q14=-1
h34_q15=-1
h34_q16=-1
h34_q17=-1
h34_q18=-1
h34_q19=-1
h34_q20=-1
h34_q21=-1
h34_q22=-1
h35=MBNL2_PONAB,3.03e+001,0.23,41517.56
h35_text=Muscleblind-like protein 2 OS=Pongo abelii OX=9601 GN=MBNL2 PE=2 SV=2
h35_q1=0,577.297241,0.003591,21,24,0.00,QFQR,0,000000,0.00,1,0000000000000000000,0,0,0.000000
h35_q1_terms=R,G
h35_q2=-1
h35_q3=-1
h35_q4=0,675.337387,-0.003419,179,183,0.00,LEVCR,0,0000000,0.00,1,0000000000000000000,0,0,0.000000
h35_q4_terms=K,E
h35_q5=-1
h35_q6=1,744.412964,-0.016317,281,286,0.00,RQALEK,0,00100000,0.00,2,0000000000000000000,0,0,0.000000
h35_q6_terms=K,S
h35_q7=-1
h35_q8=-1
h35_q9=0,859.428665,-0.001701,85,91,0.00,NNLIQQK,0,011001000,0.00,1,0000000000000000000,0,0,0.000000
h35_q9_terms=R,T
h35_q10=-1
h35_q11=-1
h35_q12=-1
h35_q13=0,985.537872,-0.023286,1,9,0.00,MALNVAPVR,0,02000000000,0.00,1,0000000000000000000,0,0,0.000000
h35_q13_terms=-,D
h35_q14=-1
h35_q15=-1
h35_q16=-1
h35_q17=-1
h35_q18=-1
h35_q19=-1
h35_q20=-1
h35_q21=-1
h35_q22=-1
h36=DDAH1_HUMAN,3.03e+001,0.23,31444.06
h36_text=N(G),N(G)-dimethylarginine dimethylaminohydrolase 1 OS=Homo sapiens OX=9606 GN=DDAH1 PE=1 SV=3
h36_q1=-1
h36_q2=-1
h36_q3=-1
h36_q4=1,675.329971,0.003997,145,149,0.00,RTNQR,0,0001100,0.00,1,0000000000000000000,0,0,0.000000
h36_q4_terms=K,G
h36_q5=-1
h36_q6=-1
h36_q7=-1
h36_q8=-1
h36_q9=-1
h36_q10=-1
h36_q11=0,925.490921,-0.025021,137,144,0.00,EFFVGLSK,0,0000000000,0.00,1,0000000000000000000,0,0,0.000000
h36_q11_terms=R,R
h36_q12=-1
h36_q13=-1
h36_q14=-1
h36_q15=-1
h36_q16=-1
h36_q17=2,1582.810333,-0.043516,137,149,0.00,EFFVGLSKRTNQR,0,000000000001100,0.00,1,0000000000000000000,0,0,0.000000
h36_q17_terms=R,G
h36_q18=1,1900.963791,-0.046068,252,267,0.00,LKDHMLIPVSMSELEK,0,000002000002000000,0.00,1,0000000000000000000,0,0,0.000000
h36_q18_terms=K,V
h36_q19=2,1909.967255,0.149928,238,253,0.00,TPEEYPESAKVYEKLK,0,000000000000000000,0.00,1,0000000000000000000,0,0,0.000000
h36_q19_terms=R,D
h36_q20=-1
h36_q21=-1
h36_q22=-1
h37=PPM1K_BOVIN,3.02e+001,0.23,41524.00
h37_text=Protein phosphatase 1K, mitochondrial OS=Bos taurus OX=9913 GN=PPM1K PE=2 SV=1
h37_q1=-1
h37_q2=-1
h37_q3=-1
h37_q4=1,675.318741,0.015227,232,236,0.00,DEKER,0,0000000,0.00,1,0000000000000000000,0,0,0.000000
h37_q4_terms=K,I
h37_q5=1,687.410141,0.033467,216,221,0.00,KGKPMK,0,00000000,0.00,1,0000000000000000000,0,0,0.000000
h37_q5_terms=R,L
h37_q6=-1
h37_q7=-1
h37_q8=-1
h37_q9=0,859.465088,-0.038124,263,270,0.00,SLGDLDLK,0,0000000000,0.00,1,0000000000000000000,0,0,0.000000
h37_q9_terms=R,T
h37_q10=-1
h37_q11=-1
h37_q12=-1
h37_q13=-1
h37_q14=0,1130.581909,-0.046596,271,281,0.00,TSGVIAEPETK,0,0000000000000,0.00,1,0000000000000000000,0,0,0.000000
h37_q14_terms=K,R
h37_q15=1,1478.704132,0.020231,352,363,0.00,YKNSEITFSFSR,0,00010000000000,0.00,1,0000000000000000000,0,0,0.000000
h37_q15_terms=K,S
h37_q16=-1
h37_q17=-1
h37_q18=-1
h37_q19=-1
h37_q20=-1
h37_q21=-1
h37_q22=-1
h38=WHAMM_MOUSE,3.00e+001,0.41,89967.66
h38_text=WASP homolog-associated protein with actin, membranes and microtubules OS=Mus musculus OX=10090 GN=Whamm PE=1 SV=2
h38_q1=-1
h38_q2=0,586.343842,0.045405,490,494,0.00,LQQAK,0,0000000,0.00,1,0000000000000000000,0,0,0.000000
h38_q2_terms=R,Q
h38_q3=-1
h38_q4=-1
h38_q5=-1
h38_q6=0,744.376587,0.020060,355,360,0.00,QAEIQR,0,01000000,0.00,1,0000000000000000000,0,0,0.000000
h38_q6_terms=R,K
h38_q7=-1
h38_q8=-1
h38_q9=-1
h38_q10=-1
h38_q11=-1
h38_q12=-1
h38_q13=2,985.451172,0.063414,477,483,0.00,NRKDHCR,0,010000000,0.00,1,0000000000000000000,0,0,0.000000
h38_q13_terms=R,E
h38_q14=0,1130.524384,0.010929,17,25,0.00,EDLFPEPER,0,00000000000,0.00,1,0000000000000000000,0,0,0.000000
h38_q14_terms=R,H
h38_q15=1,1478.708847,0.015516,316,328,0.00,RFGQAAWATAMPR,0,000010000002000,0.00,1,0000000000000000000,0,0,0.000000
h38_q15_terms=K,L
h38_q16=1,1553.846649,-0.002552,26,38,0.00,HQLRFLVAWNAAK,0,001000000000000,0.00,1,0000000000000000000,0,0,0.000000
h38_q16_terms=R,G
h38_q17=2,1582.831436,-0.064619,355,367,0.00,QAEIQRKVEDLPR,0,010001000000000,0.00,1,0000000000000000000,0,0,0.000000
h38_q17_terms=R,Q
h38_q18=1,1901.023148,-0.105425,187,203,0.00,LGQTATRLHQVLQDHGK,0,0000000000000000000,0.00,1,0000000000000000000,0,0,0.000000
h38_q18_terms=R,A
h38_q19=-1
h38_q20=-1
h38_q21=1,2149.113602,0.144814,194,212,0.00,LHQVLQDHGKANTMVALMK,0,000000000000002000000,0.00,1,0000000000000000000,0,0,0.000000
h38_q21_terms=R,V
h38_q22=-1
h39=TFE3_MOUSE,3.00e+001,0.27,61555.70
h39_text=Transcription factor E3 OS=Mus musculus OX=10090 GN=Tfe3 PE=1 SV=2
h39_q1=-1
h39_q2=-1
h39_q3=-1
h39_q4=0,675.337372,-0.003404,117,121,0.00,QQLMR,0,0100000,0.00,1,0000000000000000000,0,0,0.000000
h39_q4_terms=R,A
h39_q5=2,687.402740,0.040868,343,347,0.00,ERQKK,0,0000000,0.00,1,0000000000000000000,0,0,0.000000
h39_q5_terms=K,D
h39_q6=-1
h39_q7=-1
h39_q8=-1
h39_q9=0,859.491577,-0.064613,69,75,0.00,SQPLFLR,0,000000000,0.00,1,0000000000000000000,0,0,0.000000
h39_q9_terms=K,S
h39_q10=-1
h39_q11=-1
h39_q12=-1
h39_q13=2,985.591980,-0.077394,339,346,0.00,ALLKERQK,0,0000000100,0.00,1,0000000000000000000,0,0,0.000000
h39_q13_terms=K,K
h39_q14=-1
h39_q15=-1
h39_q16=2,1553.853867,-0.009770,188,199,0.00,YHLQQARRQQVK,0,00000000000000,0.00,1,0000000000000000000,0,0,0.000000
h39_q16_terms=R,Q
h39_q17=-1
h39_q18=-1
h39_q19=-1
h39_q20=-1
h39_q21=2,2149.098312,0.160104,103,121,0.00,TPAMSSSSSRVLLRQQLMR,0,000000000000000110000,0.00,1,0000000000000000000,0,0,0.000000
h39_q21_terms=R,A
h39_q22=-1
h40=CHST7_MOUSE,2.99e+001,0.32,55302.81
h40_text=Carbohydrate sulfotransferase 7 OS=Mus musculus OX=10090 GN=Chst7 PE=2 SV=1
h40_q1=-1
h40_q2=0,586.343842,0.045405,290,294,0.00,QGLLR,0,0100000,0.00,1,0000000000000000000,0,0,0.000000
h40_q2_terms=R,E
h40_q3=-1
h40_q4=-1
h40_q5=-1
h40_q6=-1
h40_q7=1,754.343185,-0.012924,9,13,0.00,REYCK,0,0000000,0.00,1,0000000000000000000,0,0,0.000000
h40_q7_terms=R,F
h40_q8=-1
h40_q9=0,859.439926,-0.012962,436,442,0.00,LSQEQVR,0,000100000,0.00,1,0000000000000000000,0,0,0.000000
h40_q9_terms=R,Q
h40_q10=-1
h40_q11=1,925.524597,-0.058697,359,366,0.00,GAPAWLRR,0,0000000000,0.00,1,0000000000000000000,0,0,0.000000
h40_q11_terms=R,R
h40_q12=-1
h40_q13=-1
h40_q14=-1
h40_q15=-1
h40_q16=-1
h40_q17=-1
h40_q18=2,1900.953033,-0.035310,468,484,0.00,TVREGETPLETKANWAV,0,0000000000000010000,0.00,1,0000000000000000000,0,0,0.000000
h40_q18_terms=K,-
h40_q19=-1
h40_q20=1,1916.963181,-0.043674,371,385,0.00,LRYEDLVWQPQAQLR,0,00000000010101000,0.00,1,0000000000000000000,0,0,0.000000
h40_q20_terms=R,R
h40_q21=-1
h40_q22=2,2186.148346,-0.051804,373,389,0.00,YEDLVWQPQAQLRRLLR,0,0000000101010000000,0.00,1,0000000000000000000,0,0,0.000000
h40_q22_terms=R,F
h41=PRS35_RAT,2.98e+001,0.27,45923.58
h41_text=Inactive serine protease 35 OS=Rattus norvegicus OX=10116 GN=Prss35 PE=2 SV=1
h41_q1=-1
h41_q2=-1
h41_q3=-1
h41_q4=-1
h41_q5=2,687.439133,0.004475,177,182,0.00,GSKKLR,0,00000000,0.00,1,0000000000000000000,0,0,0.000000
h41_q5_terms=K,V
h41_q6=-1
h41_q7=-1
h41_q8=0,836.402817,0.054856,377,383,0.00,DYNVAVR,0,000100000,0.00,1,0000000000000000000,0,0,0.000000
h41_q8_terms=K,I
h41_q9=-1
h41_q10=-1
h41_q11=0,925.414124,0.051776,120,127,0.00,QVYGTDSR,0,0100000000,0.00,1,0000000000000000000,0,0,0.000000
h41_q11_terms=R,F
h41_q12=-1
h41_q13=1,985.498108,0.016478,349,356,0.00,EPGQKNWK,0,0000000000,0.00,1,0000000000000000000,0,0,0.000000
h41_q13_terms=K,R
h41_q14=-1
h41_q15=-1
h41_q16=1,1553.835449,0.008648,134,146,0.00,RFLTNFPFNTAVK,0,000000000000000,0.00,1,0000000000000000000,0,0,0.000000
h41_q16_terms=K,L
h41_q17=-1
h41_q18=-1
h41_q19=-1
h41_q20=-1
h41_q21=1,2149.196732,0.061684,97,116,0.00,VQGLVLEPTQNSSIKGARPR,0,0000000000000000000000,0.00,1,0000000000000000000,0,0,0.000000
h41_q21_terms=K,R
h41_q22=-1
h42=MMP21_MOUSE,2.98e+001,0.32,65698.08
h42_text=Matrix metalloproteinase-21 OS=Mus musculus OX=10090 GN=Mmp21 PE=1 SV=1
h42_q1=1,577.333618,-0.032786,86,89,0.00,RFQK,0,000000,0.00,1,0000000000000000000,0,0,0.000000
h42_q1_terms=R,A
h42_q2=-1
h42_q3=-1
h42_q4=0,675.370407,-0.036439,174,178,0.00,TLTWR,0,0000000,0.00,1,0000000000000000000,0,0,0.000000
h42_q4_terms=K,L
h42_q5=-1
h42_q6=-1
h42_q7=0,754.328583,0.001678,522,527,0.00,GNSYWK,0,00100000,0.00,1,0000000000000000000,0,0,0.000000
h42_q7_terms=K,V
h42_q8=-1
h42_q9=0,859.494934,-0.067970,1,8,0.00,MLAASVLR,0,0000000000,0.00,1,0000000000000000000,0,0,0.000000
h42_q9_terms=-,L
h42_q10=-1
h42_q11=-1
h42_q12=-1
h42_q13=-1
h42_q14=-1
h42_q15=-1
h42_q16=-1
h42_q17=-1
h42_q18=0,1900.896393,0.021330,488,503,0.00,MSQVFPAIMPQNHPFR,0,000100000001000000,0.00,1,0000000000000000000,0,0,0.000000
h42_q18_terms=K,N
h42_q19=-1
h42_q20=0,1916.891312,0.028195,488,503,0.00,MSQVFPAIMPQNHPFR,0,020100000001000000,0.00,1,0000000000000000000,0,0,0.000000
h42_q20_terms=K,N
h42_q21=-1
h42_q22=0,2186.010880,0.085662,504,521,0.00,NLDSAYYSYAHNSIFFFK,0,00000000000000000000,0.00,1,0000000000000000000,0,0,0.000000
h42_q22_terms=R,G
h43=K2C3_HUMAN,2.96e+001,0.32,64548.63
h43_text=Keratin, type II cytoskeletal 3 OS=Homo sapiens OX=9606 GN=KRT3 PE=1 SV=3
h43_q1=-1
h43_q2=-1
h43_q3=-1
h43_q4=1,675.366364,-0.032396,2,7,0.00,SRQASK,0,00000000,0.00,1,0000000000000000000,0,0,0.000000
h43_q4_terms=M,T
h43_q5=-1
h43_q6=0,744.401749,-0.005102,430,436,0.00,AEIEGVK,0,000000000,0.00,1,0000000000000000000,0,0,0.000000
h43_q6_terms=R,K
h43_q7=-1
h43_q8=-1
h43_q9=-1
h43_q10=-1
h43_q11=-1
h43_q12=-1
h43_q13=-1
h43_q14=-1
h43_q15=1,1478.750397,-0.026034,218,229,0.00,FLEQQNKVLETK,0,00001110000000,0.00,1,0000000000000000000,0,0,0.000000
h43_q15_terms=R,W
h43_q16=-1
h43_q17=1,1582.769943,-0.003126,611,625,0.00,GGSIKFSQSSQSSQR,0,00000000000000000,0.00,1,0000000000000000000,0,0,0.000000
h43_q17_terms=R,Y
h43_q18=2,1900.880981,0.036742,1,19,0.00,MSRQASKTSGGGSQGFSGR,0,020000000000000000000,0.00,1,0000000000000000000,0,0,0.000000
h43_q18_terms=-,S
h43_q19=1,1910.022095,0.095088,464,480,0.00,LQELQAALQQAKDDLAR,0,0000000000000000000,0.00,1,0000000000000000000,0,0,0.000000
h43_q19_terms=K,L
h43_q20=-1
h43_q21=1,2149.101471,0.156945,387,406,0.00,AEAEALYQTKLGELQTTAGR,0,0000000000000000000000,0.00,1,0000000000000000000,0,0,0.000000
h43_q21_terms=K,H
h43_q22=-1
h44=USH1G_MOUSE,2.95e+001,0.27,51857.03
h44_text=Usher syndrome type-1G protein homolog OS=Mus musculus OX=10090 GN=Ush1g PE=1 SV=1
h44_q1=1,577.300598,0.000234,153,156,0.00,MQRK,0,020000,0.00,1,0000000000000000000,0,0,0.000000
h44_q1_terms=K,H
h44_q2=0,586.380234,0.009013,50,54,0.00,LIVSR,0,0000000,0.00,1,0000000000000000000,0,0,0.000000
h44_q2_terms=R,G
h44_q3=-1
h44_q4=-1
h44_q5=-1
h44_q6=-1
h44_q7=-1
h44_q8=-1
h44_q9=-1
h44_q10=-1
h44_q11=2,925.455185,0.010715,161,166,0.00,MERRYR,0,02000000,0.00,1,0000000000000000000,0,0,0.000000
h44_q11_terms=R,R
h44_q12=-1
h44_q13=-1
h44_q14=1,1130.521683,0.013630,2,10,0.00,NDQYHRAAR,0,01000000000,0.00,1,0000000000000000000,0,0,0.000000
h44_q14_terms=M,D
h44_q15=-1
h44_q16=-1
h44_q17=-1
h44_q18=-1
h44_q19=2,1910.022079,0.095104,446,461,0.00,RRQALERPLALEDTEL,0,000100000000000000,0.00,1,0000000000000000000,0,0,0.000000
h44_q19_terms=R,-
h44_q20=-1
h44_q21=-1
h44_q22=1,2186.071106,0.025436,410,427,0.00,HEKIDLEALMLCSDLDLR,0,00000000002000000000,0.00,1,0000000000000000000,0,0,0.000000
h44_q22_terms=R,S
h45=ACYP2_CAVPO,2.95e+001,0.18,11057.58
h45_text=Acylphosphatase-2 OS=Cavia porcellus OX=10141 GN=ACYP2 PE=1 SV=2
h45_q1=0,577.289368,0.011464,59,63,0.00,VNSMK,0,0000000,0.00,1,0000000000000000000,0,0,0.000000
h45_q1_terms=K,S
h45_q2=-1
h45_q3=-1
h45_q4=-1
h45_q5=0,687.391510,0.052098,2,8,0.00,SAAAQLK,0,000000000,0.00,1,0000000000000000000,0,0,0.000000
h45_q5_terms=M,S
h45_q6=-1
h45_q7=-1
h45_q8=-1
h45_q9=-1
h45_q10=-1
h45_q11=-1
h45_q12=-1
h45_q13=-1
h45_q14=-1
h45_q15=-1
h45_q16=-1
h45_q17=-1
h45_q18=-1
h45_q19=2,1909.997162,0.120021,25,41,0.00,MYTEGEAKKIGVVGWVK,0,0200000000000000000,0.00,1,0000000000000000000,0,0,0.000000
h45_q19_terms=R,N
h45_q20=1,1916.920349,-0.000842,9,24,0.00,SVDYEVFGRVQGVCFR,0,000000000000000000,0.00,1,0000000000000000000,0,0,0.000000
h45_q20_terms=K,M
h45_q21=-1
h45_q22=-1
h46=CAZA2_PLEMO,2.94e+001,0.23,33160.69
h46_text=F-actin-capping protein subunit alpha-2 OS=Plecturocebus moloch OX=9523 GN=CAPZA2 PE=3 SV=3
h46_q1=-1
h46_q2=-1
h46_q3=-1
h46_q4=0,675.322769,0.011199,269,273,0.00,IDWNK,0,0000100,0.00,1,0000000000000000000,0,0,0.000000
h46_q4_terms=K,I
h46_q5=-1
h46_q6=-1
h46_q7=-1
h46_q8=1,836.432678,0.024995,92,97,0.00,NRICFK,0,00000000,0.00,1,0000000000000000000,0,0,0.000000
h46_q8_terms=K,F
h46_q9=-1
h46_q10=-1
h46_q11=-1
h46_q12=1,942.549820,0.030034,261,268,0.00,QLPVTRTK,0,0100000000,0.00,1,0000000000000000000,0,0,0.000000
h46_q12_terms=R,I
h46_q13=-1
h46_q14=-1
h46_q15=0,1478.645966,0.078397,110,121,0.00,SCEVENAIESWR,0,00000000000000,0.00,1,0000000000000000000,0,0,0.000000
h46_q15_terms=R,T
h46_q16=-1
h46_q17=-1
h46_q18=-1
h46_q19=-1
h46_q20=-1
h46_q21=-1
h46_q22=0,2186.078995,0.017547,148,166,0.00,IDGQQTIIACIESHQFQAK,0,000000000000000000000,0.00,2,0000000000000000000,0,0,0.000000
h46_q22_terms=K,N
h47=CASK_RABIT,2.94e+001,0.18,20386.51
h47_text=Kappa-casein OS=Oryctolagus cuniculus OX=9986 GN=CSN3 PE=2 SV=1
h47_q1=-1
h47_q2=-1
h47_q3=-1
h47_q4=0,675.282349,0.051619,33,37,0.00,ENEER,0,0000000,0.00,1,0000000000000000000,0,0,0.000000
h47_q4_terms=R,L
h47_q5=-1
h47_q6=-1
h47_q7=-1
h47_q8=-1
h47_q9=-1
h47_q10=-1
h47_q11=-1
h47_q12=-1
h47_q13=-1
h47_q14=-1
h47_q15=0,1478.671753,0.052610,56,66,0.00,YPQYEPSYYLR,0,0001000000000,0.00,1,0000000000000000000,0,0,0.000000
h47_q15_terms=R,R
h47_q16=0,1553.781296,0.062801,111,123,0.00,HSHPFFMAILPNK,0,000000020000000,0.00,1,0000000000000000000,0,0,0.000000
h47_q16_terms=R,M
h47_q17=-1
h47_q18=-1
h47_q19=-1
h47_q20=-1
h47_q21=-1
h47_q22=0,2186.098267,-0.001725,38,55,0.00,LFHQVTAPYIPVHYVMNR,0,00001000000000000100,0.00,1,0000000000000000000,0,0,0.000000
h47_q22_terms=R,Y
h48=MTEF2_MOUSE,2.93e+001,0.27,44052.28
h48_text=Transcription termination factor 2, mitochondrial OS=Mus musculus OX=10090 GN=Mterf2 PE=1 SV=1
h48_q1=-1
h48_q2=0,586.369003,0.020244,283,287,0.00,QLVVK,0,0100000,0.00,1,0000000000000000000,0,0,0.000000
h48_q2_terms=R,C
h48_q3=-1
h48_q4=-1
h48_q5=0,687.391525,0.052083,163,168,0.00,NVVISR,0,01000000,0.00,1,0000000000000000000,0,0,0.000000
h48_q5_terms=R,F
h48_q6=-1
h48_q7=-1
h48_q8=-1
h48_q9=-1
h48_q10=-1
h48_q11=-1
h48_q12=-1
h48_q13=0,985.555618,-0.041032,309,317,0.00,EGISIAQIR,0,00000000000,0.00,1,0000000000000000000,0,0,0.000000
h48_q13_terms=K,E
h48_q14=-1
h48_q15=-1
h48_q16=-1
h48_q17=-1
h48_q18=1,1901.019272,-0.101549,366,382,0.00,MQAKQGRPLFNPVASLK,0,0210000000000000000,0.00,1,0000000000000000000,0,0,0.000000
h48_q18_terms=K,V
h48_q19=-1
h48_q20=0,1916.991714,-0.072207,318,333,0.00,ESPMVLELTPQIIQYR,0,000000000001000000,0.00,1,0000000000000000000,0,0,0.000000
h48_q20_terms=R,I
h48_q21=-1
h48_q22=2,2186.169495,-0.072953,337,357,0.00,LNSLGYGIKDGHLASLNGTKK,0,00100000000000000000000,0.00,3,0000000000000000000,0,0,0.000000
h48_q22_terms=K,E
h49=HRH2_GORGO,2.92e+001,0.23,40813.42
h49_text=Histamine H2 receptor OS=Gorilla gorilla gorilla OX=9595 GN=HRH2 PE=3 SV=1
h49_q1=0,577.270737,0.030095,162,166,0.00,NETSK,0,0000000,0.00,1,0000000000000000000,0,0,0.000000
h49_q1_terms=R,G
h49_q2=-1
h49_q3=-1
h49_q4=-1
h49_q5=-1
h49_q6=0,744.340225,0.056422,167,173,0.00,GNHTTSK,0,001000000,0.00,1,0000000000000000000,0,0,0.000000
h49_q6_terms=K,C
h49_q7=-1
h49_q8=-1
h49_q9=-1
h49_q10=-1
h49_q11=-1
h49_q12=2,942.535889,0.043965,208,215,0.00,VARDQAKR,0,0000000000,0.00,1,0000000000000000000,0,0,0.000000
h49_q12_terms=K,I
h49_q13=-1
h49_q14=-1
h49_q15=-1
h49_q16=-1
h49_q17=2,1582.821548,-0.054731,211,223,0.00,DQAKRINHISSWK,0,001000000000000,0.00,1,0000000000000000000,0,0,0.000000
h49_q17_terms=R,A
h49_q18=-1
h49_q19=-1
h49_q20=0,1916.911591,0.007916,342,359,0.00,LQVWSGTEVTAPQGATDR,0,00100000000001000000,0.00,2,0000000000000000000,0,0,0.000000
h49_q20_terms=K,-
h49_q21=-1
h49_q22=-1
h50=HRH2_HUMAN,2.92e+001,0.23,40813.42
h50_text=Histamine H2 receptor OS=Homo sapiens OX=9606 GN=HRH2 PE=2 SV=1
h50_q1=0,577.270737,0.030095,162,166,0.00,NETSK,0,0000000,0.00,1,0000000000000000000,0,0,0.000000
h50_q1_terms=R,G
h50_q2=-1
h50_q3=-1
h50_q4=-1
h50_q5=-1
h50_q6=0,744.340225,0.056422,167,173,0.00,GNHTTSK,0,001000000,0.00,1,0000000000000000000,0,0,0.000000
h50_q6_terms=K,C
h50_q7=-1
h50_q8=-1
h50_q9=-1
h50_q10=-1
h50_q11=-1
h50_q12=2,942.535889,0.043965,208,215,0.00,VARDQAKR,0,0000000000,0.00,1,0000000000000000000,0,0,0.000000
h50_q12_terms=K,I
h50_q13=-1
h50_q14=-1
h50_q15=-1
h50_q16=-1
h50_q17=2,1582.821548,-0.054731,211,223,0.00,DQAKRINHISSWK,0,001000000000000,0.00,1,0000000000000000000,0,0,0.000000
h50_q17_terms=R,A
h50_q18=-1
h50_q19=-1
h50_q20=0,1916.911591,0.007916,342,359,0.00,LQVWSGTEVTAPQGATDR,0,00100000000001000000,0.00,2,0000000000000000000,0,0,0.000000
h50_q20_terms=K,-
h50_q21=-1
h50_q22=-1
--gc0p4Jq0M2Yt08jU534c0p
Content-Type: application/x-Mascot; name="mixture"

num_hits=0
--gc0p4Jq0M2Yt08jU534c0p
Content-Type: application/x-Mascot; name="query1"

charge=1+
--gc0p4Jq0M2Yt08jU534c0p
Content-Type: application/x-Mascot; name="query2"

charge=1+
--gc0p4Jq0M2Yt08jU534c0p
Content-Type: application/x-Mascot; name="query3"

charge=1+
--gc0p4Jq0M2Yt08jU534c0p
Content-Type: application/x-Mascot; name="query4"

charge=1+
--gc0p4Jq0M2Yt08jU534c0p
Content-Type: application/x-Mascot; name="query5"

charge=1+
--gc0p4Jq0M2Yt08jU534c0p
Content-Type: application/x-Mascot; name="query6"

charge=1+
--gc0p4Jq0M2Yt08jU534c0p
Content-Type: application/x-Mascot; name="query7"

charge=1+
--gc0p4Jq0M2Yt08jU534c0p
Content-Type: application/x-Mascot; name="query8"

charge=1+
--gc0p4Jq0M2Yt08jU534c0p
Content-Type: application/x-Mascot; name="query9"

charge=1+
--gc0p4Jq0M2Yt08jU534c0p
Content-Type: application/x-Mascot; name="query10"

charge=1+
--gc0p4Jq0M2Yt08jU534c0p
Content-Type: application/x-Mascot; name="query11"

charge=1+
--gc0p4Jq0M2Yt08jU534c0p
Content-Type: application/x-Mascot; name="query12"

charge=1+
--gc0p4Jq0M2Yt08jU534c0p
Content-Type: application/x-Mascot; name="query13"

charge=1+
--gc0p4Jq0M2Yt08jU534c0p
Content-Type: application/x-Mascot; name="query14"

charge=1+
--gc0p4Jq0M2Yt08jU534c0p
Content-Type: application/x-Mascot; name="query15"

charge=1+
--gc0p4Jq0M2Yt08jU534c0p
Content-Type: application/x-Mascot; name="query16"

charge=1+
--gc0p4Jq0M2Yt08jU534c0p
Content-Type: application/x-Mascot; name="query17"

charge=1+
--gc0p4Jq0M2Yt08jU534c0p
Content-Type: application/x-Mascot; name="query18"

charge=1+
--gc0p4Jq0M2Yt08jU534c0p
Content-Type: application/x-Mascot; name="query19"

charge=1+
--gc0p4Jq0M2Yt08jU534c0p
Content-Type: application/x-Mascot; name="query20"

charge=1+
--gc0p4Jq0M2Yt08jU534c0p
Content-Type: application/x-Mascot; name="query21"

charge=1+
--gc0p4Jq0M2Yt08jU534c0p
Content-Type: application/x-Mascot; name="query22"

charge=1+
--gc0p4Jq0M2Yt08jU534c0p
Content-Type: application/x-Mascot; name="index"

parameters=4
masses=79
unimod=122
enzyme=556
taxonomy=564
header=571
summary=590
mixture=2252
query1=2256
query2=2260
query3=2264
query4=2268
query5=2272
query6=2276
query7=2280
query8=2284
query9=2288
query10=2292
query11=2296
query12=2300
query13=2304
query14=2308
query15=2312
query16=2316
query17=2320
query18=2324
query19=2328
query20=2332
query21=2336
query22=2340
--gc0p4Jq0M2Yt08jU534c0p--
```
