# Supplementary figures and images for: Monoterpenoid aryl hydrocarbon receptor allosteric antagonists protect against ultraviolet skin damage in female mice
Source: Nat Commun. 2023 May 11;14:2728. doi: 10.1038/s41467-023-38478-6 (PMC10174618; doi:10.1038/s41467-023-38478-6)

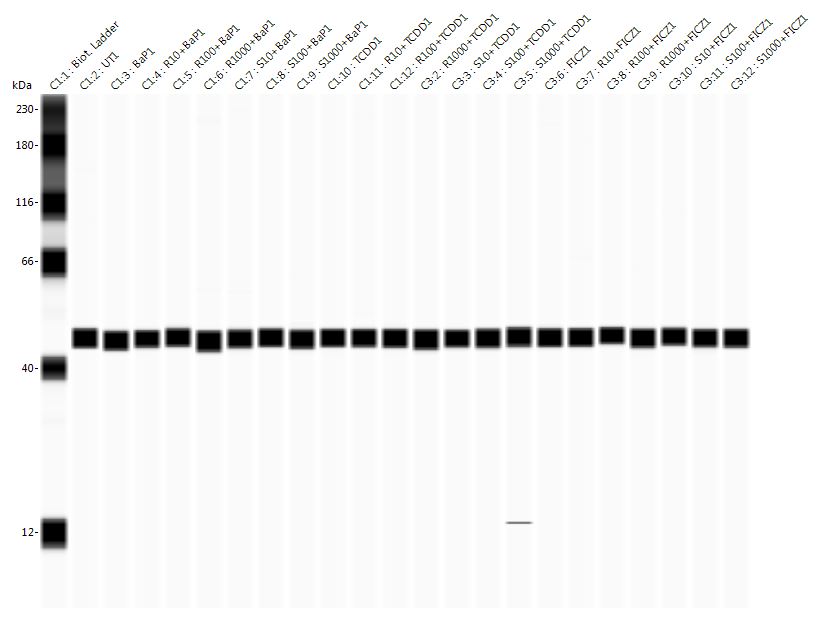

Supplement: Supplementary file 4 — source data [file 41467_2023_38478_MOESM4_ESM.zip › DATA - ONDROVA new/Figure 2/Figure 2B-HaCaT_actin.JPG]

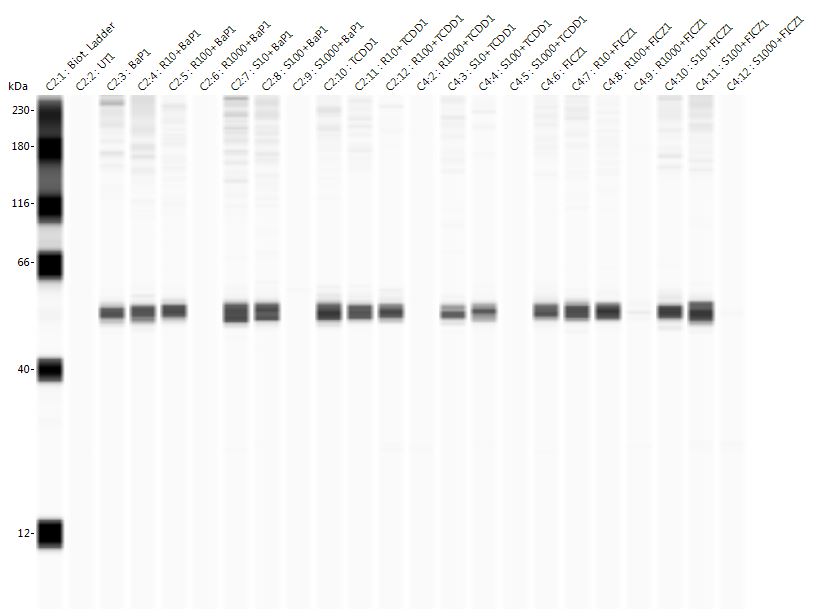

Supplement: Supplementary file 4 — source data [file 41467_2023_38478_MOESM4_ESM.zip › DATA - ONDROVA new/Figure 2/Figure 2B-HaCaT_CYP1A1.JPG]

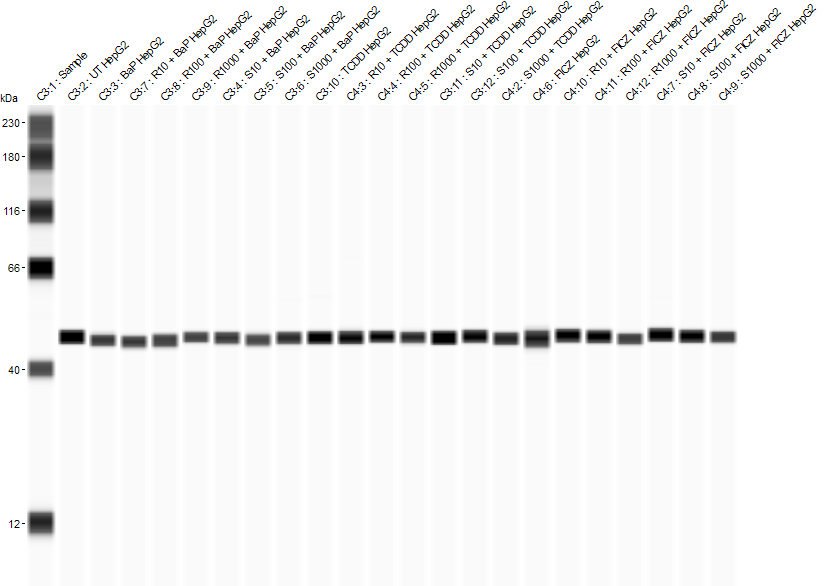

Supplement: Supplementary file 4 — source data [file 41467_2023_38478_MOESM4_ESM.zip › DATA - ONDROVA new/Figure 2/Figure 2B-HepG2_actin.PNG]

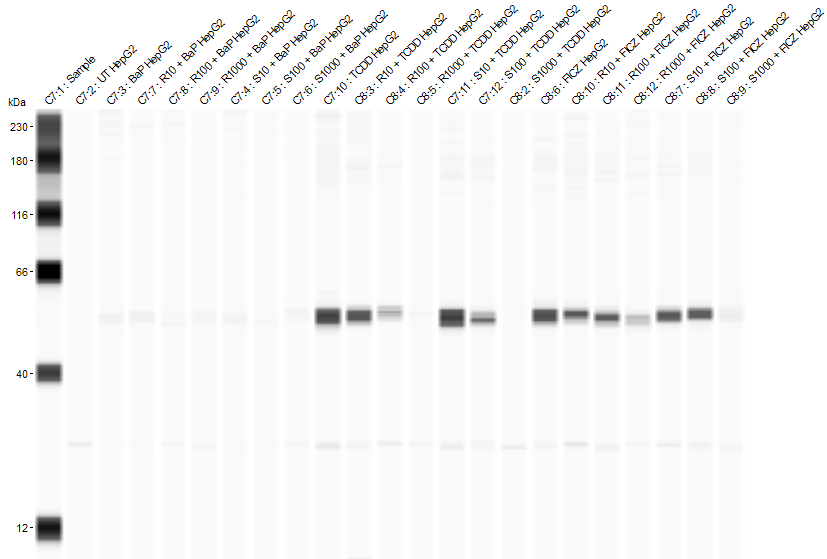

Supplement: Supplementary file 4 — source data [file 41467_2023_38478_MOESM4_ESM.zip › DATA - ONDROVA new/Figure 2/Figure 2B-HepG2_CYP1A1.PNG]

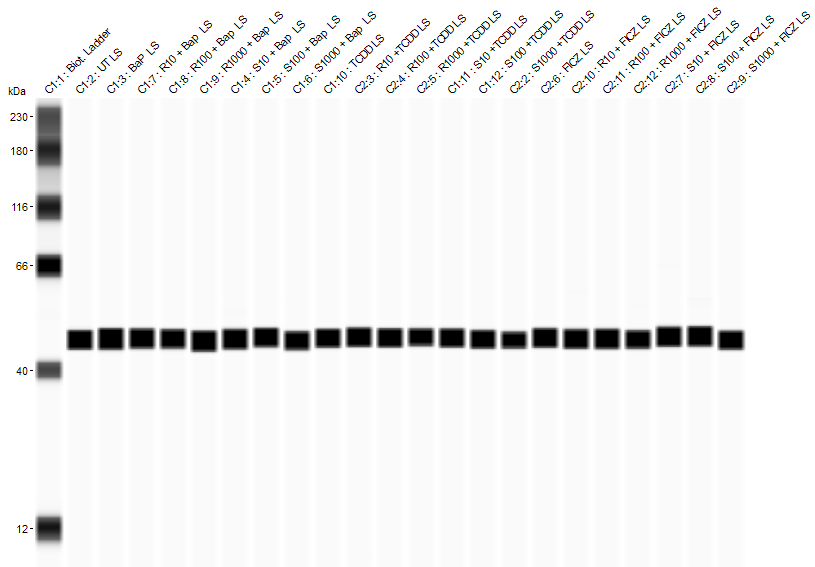

Supplement: Supplementary file 4 — source data [file 41467_2023_38478_MOESM4_ESM.zip › DATA - ONDROVA new/Figure 2/Figure 2B-LS180_actin.PNG]

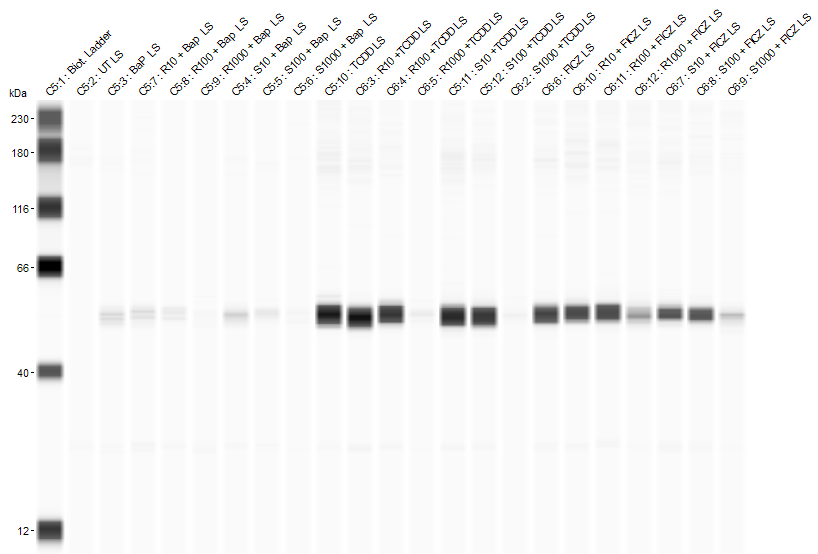

Supplement: Supplementary file 4 — source data [file 41467_2023_38478_MOESM4_ESM.zip › DATA - ONDROVA new/Figure 2/Figure 2B-LS180_CYP1A1.PNG]

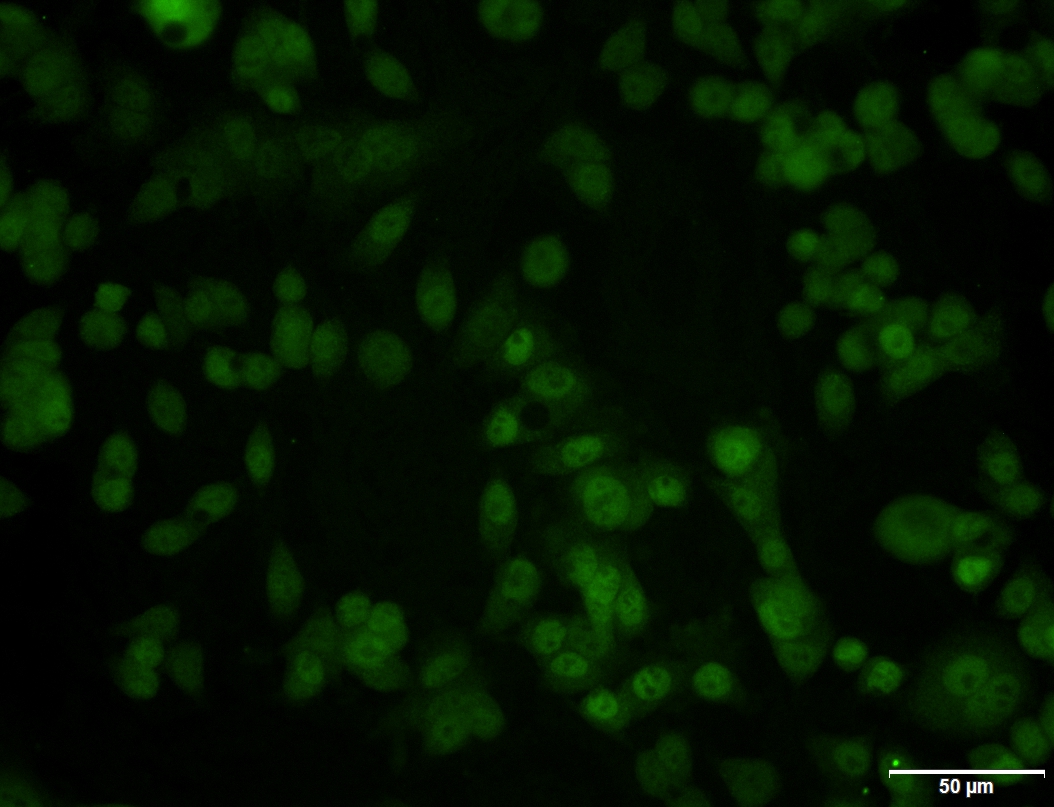

Supplement: Supplementary file 4 — source data [file 41467_2023_38478_MOESM4_ESM.zip › DATA - ONDROVA new/Figure 3/Figure 3A/2018-07-19_tcdd.jpg]

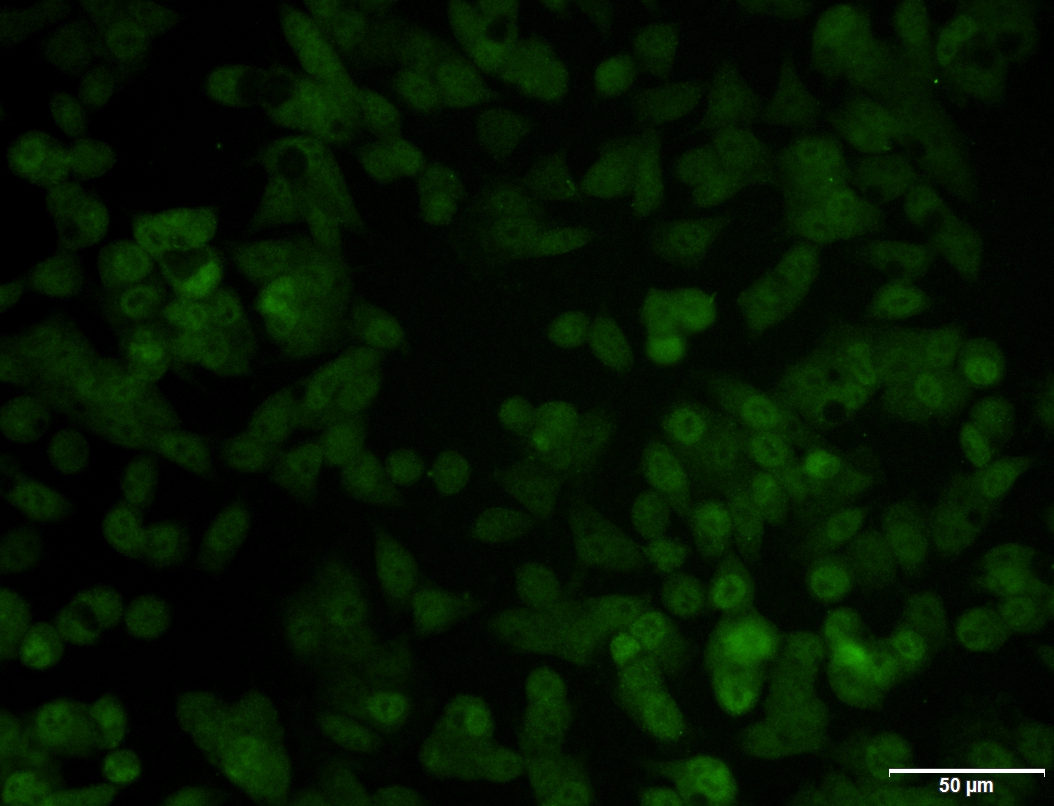

Supplement: Supplementary file 4 — source data [file 41467_2023_38478_MOESM4_ESM.zip › DATA - ONDROVA new/Figure 3/Figure 3A/2018-07-20_ficz.jpg]

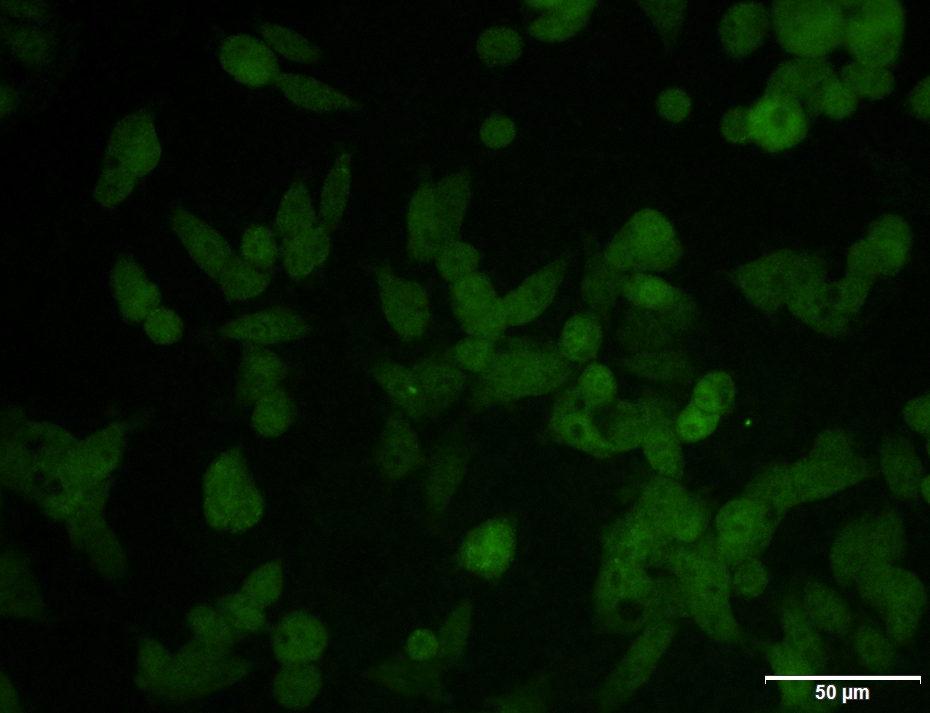

Supplement: Supplementary file 4 — source data [file 41467_2023_38478_MOESM4_ESM.zip › DATA - ONDROVA new/Figure 3/Figure 3A/2018-07-24_1000uM_RC.jpg]

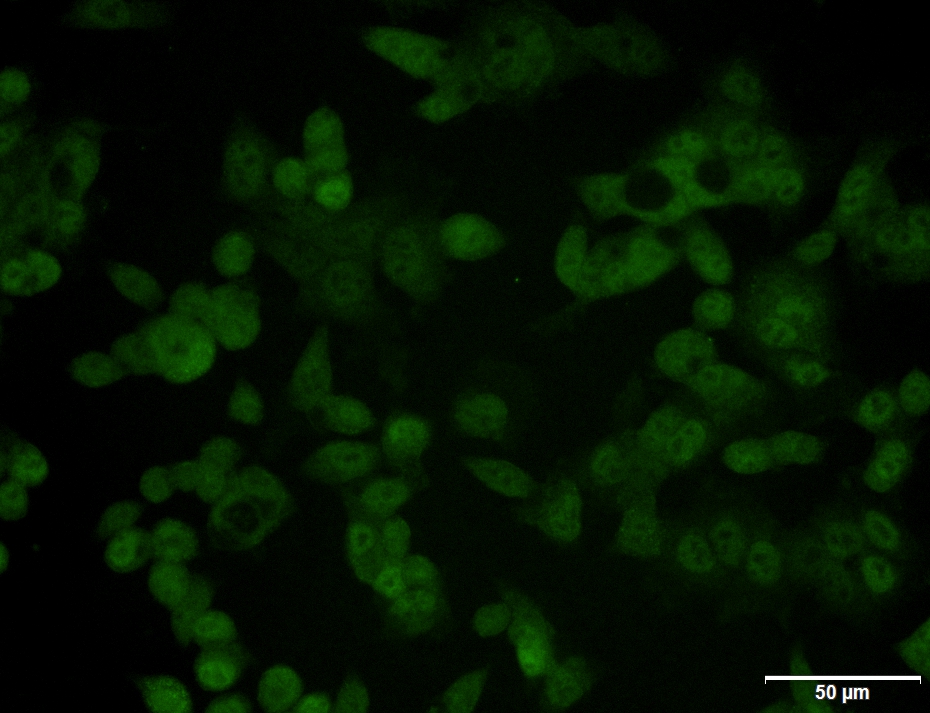

Supplement: Supplementary file 4 — source data [file 41467_2023_38478_MOESM4_ESM.zip › DATA - ONDROVA new/Figure 3/Figure 3A/2018-07-24_1000uM_RC_bap.jpg]

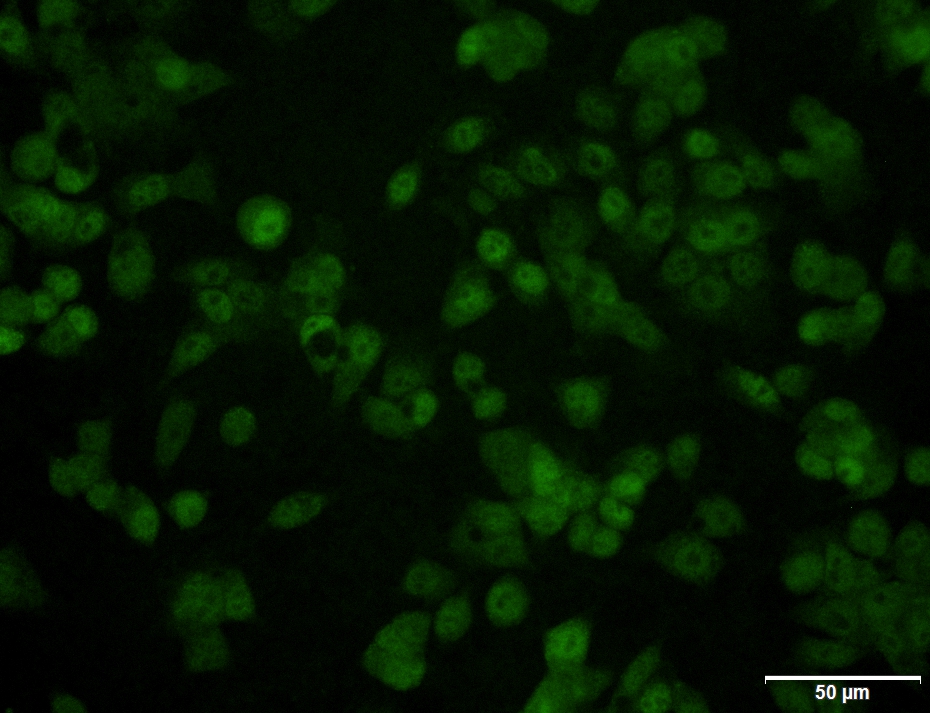

Supplement: Supplementary file 4 — source data [file 41467_2023_38478_MOESM4_ESM.zip › DATA - ONDROVA new/Figure 3/Figure 3A/2018-07-24_1000uM_RC_ficz.jpg]

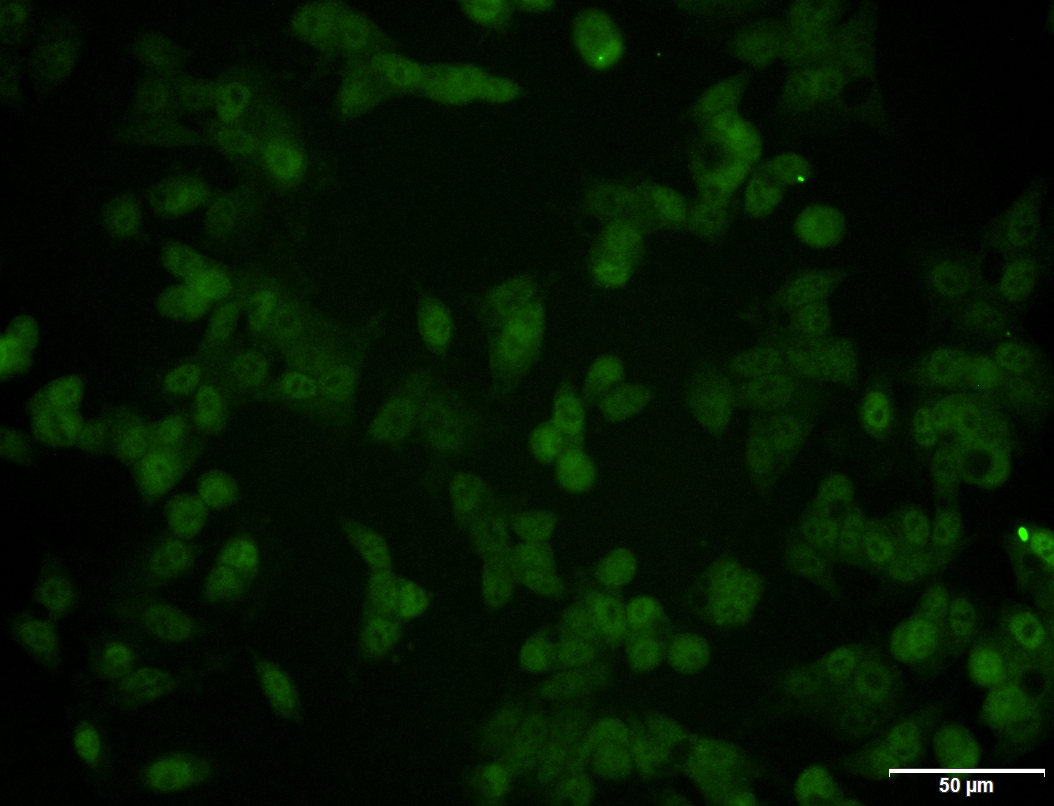

Supplement: Supplementary file 4 — source data [file 41467_2023_38478_MOESM4_ESM.zip › DATA - ONDROVA new/Figure 3/Figure 3A/2018-07-24_1000uM_RC_tcdd.jpg]

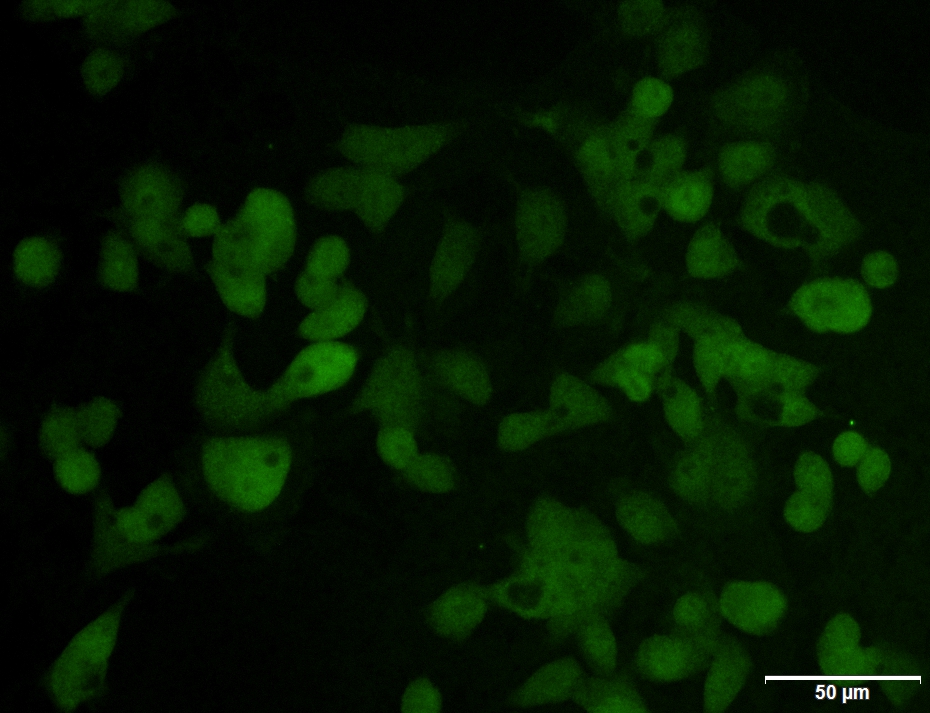

Supplement: Supplementary file 4 — source data [file 41467_2023_38478_MOESM4_ESM.zip › DATA - ONDROVA new/Figure 3/Figure 3A/2018-07-24_1000uM_SC.jpg]

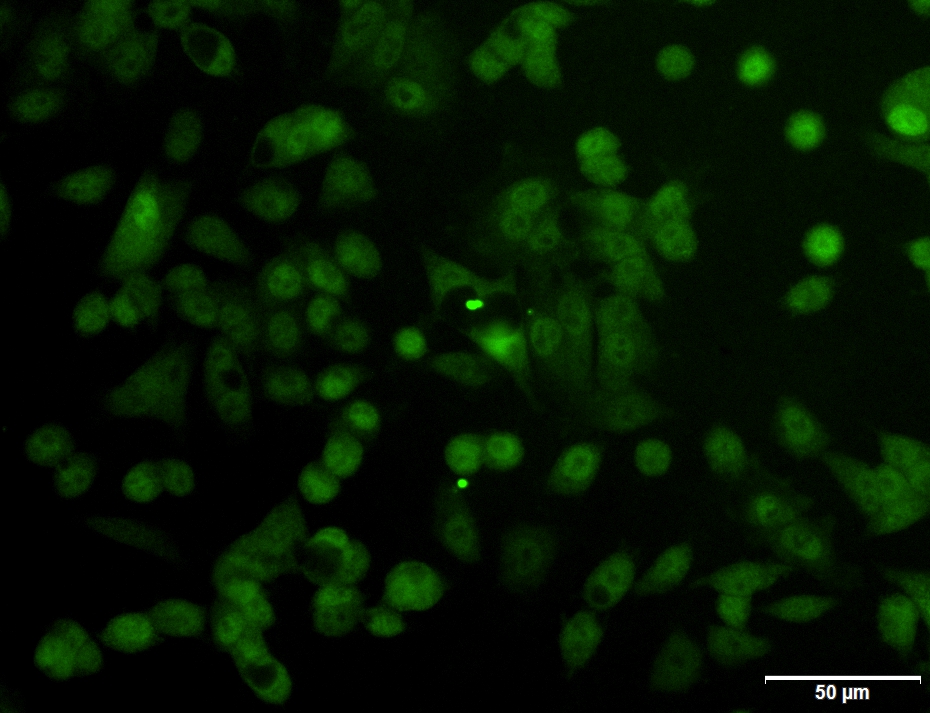

Supplement: Supplementary file 4 — source data [file 41467_2023_38478_MOESM4_ESM.zip › DATA - ONDROVA new/Figure 3/Figure 3A/2018-07-24_1000uM_SC_bap.jpg]

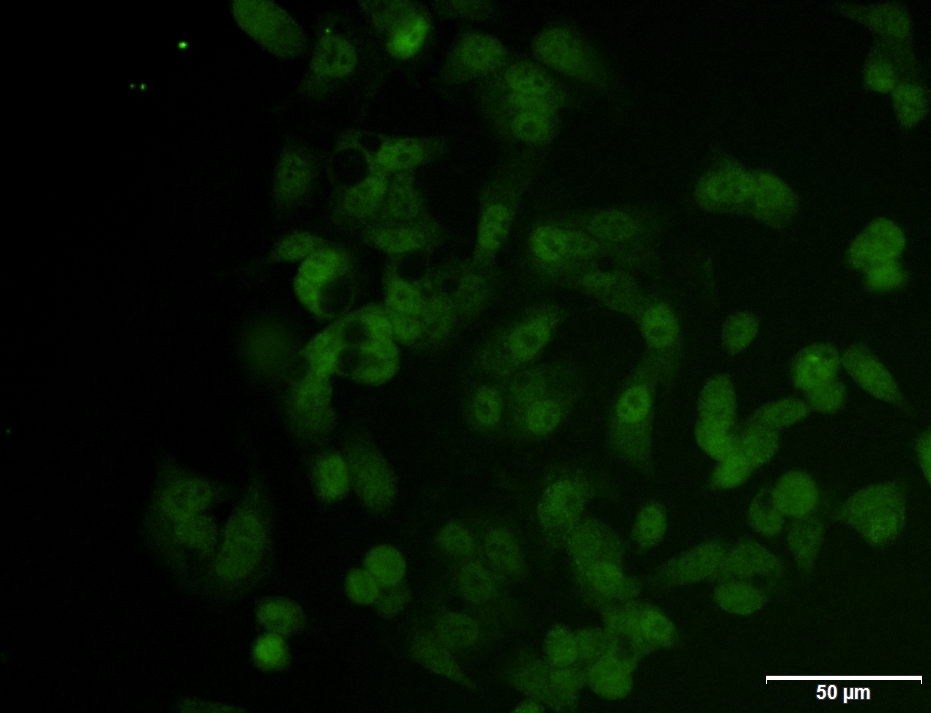

Supplement: Supplementary file 4 — source data [file 41467_2023_38478_MOESM4_ESM.zip › DATA - ONDROVA new/Figure 3/Figure 3A/2018-07-24_1000uM_SC_ficz.jpg]

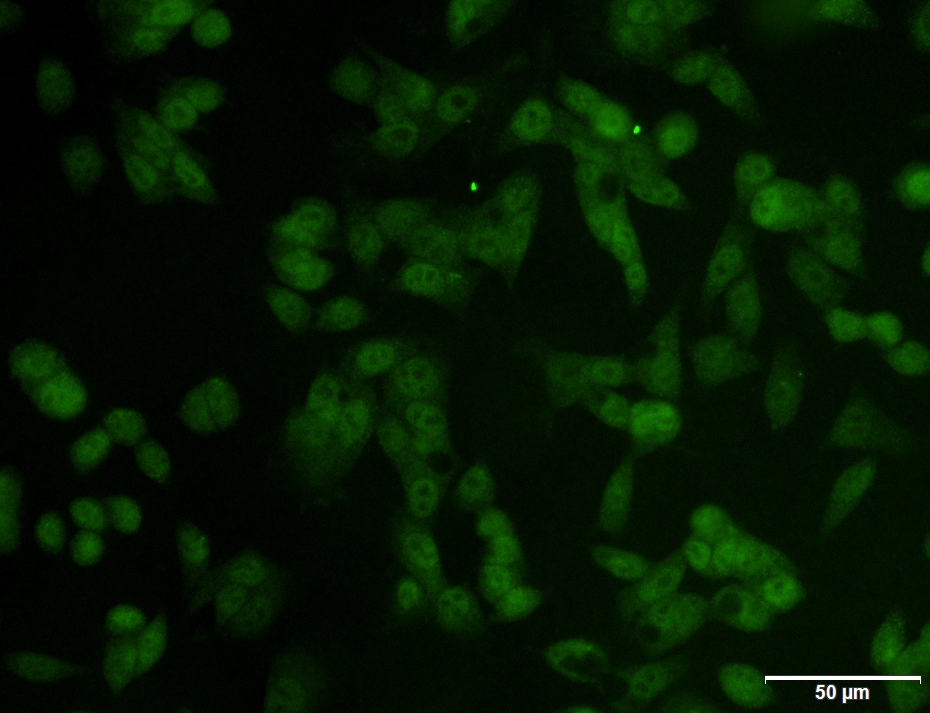

Supplement: Supplementary file 4 — source data [file 41467_2023_38478_MOESM4_ESM.zip › DATA - ONDROVA new/Figure 3/Figure 3A/2018-07-24_1000uM_SC_tcdd.jpg]

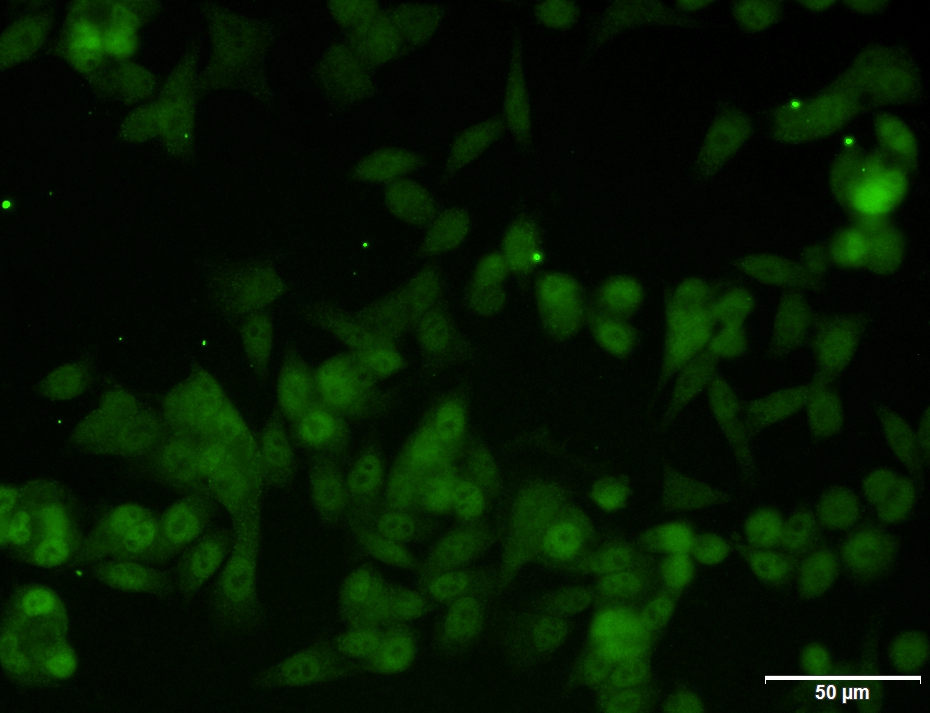

Supplement: Supplementary file 4 — source data [file 41467_2023_38478_MOESM4_ESM.zip › DATA - ONDROVA new/Figure 3/Figure 3A/2018-07-31_ BaP.jpg]

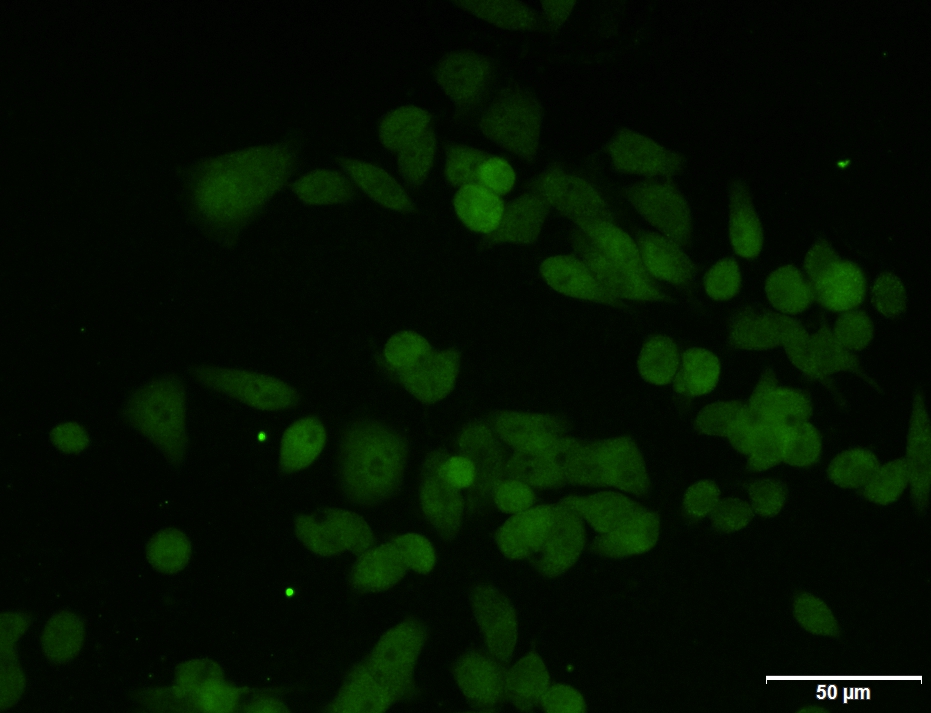

Supplement: Supplementary file 4 — source data [file 41467_2023_38478_MOESM4_ESM.zip › DATA - ONDROVA new/Figure 3/Figure 3A/2018-07-31_dmso.jpg]

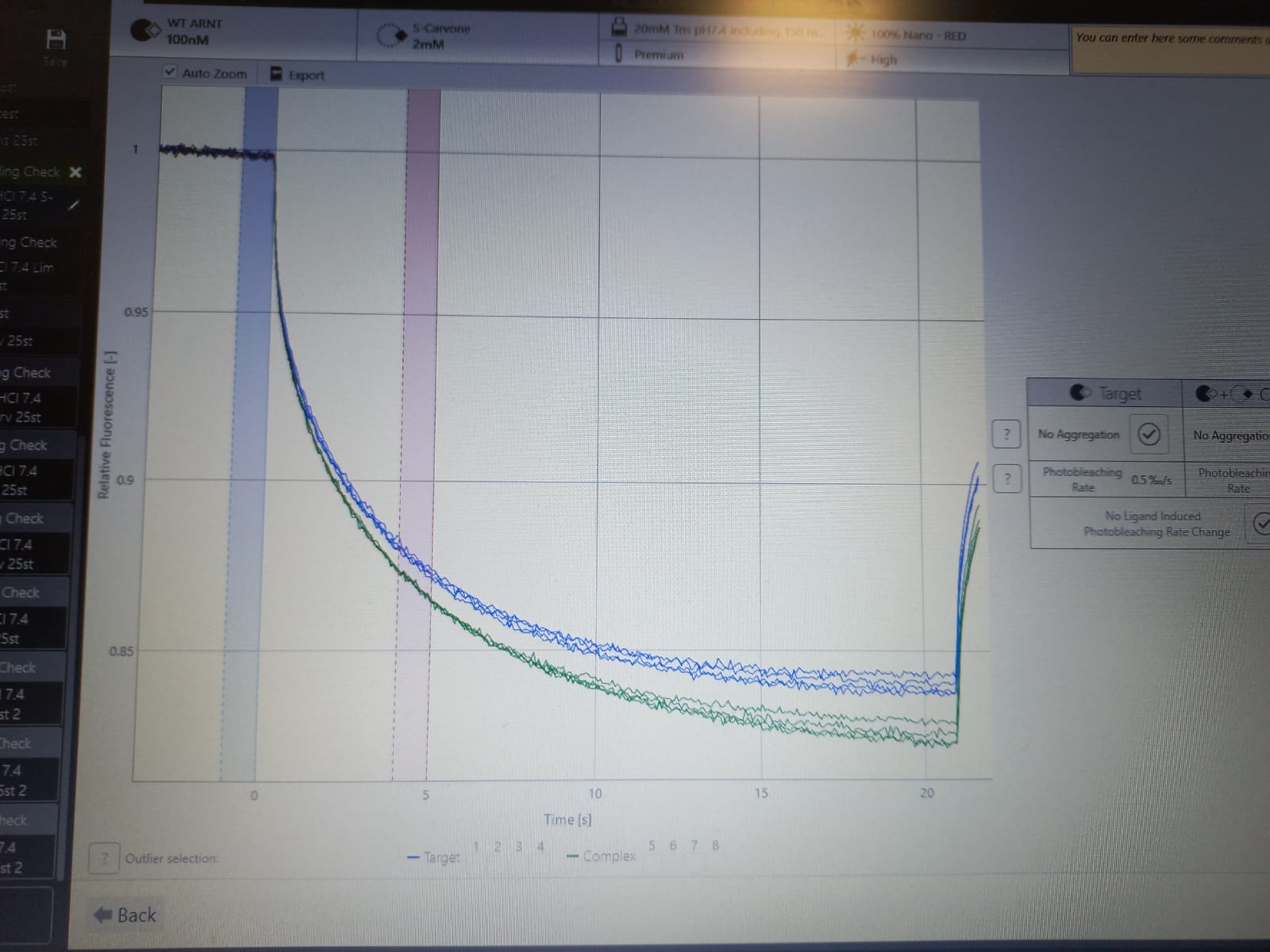

Supplement: Supplementary file 4 — source data [file 41467_2023_38478_MOESM4_ESM.zip › DATA - ONDROVA new/Figure 4/Figure 4D/Figure 4D - WT.jpg]

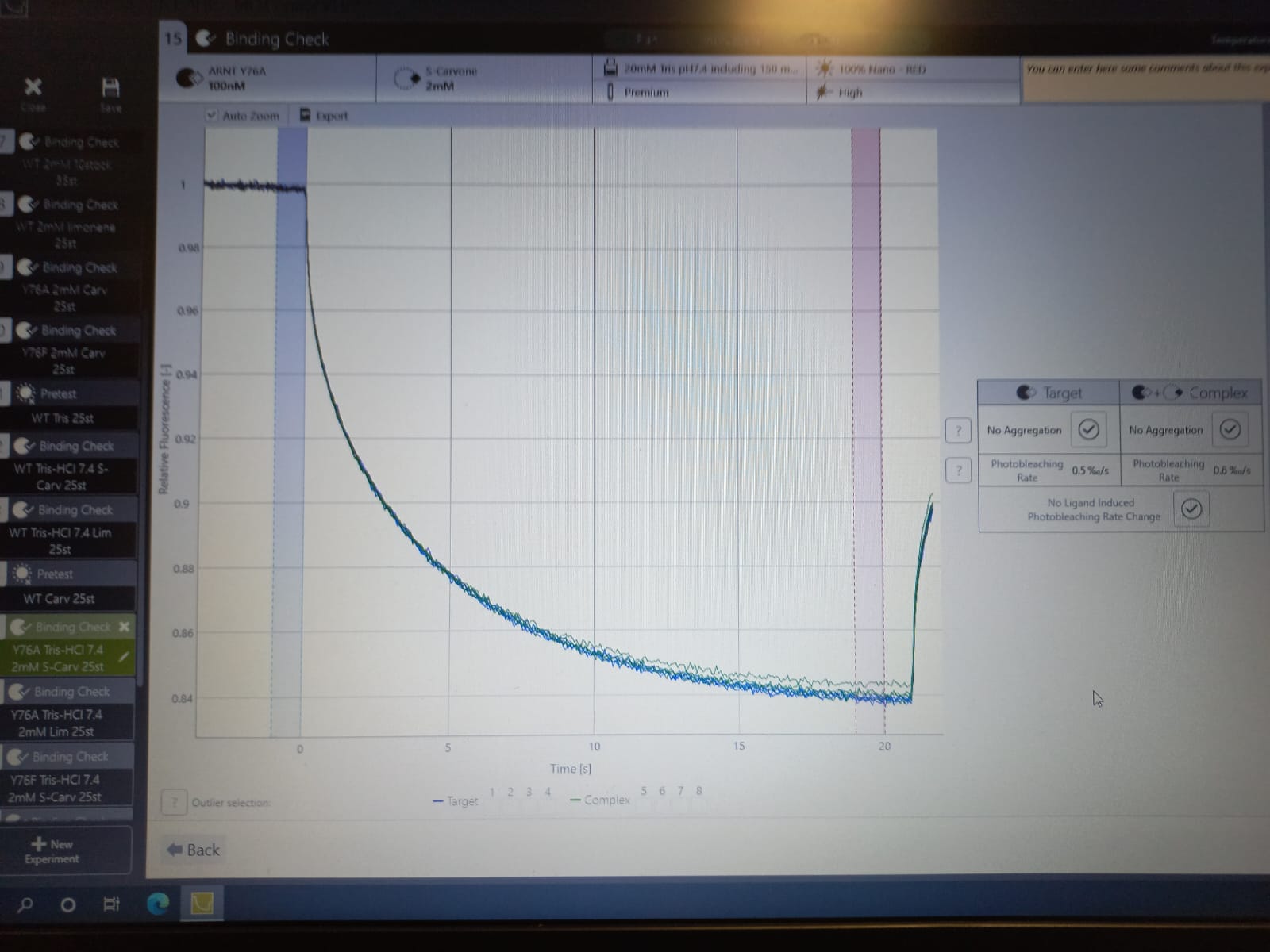

Supplement: Supplementary file 4 — source data [file 41467_2023_38478_MOESM4_ESM.zip › DATA - ONDROVA new/Figure 4/Figure 4D/Figure 4D - Y76A.jpg]

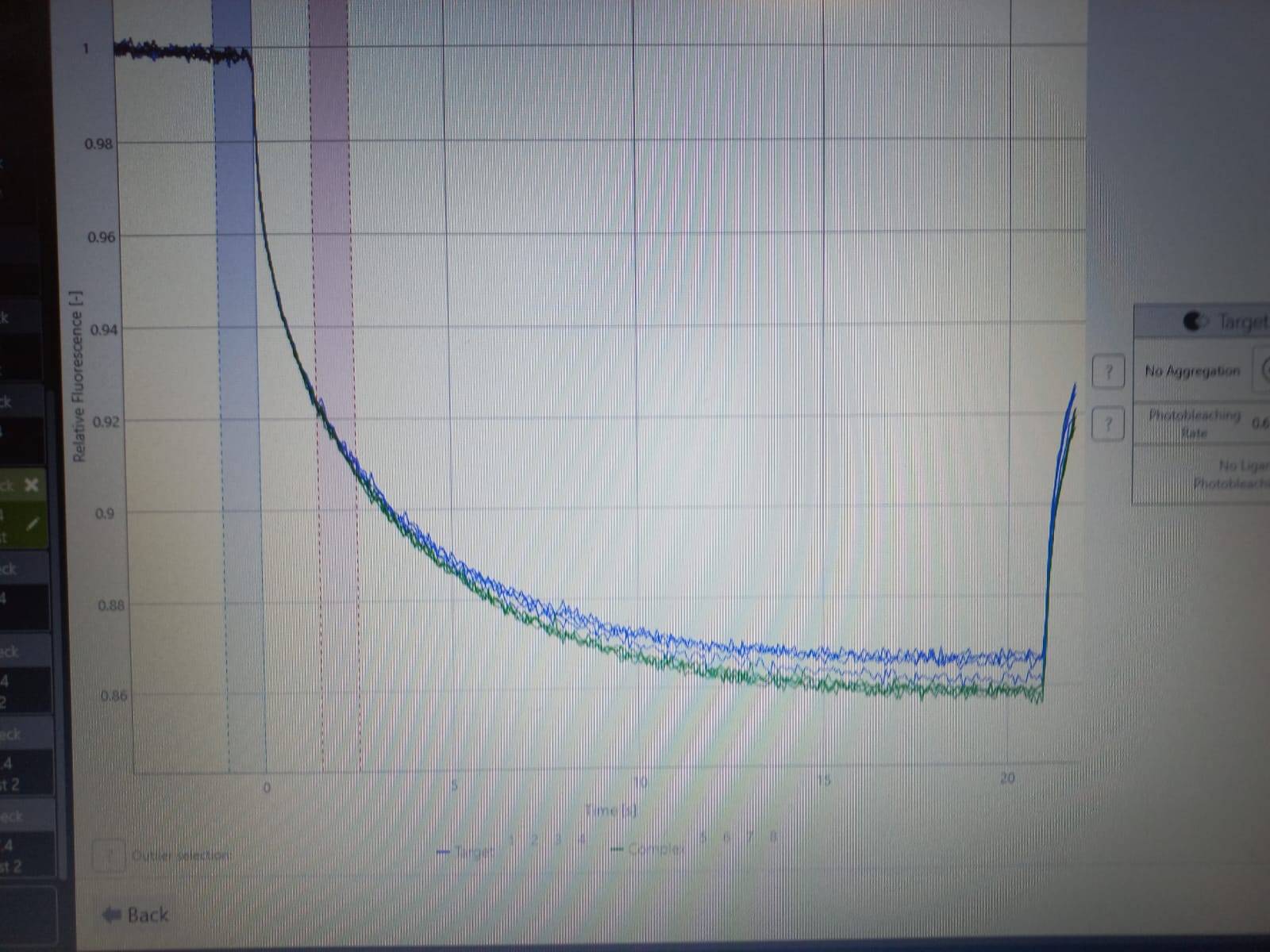

Supplement: Supplementary file 4 — source data [file 41467_2023_38478_MOESM4_ESM.zip › DATA - ONDROVA new/Figure 4/Figure 4D/Figure 4D - Y76F.jpg]

## Slide 1
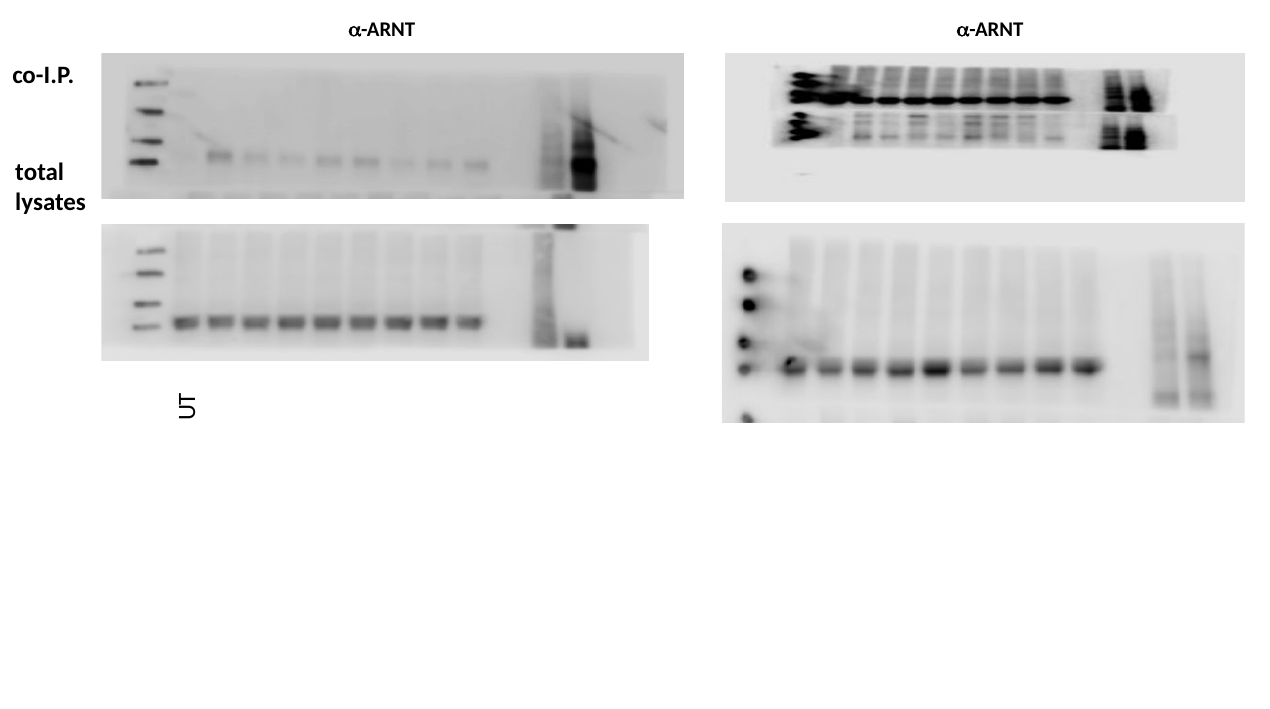

a-ARNT
a-ARNT
co-I.P.
total
lysates
UT

Supplement: Supplementary file 4 — source data [file 41467_2023_38478_MOESM4_ESM.zip › DATA - ONDROVA new/Figure S5/Figure S5B/Figure S5B.pptx]
